# Tanned leather of the paiche *Arapaima gigas* Schinz, 1822 (Arapaimidae) with extracts of vegetable origin to replace chromium salts

**DOI:** 10.1371/journal.pone.0261781

**Published:** 2022-01-21

**Authors:** Jucilene Cavali, Maria Luiza Rodrigues de Souza, Patrícia Silva de Oliveira Kanarski, Melina Franco Coradini, Jerônimo Vieira Dantas Filho

**Affiliations:** 1 Programa de Pós-Graduação em Ciências Ambientais, Universidade Federal de Rondônia (UNIR), Rolim de Moura, Rondônia, Brazil; 2 Laboratório de Processamentos de Peles e Couros, Universidade Estadual de Maringá (UEM), Maringá, Paraná, Brazil; Massey University, NEW ZEALAND

## Abstract

With the intensification of fish farming, the amount of residues also increased. One of the by-products generated is leather. However, several factors influence its quality, among them, the types of tanning used. Paiche (*Arapaima gigas*) is the largest freshwater fish in the world, and therefore has great potential in the use of leather, in addition to being one of the most cultivated fish in the Rondônia state. The aimed was to evaluate the physicomechanical resistance, histological and morphological aspects in different directions of the fibers of the paiche to evaluate the tanning with chromium oxide and vegetable tannin. Paiches with an average weight of 12.0 kg were used, skins were made and tanned using chromium and vegetable tannin as techniques. After tanning, 20 specimens were removed in longitudinal, transverse and diagonal directions to the fish body, to determine resistance in dynamometer and leather for histological analysis, scanning electron microscopy and physical-chemical analysis. The average thickness of the specimens of the leathers ranged 1.79mm to 2.82mm, in addition, there was no interaction between the type of tanning agent and the directions obtained for strength, traction and elongation. Regarding the progressive tearing test, there was also no interaction effect. However, the defined factors had relevant differences for the maximum and average amount applied, and the leathers tanned with vegetable tannin expanded to larger dimensions 110.19 and 85.52 N. According to the images obtained by histology and scanning microscopy, they presented that in the longitudinal and transverse direction the collagen fibers are presented in layers parallel to the leather surface and in the diagonal direction the interlacing is more intense, that is, in addition to the overlapping layers intercalated thinner, close to the surface, fiber bundles can also be seen crossing each other. It is concluded that leathers tanned with vegetable tannin have less resistance than leathers tanned with chromium salts for traction to rupture and greater resistance to tear.

## Introduction

The demand to increase the production of food for human consumption has made efforts to make viable alternatives in quantity and quality, and in this sense, aquaculture has been shown to be adequate for this purpose [1, 2]. Therefore, the development of fish farming in Brazil is due to factors such as investments in the production and processing sector, the increase in research, together with the vast available water resources [3]. And among the cultivated native fish species, the paiche *Arapaima gigas* Schinz, 1822 (Osteoglossiformes: Arapaimidae) has been gaining great interest, due to its meat quality and excellent acceptance by consumers [4].

Fish skin can vary between 4.5 to 14.0% in relation to body weight, and the skin can be transformed into leather [5]. The skin processed into leather can later be used as a raw material for making shoes, bags, wallets, belts, jackets, among others [6]. To carry out the tanning of fish skins, whatever the species, several types of tanning agents can be used, including chromium salts, vegetable or synthetic tannin, aluminum, as well as for dyeing, the use of special chemical dyes for leather or natural fabrics from different origins [7]. The use of tanning agents of mineral origin has been neglected, as is the case with chromium, as they can be harmful to the environment, leading many countries to give commercial preference to leathers that used vegetable tannins [8].

Chromium is the most used mineral agent worldwide, around 90% of tanneries use this product [9]. Thus, environmental contamination from industrial effluents has aggravated the situation of environmental degradation, and among toxic metals, the one that is most prominent is chromium [10]. Furthermore, high concentrations of chromium can have direct impacts on physiological and biochemical factors of vital functions in the human body [11]. Thus, it is necessary to evaluate the possibility of using vegetable tannins to replace chromium salts in the tanning of animal skins.

Given the assumptions, the aimed was to evaluate the physicomechanical resistance, histological and morphological aspects in different directions of the fibers of the paiche *Arapaima gigas* (Schinz, 1822) to evaluate the tanning with chromium oxide and vegetable tannin.

## Material and methods

The study was conducted by the Universidade Federal de Rondônia (UNIR) and the tanning of the skins was carried out in March 2021, at the Tannery Textures da Amazônia, from Ji-Paraná, RO—Brazil. The research met the requirements of the Ethics Committee on the Use of Animals, UNIR (CEUA), of the Department of Veterinary Medicine at UNIR, under registration number 012/2021.

For the tanning and coloring process, 42 ½ skins with an average *in natura* weight of 1.0 ± 0.40 kg of 21 fish of 12 ± 0.5 kg were used, distributed in a completely randomized design and in a 2 x 3 factorial. Tanning followed the pattern described by Hoinacki [12] and Souza [13], with some modifications, it is emphasized that the steps are described (Table 1). The skins were processed at different times, that is, a drum was used for tanning with vegetable tannin and then with chromium salts. After 12 days of tanning the skins, a time of 16 hours was stipulated to measure the moisture of the skins. For this purpose, the Drying and control systems device was used. At the end of the tanning process, the skins were stabilized at 18.0% moisture, measuring the area, weight and thickness of the skin with a caliper for further analysis.

**Table 1:** Percentages of products, time corresponding to each step, process observations, pH in each step of the Nile tilapia (*O*. *niloticus*) skin tanning process.

Percentages (%)	Products	Time	Note
**Re-sauce**			
200.0	H_2_O	20’	Exhaust/wash
0.4	Surfactant MK IV^®^		
**Gutter**			
100.0	H_2_O	120’	Exhaust
0.5	Surfactant MK IV^®^		
2.0	Lime		
8.0	Sodium Sulfide		
**Underliming**			
100.0	H_2_O	20’	Exhaust
0.5	Surfactant MK IV^®^		
2.0	Ammonia Sulfide		
2.0	Dekalon^®^		
**Purge**			
100.0	H_2_O		
0.5	Surfactant MK IV^®^		
2.0	Ammonia Sulfide	20’	Exhaust/wash
100.0	H_2_O		
0.4	Proteolytic Enzyme	40’	
0.4	Surfactant MK IV^®^		
**Underliming**			
100.0	H_2_O		
2.0	Dekalon^®^	-	
2.0	Ammonia Sulfide	20’	
0.5	Surfactant MK IV^®^	20’	Exhaust/wash
**Píkel**			
100.0	H_2_O		
*	Sodium Chloride	15’	8^th^ Bet
3.0	Formic Acid	60’	
**Tanning**			
5.0	Chrome Salts (Kromium PP^®^)	60’	Even pikel bath
15.0	vegetable tannin (Weibull^®^)		
**Basification**			
1.5	Sodium bicarbonate	To dilute and 3x15’/60’	Same tanning bath
**Neutralization**			
1.5	Sodium bicarbonate	60’	
**Re-tanning and Dyeing**			
100.0	H_2_O		
2.0	Weibull^®^	30’	
2.0	Syntac F^®^		
1.0	Tamol LBA^®^		
2.0	Dye**	60’	Exhaust
**To grease**			
8.0	Superdema GW and AF 2.0% formic acid	60’	Emulsion H_2_O to 60° C
		30’	Exhaust

* = Sufficient sodium chloride to obtain 8^th^ Bet.

### Commercial and composition data

Two tanning agentes, commercially named salts chromium (6% Chromosal B^®^ chromium) and vegetable tannin—*Acacia mearnsii* De Wild (Fabaceae) and two dyes and annatto *Bixa orellana* L. (Bixaceae) were evaluated, with seven repetitions for each treatment. The skins, frozen (-18.0° C), were thawed and grouped into lots with an fish average of 14.0 kg.

The chemical tanning process was basically with skins were subjected to the steps of pickling, soaking, liming (8% more dermascal and 3% hydrated lime) (twice), peeling, purging, degreasing, pickling, tanning (five treatments), neutralization, retanning (2% of Relugan RV^®^ synthetic tannin + 1% Tamol LBM^®^ synthetic tannin), dyeing, polishing, drying, softening and finishing.

Clarotan-x8^®^ vegetable tannin is a sulphited mimosa extract, Syntac F^®^ is a phenolic-based replacement tannin and Syntac CW^®^ is chemically modified tannin, that is, derived from naphthalene sulfonic acids and vegetable tannins. Relugan RV^®^ is an anionic polymeric retanning agent, Weibull^®^ is a vegetable tannin based on mimosa extract and Tamol LBM^®^ is a naphthalene synthetic tannin.

Also according to the tanning process, paiche (*A*. *gigas*) skin when tanning with chromium salts and tannin vegetable, they go through several basification processes, immersions in different pH series and also through tanning periods, to finally obtain the leather for the fish industry, these two methodologies are meticulously elaborated in the studies by Hoinacki [12] and Souza [13].

### Physicomechanical tests

**Fig 1:**
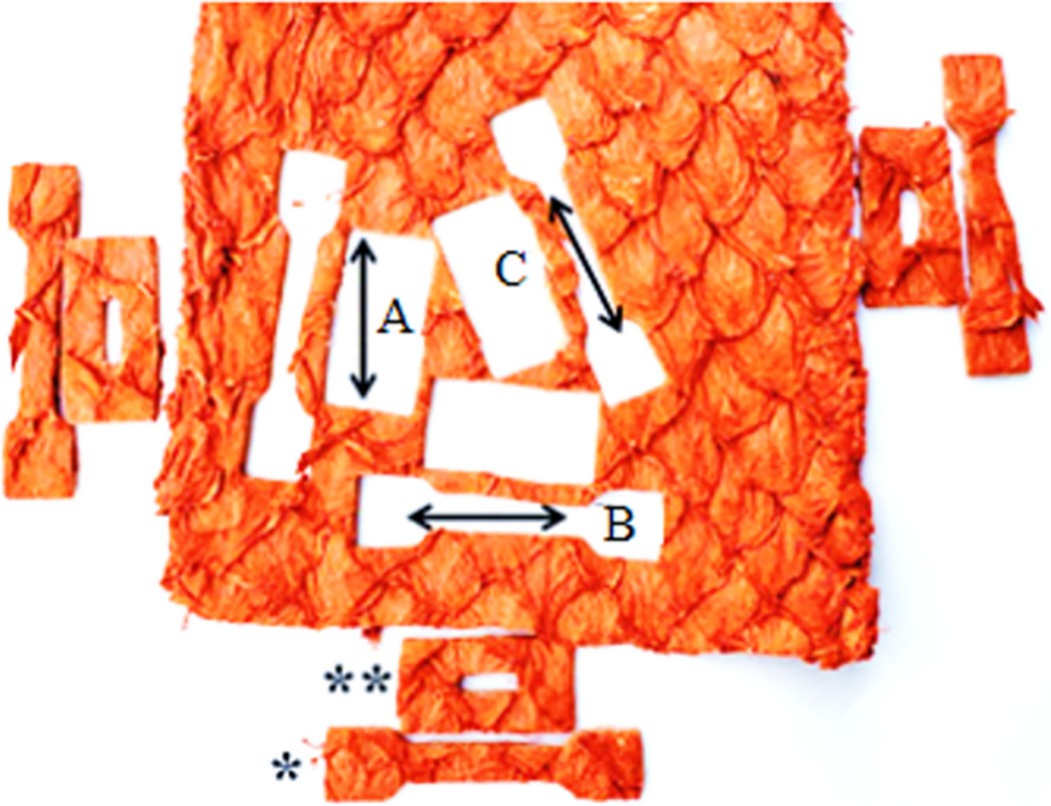
Paiche (*A*. *gigas*) leather with the removal of the specimens used for the tests to determine traction and elongation (*) and progressive tearing (**). Specimens taken longitudinally (A), transversal (B) and diagonal (C) to the length of the fish body.

After tanning, the dry skins were sent to the Skin and Leather Processing Laboratory, Universidade Estadual de Maringá (UEM), located at the Experimental Farm of Iguatemi, from Iguatemi, PR—Brazil. The skins were kept in an air-conditioned environment (23.0° C and 50.0% relative humidity) for 24 hours, according to ABNT [14]. The specimens were removed by treatment (tanning with chromium salts and vegetable tannin) with the aid of a rocker, according to ABNT [15], and with a caliper, the thickness of each specimen was measured at two points, according to ABNT [16]. The specimens were removed in the dorsal region of the paiche and tanned by the two tanning agents, longitudinally, transversally and diagonally to the length of the fish’s body (Fig 1).

Tensile strength, elongation strength was determined according to ABNT [17] and progressive tear strength according to ABNT [18]. For the strength determination tests, the force (N) applied in the test, the tension (N/mm^2^) until the breakage of the leather were evaluated, and for the evaluation of the elasticity of these leathers the elongation test (%) and for the progressive tearing (N/mm). For the resistance tests, an EMIC brand dynamometer was used, with a separation speed between loads of 100 ± 20 mm/mm. A 200 kgf load cell was used and calibration was performed by Emic-dcame, a calibration laboratory accredited by CGCRE/INMETRO under n° 197.

### Histological analysis and scanning electron microscopy

Samples were collected from each direction of the paiche (*A*. *gigas*) leather tanned with vegetable tannin and embedded in paraffin to perform the cuts for histological analysis. The samples were cut approximately 5.0 μm thick and stained using the hematoxylin-eosin (HE) technique [19] to describe the histology of the dermis. Histological sections were analyzed by light microscopy and photographed in a Calrs Zeiss/AxioLab and AxioxKop Zeiss photomicroscope.

For scanning electron microscopy, samples in different leather directions in relation to the length of the fish were taken to analyze the distribution of collagen fibers in the dermis and insertion coverslips and scale protection. Small samples were taken in different directions (longitudinal, transversal and diagonal), on the surface of the leather flower and on the face of the flesh. The samples were simply fixed with adhesive tape on the stabs for analysis in the SHIMADZU-SS550 scanning electron microscope, provided by the Central Research Support Complex (COMCAP/UEM). The samples were not metallized, as a low vacuum was used.

### Physicochemical analysis

The preparation of leather samples for chemical analysis followed the conditions required by ABNT standards [14], for the determination of chromium oxide Cr_2_O_3_, according to ABNT [20], determination of extractable substances with dichloromethane (CH_2_C_l2_) according to ABNT [21] and the determination of the pH and the pH differential figure of an aqueous extract, according to ABNT [22].

### Experimental design and statistical analysis

The experimental design was completely randomized in a 2 x 3 factorial scheme, with two tanning agents (chromium salts and vegetable tannin) and three evaluation directions (longitudinal, transversal and diagonal) and the results of the analyzed variables were presented as average ± standard deviation for each treatment tested. ANOVA was used followed by multiple comparisons test, the Tukey’s test (α = 0.05). For all analyses, the SAS (TASSEL) Inst program was used. Inc., Bradbury, NC, USA [23]. For the results of histology, scanning electron microscopy and physical-chemical analysis, the characterization of the skin was performed.

## Results

**Fig 2:**
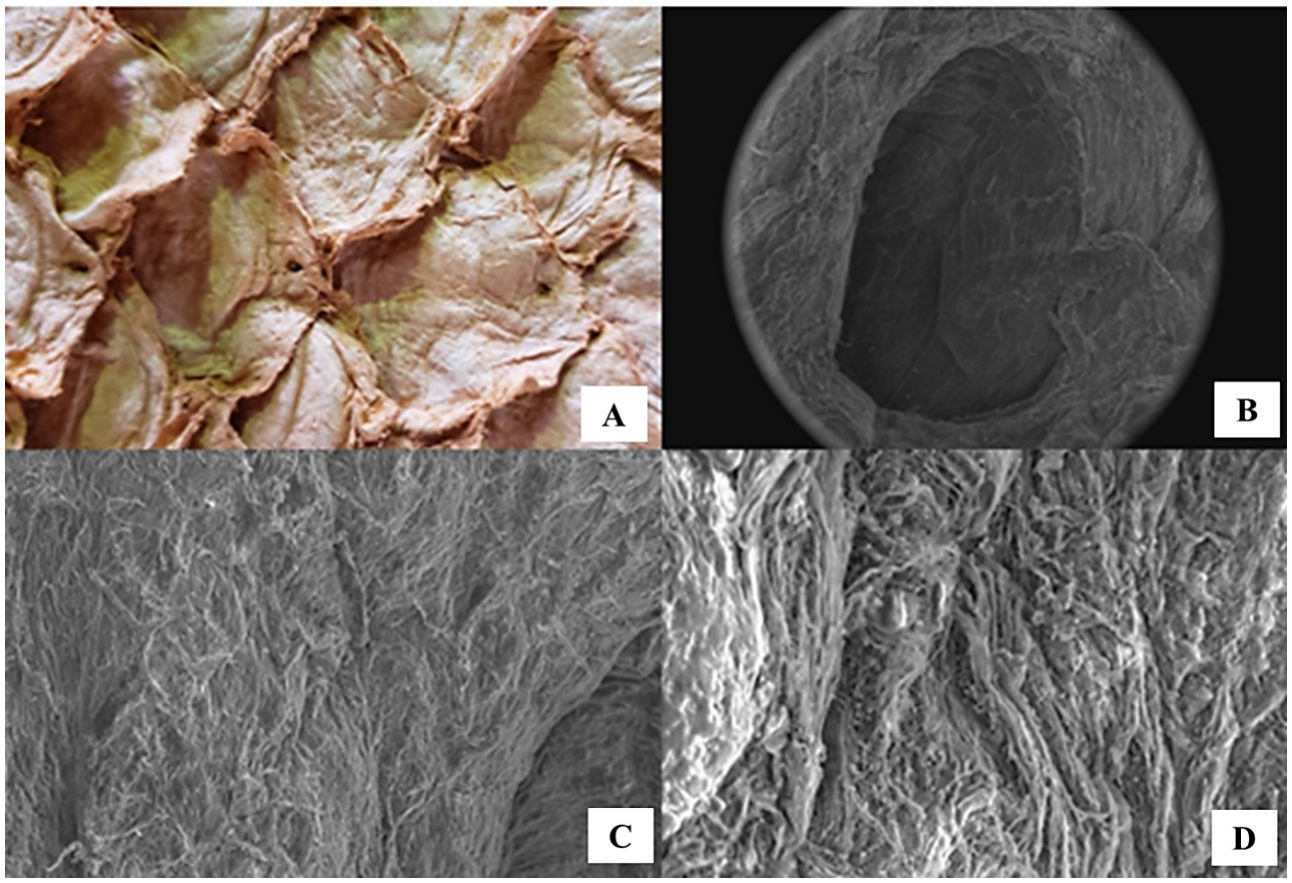
(A) Paiche (*A*. *gigas*) leather surface showing the protection coverslips and scale insertion (junction point between three coverslips), (B) Scanning Electron Microscopy of the lateral line orifice, and around (C and D) showing the fine collagen fibers without the epidermal layer.

After tanning, skins of the paiche (*A*. *gigas*) presented greater thickness in the animal’s dorsal region and thinner in the ventral region. Through the analysis, a reduction in the thickness of the dorsal region (2.9–3.8mm) for the ventral region (2.0–2.9mm) is noted. In the central region of the leather, the average thickness ranged 1.8 to 2.8mm. The protective coverslips and the insertion of the scales are long and deep and thick (Fig 2A). The variation in thickness of these coverslips ranges from 0.45 on the sides and 1.0mm in the center, which corresponds to the junction point between the three coverslips.

The average thickness of the specimens of the paiche (*A*. *gigas*) leather ranged 1.79 to 2.82mm (Tables 2 and 3). When analyzing the thickness of the specimens used for the tensile and elongation tests, there was an interaction (P<0.05) for the tanning agent and the sense of removal of these specimens. With the unfolding of this interaction, it can be inferred that the leathers with vegetable tannin were thicker (P<0.05) than those tanned with chromium salts, that is, the vegetable tannin provided a leather with 24.59% thicker compared to tanned with chromium salts. Therefore, the vegetable tannin provided a greater thickness to the leather, making the leather more full-bodied.

**Table 2:** Traction and elongation of the paiche (*A*. *gigas*) leathers tanned with vegetable tannin and chromium salts in longitudinal, transversal and diagonal directions.

Leather		Thickness (mm)	Force (N)	Tractive (N/mm^2^)	Deformation (mm)	Stretching (%)
**Tannin**	Longitudinal	2.76 ± 0.42^a^^1^	154.71 ± 97.96^a^	4.48 ± 0.84^a^	33.00 ± 14.51^a^	55,00 ± 24,81^a^
	Transverse	2.20 ± 0.34^ab^	108.28 ± 31.36^a^	4.82 ± 1.24^a^	35.29 ± 4.39^a^	59,00 ± 7,04^a^
	Diagonal	2.36 ± 0.25^ab^	160.43 ± 61.47^a^	6.65 ± 2.13^a^	29.29 ± 3.99^a^	48,57 ± 6,77^a^
**Chrome**	Longitudinal	1.77 ± 0.26^c^	95.71 ± 37.95^a^	4.71 ± 1.72^a^	35.71 ± 11.10^a^	59,57 ± 18,49^a^
	Transverse	1.82 ± 0.27^c^	125.29 ± 59.83^a^	8.49 ± 2.71^a^	37.86 ± 9.89^a^	62,57 ± 15,86^a^
	Diagonal	1.94 ± 0.37^b^	109.29 ± 71.34^a^	8.61 ± 1.53^a^	26.00 ± 6.40^a^	43,29 ± 10,42^a^
**Main effects**						
Tanner	Tannin	2.44 ± 0.40^a^	141.14 ± 69.87^a^	5.32 ± 1.73^b^	32.52 ± 8.95^a^	54,19 ± 15,26^a^
	Chrome	1.84 ± 0.20^a^	110.00 ± 67.43^a^	7.28 ± 3.24^a^	33.19 ± 10.35^a^	55,14 ± 16,91^a^
Direction	Longitudinal	2.26 ± 0.62^a^	125.21 ± 56.45^a^	4.68 ± 1.30^b^	34.36 ± 12.53^ab^	57,28 ± 21,16^ab^
	Transverse	2.01 ± 0.35^a^	116.79 ± 46.73^a^	6.66 ± 2.78^a^	36.57 ± 7.47^a^	60,79 ± 11,94^a^
	Diagonal	2.16 ± 0.35^a^	139.85 ± 69.26^a^	7.64 ± 2.05^a^	27.64 ± 5.40^b^	45,93 ± 8,88^b^
P-value						
Tanning (T)		0.0001	0.1240	0.0012	0.8160	0.8418
Direction (D)		0.1183	0.7569	0.0003	0.0388	0.0381
T x D		0.0244	0.2370	0.0533	0.6210	0,6492
CV^2^ (%)		14.7100	50.8400	34.6333	28.0500	28.0867

If there are averages followed by different letters (a, b, c) in the columns, they are different by Tukey’s test (p<0.05);

^1^Averages ± standard deviation followed in the same column by Tukey’s test (α = 0.05);

^2^CV = Coefficient of Variation.

**Table 3:** Determination of progressive tearing of the paiche (*A*. *gigas*) leathers tanned with vegetable tannin and chromium salts in longitudinal, transversal and diagonal directions.

Leather		Thickness (mm)	Tear (N/mm)	Maximum Force (N)	Average Force (N)
**Tannin**	Longitudinal	2.82 ± 0.63^a^	44.99 ± 14.36^a^	125.57 ± 44.12^a^	101,00 ± 3579^a^
	Transverse	2.76 ± 0.42^a^	31.14 ± 8.29^a^	83.42 ± 25.96^a^	60,14 ± 18,34^a^
	Diagonal	2.81 ± 0.37^a^	43.60 ± 10.98^a^	121.57 ± 26.19^a^	95,92 ± 25,62^a^
**Chrome**	Longitudinal	2.15 ± 0.59^a^	39.41 ± 8.94^a^	84.12 ± 22.15^a^	66,37 ± 20,67^a^
	Transverse	1.85 ± 0.44^a^	37.69 ± 9.70^a^	70.28 ± 23.54^a^	53,85 ± 17,76^a^
	Diagonal	2.16 ± 0.43^a^	43.40 ± 9.99^a^	91.85 ± 19.95^a^	71,14 ± 11,85^a^
**Main effects**					
Tanner	Tannin	2.80 ± 0.46a^1^	39.92 ± 12.63^a^	110.19 ± 37.03^a^^1^	85,52 ± 32,03^a^
	Chrome	2.06 ± 0.49^b^	40.14 ± 9.37^a^	82.18 ± 22.71^b^	63,91 ± 18,06^b^
Direction	Longitudinal	2.46 ± 0.68^a^	42.02 ± 11.69^a^	103.47 ± 39.22^a^	82,53 ± 32,90^a^
	Transverse	2.31 ± 0.63^a^	34.42 ± 9.32^a^	76.86 ± 24.77^b^	57,00 ± 17,65^b^
	Diagonal	2.49 ± 0.51^a^	43.51 ± 10.09^a^	106.71 ± 27.17^a^	83,29 ± 22,95^a^
P-value					
Tannin (T)		0,0001	0.9370	0.0022	0.0036
Direction (D)		0,5550	0.0600	0.0116	0.0043
T x D		0,7569	0.3117	0.4040	0.2567
CV^2^ (%)		20,4444	26.2900	29.2000	30.7373

If there are averages followed by different letters (a, b, c) in the columns, they are different by Tukey’s test (p<0.05);

^1^Averages ± standard deviation followed in the same column by Tukey’s test (α = 0.05);

^2^CV = Coefficient of Variation.

Concerning the direction of removal of the specimens, the longitudinal and transversal directions presented significantly less thickness when tanned with chromium salts, while the diagonal direction did not differ only from the longitudinal when tanned with vegetable tannin.

Concerning the removal direction of the specimens, only the diagonal direction of the leathers tanned with chromium salts did not differ (P>0.05) of the transversal and diagonal direction of the leathers tanned with vegetable tannin. The force applied in the tensile and elongation test did not differ (P>0.05) for tanning agents and leather direction (Table 2), whose values ranged 110 to 141N for the rupture of the specimen to occur.

The leather elasticity (%) and deformation (mm) were not influenced (P>0.05) by the use of different tanning agents, but by the directions of removal of the specimens. The leathers in the transverse direction (60.79%) showed greater elasticity compared to the diagonal direction (45.93%), while the longitudinal (57.78%) did not differ from the directions (P>0.05). As for the deformation, there was an increase in the length of the specimen after the determination of the leather elasticity of 3.7 cm for the transverse direction, 3.4 cm for the longitudinal and 2.7 cm for the diagonal (Table 2).

For the determination of traction there was a difference (P>0.05) for the tanning agents and sense of removal of the specimens (P<0.05). Leathers tanned with chromium salts had greater strength (7.28 N/mm^2^) compared to tannin (5.32 N/mm^2^). This reflected 26.92% more resistance when chromium salts were used as a tanning agent. When analyzing the direction of the leather, the longitudinal showed lower tensile strength (4.66 N/mm^2^) in relation to the others, that is, the specimen was 14.71% less resistant in relation to the transverse direction and 63.25% to diagonal.

After analyzing the progressive tearing of the paiche (*A*. *gigas*) leathers, the specimens of leathers tanned with vegetable tannin required 25.42% more maximum tear strength compared to those tanned with chromium salts. The tear determination does not depend on the tanning agents (P>0.05), as these did not influence the leather strength (Table 3). However, the maximum and average applied strength showed difference (P<0.05), that is, the leathers tanned with vegetable tannin needed greater strength to finish tearing the specimens. However, in the direction of the leather, the specimens removed in the transverse direction required less tear strength (P<0.05), as they had a smaller amount of reticulin fibers (Fig 2A).

After the tanning process, the flower design of the paiche (*A*. *gigas)* leather can be seen (Fig 2A). It is observed that above each coverslip, at the junction point between the three inserts and scales protection coverslips (Fig 2B), there is an orifice corresponding to the lateral line that runs along the hide along the length of the fish body. Around the orifice there are bundles of fine collagen fibers arranged horizontally to the dermis and others intertwining between them (Fig 2C and 2D).

**Fig 3:**
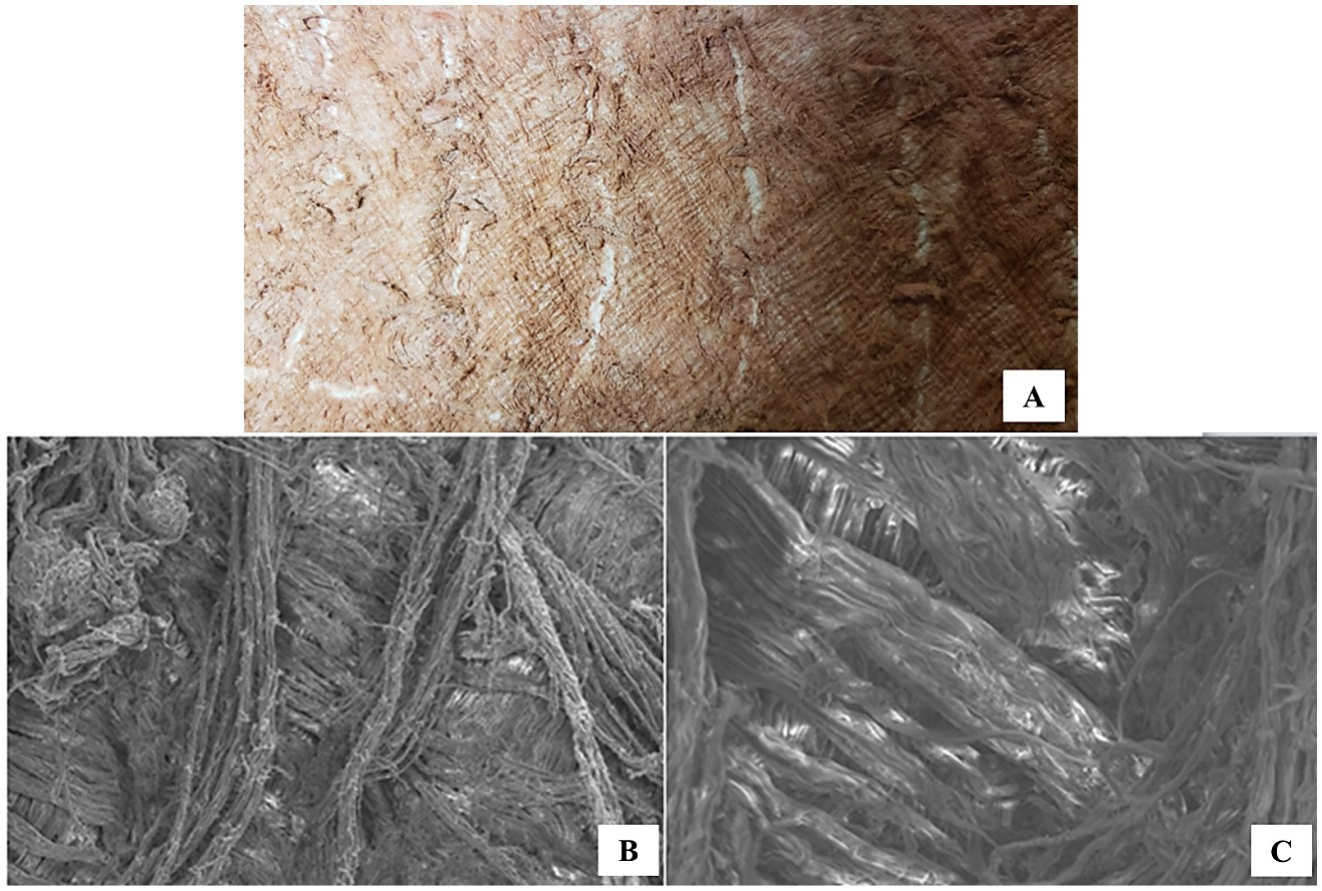
(A) Paiche (*A*. *gigas*) leather after tanning, showing the arrangement of collagen fiber bundles observed on the flesh side of the leather, (B and C) Scanning Electron Microscopy showing the arrangement and orientation of the collagen fiber bundles of the fleshy side of the paiche leather in overlapping layers.

**Fig 4:**
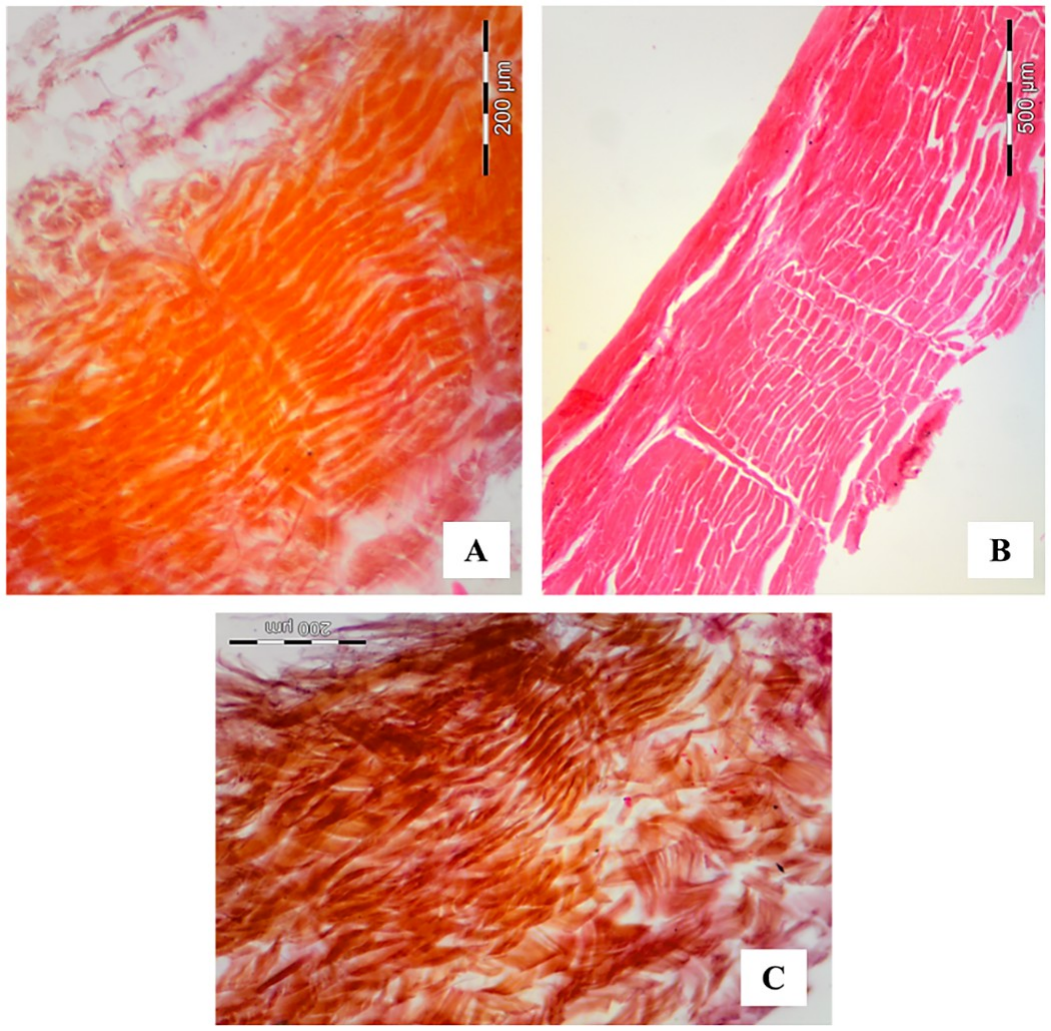
Photomicrographs of the paiche (*A*. *gigas*) leather cuts in different directions in relation to the length of the fish’s body, slides in HE staining. (A) longitudinal direction, (B) transverse direction and (C) diagonal direction.

**Fig 5:**
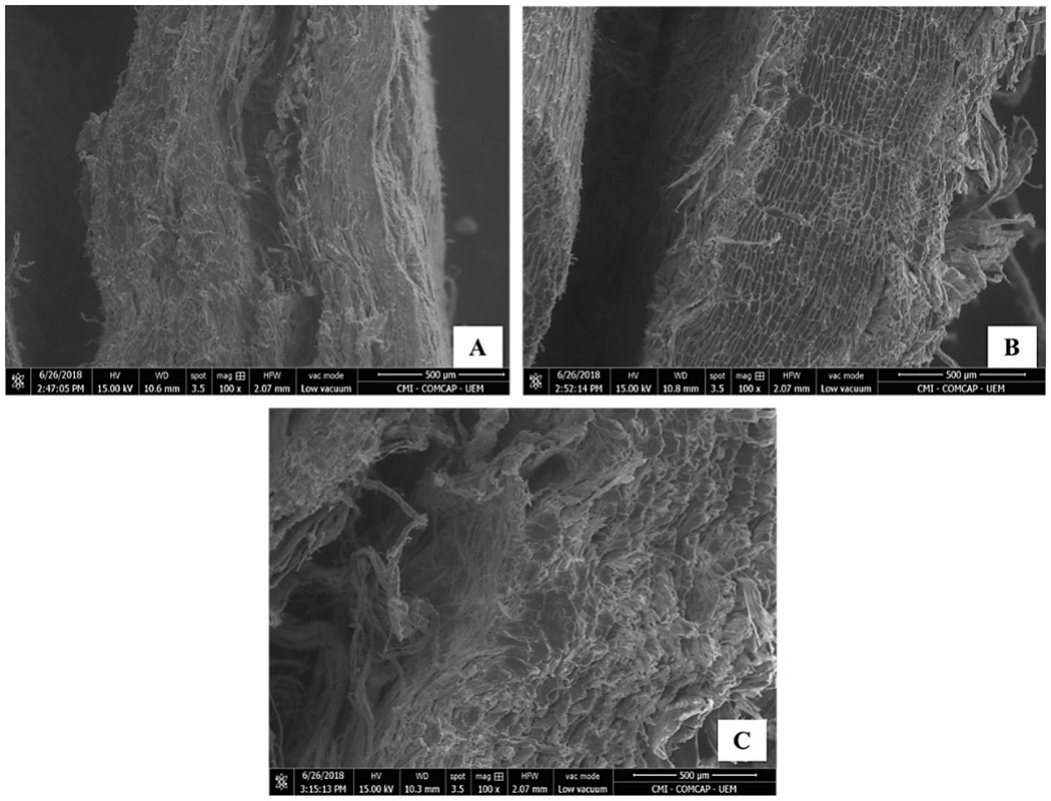
Scanning Electron Microscopy of the paiche (*A*. *gigas*) leather cuts in different directions in relation to the length of the fish’s body. (A) longitudinal direction, (B) transverse direction and (C) diagonal direction.

Paiche (*A*. *gigas*) leather presents superimposed layers of collagen fiber bundles interspersed and intertwined with each other. Fig 3A shows the flesh side of the leather, after tanning, and Fig 3B and 3C shows the scanning electron microscopy of this same location to better clearly observe this organization of the intertwining of collagen fiber bundles. Collagen fibers appear in layers parallel to the surface of the paiche (*A*. *gigas*) leather in the longitudinal cut images (Fig 4A). Collagen fibers are thinner near this surface and increase in thickness as they move to the side of the flesh (hypodermis). In the place where the scale should have been inserted (Fig 5A), in the upper part, there is a differentiated dermal tissue that corresponds to the protective coverslip, in the insertion of the scale. The fabric of this cover slip is made up of much finer fibers and overlapping joints in relation to the rest of the leather (Fig 2B–2D).

In the images of the cut of the paiche (*A*. *gigas*) leather in the transverse direction (Figs 4B and 5B) it is observed that the collagen fibers are found in layers parallel to the leather surface and space-in-space transverse fibers cross the parallel ones, providing a more binding firm on the leather. It is possible to observe the distribution of the collagen fibers of the coverslip thinner and together, and as they move away from the surface, they increase in thickness and spacing between them (Figs 4B and 5B).

Figs 4(C) and 5(C) show the images with the distribution of collagen fiber bundles of the paiche (*A*. *gigas*) leather taken diagonally to the length of the animal’s body. On the diagonal, it is noted that the intertwining is more intense, that is, in addition to the thinner intercalated superimposed layers, close to the surface, fiber bundles can also be seen crossing each other, resembling a network (Fig 4A).

Analyzing the images of scanning electron microscopy and histology, where the intertwining architecture, arrangement and orientation of collagen fibers are observed. As well as the superimposed layers of these fibers, confirm the physicomechanical results obtained of the tanned leathers in the longitudinal, transversal and diagonal directions (Table 3).

The leather in the transverse and diagonal direction presented greater traction (N/mm^2^) for the rupture of the specimen, that is, the collagen fibers of the leather. Observing the images (Figs 4B, 4C, 5B and 5C) the way the fibers intertwine diagonally and transversally provide greater tensile strength as the fibers are distributed parallel to the surface, perpendicular and crossed between them. On the fleshy side of the leather, it is possible to visualize this form of orientation and distribution of collagen fibers (Fig 3B and 3C).

Results of the percentages of chromium oxide, substances extractable with dichloromethane, as well as the pH and differential figure, for the paiche (*A*. *gigas*) hides tanned with chromium salts and vegetable tannin (Table 4). In this way, amount of chromium oxide in leather is related to the proportion of tanning agent fixed to collagen fibers. In this treatment, 5% of chromium salts were added in the tanning step and another 5% in the retanning step, while in the treatment with vegetable tanning, it was 15% in the tanning and the same percentage in the retanning, with no chromium oxide being found in the samples. In addition, the final pH of the tanned leathers was 3.43 for tannin tanning and 3.38 for chromium salts. In this pH range there is better fixation of dye and oils used in the fatliquoring step (Table 4).

**Table 4:** Chromium oxide, extractable substances with dichloromethane, pH and differential figure for the paiche (*A*. *gigas*) leathers tanned with chromium salts and vegetable tannin.

Tanning agent	Chromium Oxide Cr_2_O_3_ (%)	pH	Differential Cipher ***	Extractable Substances with Dichloromethane (%)
Chrome salts	0.27	3.38	0.83	16.57
Vegetable tannin	0.00	3.43	0.80	21.97
Standard/Method	IT10.4–009	IT10.4–008	IT10.4–008	IT10.4–007

***Differential figure only acts as a criterion for the presence of strong free acids or bases in aqueous extract with pH values below 4.0 or above 10.

It is important to highlight that in this experiment the leathers retanning with vegetable tannin presented a better pH value, closer to the recommended one, therefore, a better condition for fixing the vegetable tanning agent and the oils used in the process and the higher the differential sifra.

Accordingly, as the pH value was slightly below the recommended value, it is noted that the differential value presented values of 0.8 and 0.83, respectively, for leathers tanned with vegetable tannin and chromium salts, the maximum recommended being 0.7. This presented that there was formation of strong free acids in the analyzed samples. For the treatment with chromium salts, the pH was lower, more acidic, the result of the differential was also higher (0.83), because at the time of fixation or addition of the formic acid used, it was above what should be added, providing greater amount of free acid between the leather’s collagen fibers.

Due to the tanning process, the pH of the leather is usually acidic, and for tanning with chromium salts the acidity should be around 3.0 and for tannin 4.0 in the pickle stage; at the end of the process for fixing dyes, a tanning agent, especially vegetable tannin and oils, should be around 3.5. When the pH is below this value, it averages that an excessive amount of acid is found inside the leather, which can cause it to weaken over time. Excessive acidity causes the degradation of the protein chain, through acid hydrolysis, reducing the leather’s resistance.

It is interesting that the technique used provides greater resistance to the leathers (tensile, stretching and progressive tear), with minimal application of chromium salts or absence of these salts. Comparing the results reported with those obtained in this experiment with the paiche (*A*. *gigas*) leather, it can be inferred that in addition to the types of tanning agents applied in the tanning process, the species is essential in the evaluation of strength, as it presents an architecture in the arrangement and orientation of collagen fibers that determines a greater or lesser resistance to the leather after processing.

## Discussion

### Physicomechanical tests

Similar results were obtained in the resistance of the skin of the Bullfrog (*Rana catesbeiana*), tanned with chromium salts and vegetable tannin. The vegetable tannin provided thicker leathers (longitudinal = 0.74mm and transversal = 0.81mm) than those tanned with chromium salts (longitudinal = 0.74mm and transversal = 0.71mm). However, it was only significant for leather in the transverse direction (tannin = 0.81mm and chromium = 0.71mm) [24].

The tanning technique, the type and percentage of the tanning agent and the stage in which they were used (tanning or retanning) influence the results of leather resistance [25, 26]. It is also interesting to compare the resistance of the paiche (*A*. *gigas*) hides with the hides of other fish species and even other animals to check the quality of this innovative leather that is paiche hide for its application in clothing, among others.

Some studies evaluated the effects of different tanning agents on rabbit skins, the treatment using chromium provided greater elasticity to the leather [25, 27]. Leathers of the *Piaractus mesopotamicus*, with thickness ranging 0.73 to 0.88 mm, obtained traction values for the longitudinal and transverse directions of 5.93 N/mm^2^ and 13.81 N/mm^2^ for the progressive tearing of 15.66 N/mm and 13.85 N/mm and for the elongation of 52.20 and 76.98%, respectively [28]. In this paiche’s (*A*. *gigas*) study, traction values were lower than those found by Souza et al. [28]. However, for the tear, paiche leathers presented the highest average values, therefore, more resistant. The elasticity of paiche leathers was very close to the values of the *P*. *mesopotamicus* leather [27].

The skin of the *Colossoma macropomum* subjected to tanning with vegetable tannin and chromium salts was analyzed, reported mean values of 13.86 N/mm^2^ and 8.39 N/mm^2^ for the tensile test, 55.64 and 34.64% for elongation and 34.85 and 22.64 N/mm for progressive tearing, respectively [29]. Comparing the results obtained with the paiche (*A*. *gigas*) leather with the aforementioned authors, the elasticity of the *C*. *macropomum* leather was lower, and easier to tear (Table 3). However, comparing the resistance of the leathers as a function of the tanning agent, it is noted that the *C*. *macropomum* leather, when tanned with vegetable tannin, presented greater resistance than the paiche (*A*. *gigas*) leather. However, in addition to the tanning agent, the leather species must be evaluated due to the histological architecture of collagen fibers, concentration of products used, especially the amount or percentage of tannin used per kilograms of fresh skin. However, when tanned with chromium salts, the resistance of paiche (*A*. *gigas*) leather was very close to that obtained for the *C*. *macropomum*.

### Morphology, histology and scanning electron microscopy

Resistance of Nile tilapia (*O*. *niloticus*) leather is primarily due to the histological structure of the skin, that is, the architecture of the collagen fiber bundles in the deep dermis [30]. In this case, an arrangement of layers of collagen fibers is formed, superimposed parallel to the surface of the skin and interspersed perpendicularly to it, forming a tie between these collagen fibers, which consequently allows greater resistance to the tanned leather. Generally in fish, the dermis consists of a relative layer of diffuse tissue, a zone called the compact stratum [31]. This is rich in collagen fibers, which are arranged parallel to the surface of the skin and crisscrossed in sheets or layers, in the form of criss-cross networks as in mammals.

Average longitudinal elongation of the collagen fibers for the Cobia (*Rachycentron canadum*) was evaluated, which obtained 71.45% for leather treated with chromium salts and 81.82% when treated with vegetable tannin [32]. Some researchers have reported that the dermis presents a structural arrangement of collagen fibers, allowing the skin to have great resistance to different traction forces [31, 33]. For this reason, the skin of some species of fish can be used commercially in the manufacture of leather artifacts.

Additionally, when fish leather has a greater amount of reticulin fibers binding the collagen fibers, this leather has greater tear resistance [13]. Therefore, everything indicates that in the longitudinal and diagonal directions they present more reticulin fibers tying to the fiber bundles. Other researchers have also stated that the amount of thick layers of collagen fibers has an effect on the mechanical properties of the skin, thus providing greater resistance to the leather when pulled [34].

### Physical-chemistry tests

The physical-chemical tests of the paiche hides indicate that the tanning agent chromium salts (Table 4) provided lower chromium oxide content in the hides (0.27%), lower than what Hoinacki [12] indicates as a minimum value 3.0% chromium oxide or above 2.5%, BASF recommendation [35] for leathers tanned with chromium salts.

Final pH of the tanned leathers was 3.43 for tannin tanning and 3.38 for chromium salts. However, the best pH according to Hoinacki [12] should be a minimum value of 3.5. The recommended maximum is 0.7 [12]. There may be a reduction in the physical-mechanical resistance of the leather when the amount of acid is high (low pH), as it has a corrosive power on collagen fibers [36, 37]. This acidity of the leather can cause the oxidation of metallic components, if placed in contact with the leather, such as rivets, buckles and eyelets, as well as causing allergy or irritation for the user.

For Substances Extractable with Dichloromethane, the percentage was 21.97% for leather with vegetable tannin and 16.57% for leather with chromium salts, that is, the amount of oil fixed in the paiche (*A*. *gigas*) leather. The oils added in the tanning process act in the sliding of collagen fibers causing them to slide over each other as it is subjected to some type of traction, providing greater mobility or elasticity to the leather. Substances extractable in dichloromethane must be at most between 16 to 18% for the leather to be used in clothing [35]. Thus, only leathers tanned with chromium salts could be used for making clothing.

Some parameters to industrial standard are proposed in the scientific literature. Depending on the purpose of using the leather, characteristics must be tested for paiche leather. The color fixation of organic dyes, for example, depends on the previous tanning agent and the stress to which it is subjected. It is observed greater color fixation to paiche leather tanned with organic tannin under natural dye submitted to water and/or light compared to the worst results when submitted to heat stress. It is suggested to evaluate contraction temperatures of tanned leather with the proposed tanning agents, given the relevance of this aspect in the manufacturing process of shoes and other accessories in which high temperatures are used.

## Conclusions

Leathers tanned with vegetable tannin are less resistant than leathers tanned with chromium salts for traction to rupture. Tanning agents do not interfere with the elasticity and deformation of the paiche (*A*. *gigas*) leathers, except when evaluated separately where the transverse direction of the leather presents greater elasticity and deformation.

The quality characteristics of leathers have varied with new market niches due to the countless purposes to which they apply. In the past, when resistance and durability were prioritized, today new market niches expand in terms of flexibility, visual characteristics, representativeness of the rustic and the use of organic products. Therefore, paiche leather has important physical-mechanical characteristics compared to traditional bovine leather and has its added value when subjected to organic products and processes that maintain the product’s natural characteristics, ensuring color fixation and/or collagen fiber characteristics such as use of organic tanning and natural dyes. Ecological leather from Amazon paiche can add value to products and add market value to leather accessories and artifacts with an appeal of sustainable origin.

## Supporting information

S1 File (PDF)
