# Supplementary material for: Tanned leather of the paiche Arapaima gigas Schinz, 1822 (Arapaimidae) with extracts of vegetable origin to replace chromium salts
Source: PLoS One. 2022 Jan 21;17(1):e0261781. doi: 10.1371/journal.pone.0261781 (PMC8782319; doi:10.1371/journal.pone.0261781)
Supplement: S1 File — (PDF) [file pone.0261781.s001.pdf]

FUNDAÇÃO UNIVERSIDADE FEDERAL DE RONDÔNIA
PROGRAMA DE PÓS-GRADUAÇÃO EM CIÊNCIAS AMBIENTAIS

Patrícia Silva de Oliveira Kanarski

EXTRATOS DE ORIGEM VEGETAL EM SUBSTITUIÇÃO AOS PRODUTOS QUÍMICOS NO CURTIMENTO E TINGIMENTO DA PELE DO PIRARUCU NA AMAZÔNIA

Rolim de Moura, RO
2018

Patrícia Silva de Oliveira Kanarski

EXTRATOS DE ORIGEM VEGETAL EM SUBSTITUIÇÃO AOS PRODUTOS QUÍMICOS NO CURTIMENTO E TINGIMENTO DA PELE DO PIRARUCU NA AMAZÔNIA

Dissertação apresentada ao Programa de Pós-Graduação em Ciências Ambientais, como requisito parcial para obtenção do título de Mestre em Ciências Ambientais, sob a orientação da Drª. Jucilene Cavali e coorientação da Drª Maria Luiza Rodrigues de Souza.

Rolim de Moura, RO

PATRICIA SILVA DE OLIVEIRA KANARSKI

Extratos de origem vegetal em substituição aos produtos químicos no curtimento e tingimento da pele do pirarucu na Amazônia

Dissertação apresentada ao Programa de Pós-Graduação em Ciências Ambientais, como requisito parcial para obtenção do título de Mestre em Ciências Ambientais sob a orientação da Drª Jucilene Cavali e coorientação da Drª Maria Luiza Rodrigues de Souza.

APROVADA: 30 de julho de 2018

Profª Drª JUCILENE CAVALI
Universidade Federal de Rondônia (Orientadora)

Profª Drª MARIA LUIZA RODRIGUES DE SOUZA
Universidade Estadual de Maringá (Coorientadora)

Prof. Dr. MARLOS OLIVEIRA PORTO
Universidade Federal de Rondônia (Avaliador Interno)

Drª ANA PAULA SARTORIO CHAMBO
Universidade Estadual de Maringá (Avaliador Externo)

Prof. Dr. ELVINO FERREIRA
Universidade Federal de Mato Grosso (Avaliador Interno)

Ficha catalográfica elaborada por

K161e Kanarski, Patrícia Silva de Oliveira.

Extratos de origem vegetal em substituição aos produtos químicos no curtimento e tingimento da pele do pirarucu na Amazônia / Patrícia Silva de Oliveira Kanarski. -- Rolim de Moura, RO, 2018.

73 f. : il.

Orientador(a): Prof.ª Dra. Jucilene Cavali

Coorientador(a): Prof.ª Dra. Maria Luiza Rodrigues de Souza.

Dissertação (Mestrado Acadêmico em Ciências Ambientais) – Fundação Universidade de Rondônia

1.Tanino Vegetal. 2. Arapaima Gigas. 3. Corante . 4.Curtente. 5.Urucum. I. Cavali, Jucilene. II. Título.

CDU 615.451.1

Dedico este trabalho aos meus pais (*in memorian*), ao meu filho Guilherme, e ao meu esposo Adailton, à minha orientadora prof.ª Drª. Jucilene Cavali e a minha Coorientadora Maria Luiza Rodrigues de Souza.

AGRADECIMENTOS

Ao programa de Pós-graduação em Ciências Ambientais (PGCA).

À FAPERO pela bolsa concedida.

À MK Química Do Brasil por se dispor a fazer as análises físico-mecânica.

À Leather Solutions por realizar as análises de Colorimetria.

À profª. Jucilene Cavali pelo carinho, paciência e orientação durante o desenvolvimento deste trabalho.

À profª Maria Luiza de Souza, pela dedicação e cuidado.

Ao graduando do curso de Zootecnia, Gúliti Wandami dos Santos, pelas contribuições neste trabalho.

Aos meus pais que mesmo não estando mais presente, sei que olham e cuidam de mim.

Ao meu companheiro, parceiro, marido, amigo, Adailton. Por ser sempre carinhoso e esforçado em me ajudar.

Ao meu filho Guilherme, por ser tão carinhoso e compressivo.

Ao Sr. Antônio, que selecionou os peixes de forma rigorosa no seu tanque em contribuição à pesquisa.

À minha equipe Texturas da Amazônia pela colaboração.

E a todos que de alguma forma contribuíram para execução e realização deste trabalho. A todos o meu MUITO OBRIGADA!

LISTA DE TABELAS

Tabela 1 - Principais características físico-mecânicas, métodos de ensaios e limites segundo as normas UNI para uma confecção de calçados 23

Tabela 2 - Parâmetros utilizados na análise química de acordo com a ABNT para couros em wet-blue 23

Tabela 3 - Principais características físico-mecânicas e químicos, métodos de ensaios de couros para vestuário segundo as normas ABNT NBR BRASILEIRA 13525 (2016) 24

Capítulo I

Tabela 1 - Determinação da tração, alongamento e rasgamento progressivo do couro de *Arapaima Gigas* curtidos em diferentes níveis de tanino vegetal 34

Capítulo II

Tabela 1 - Tração e alongamento dos couros de Pirarucu curtidos com tanino vegetal e sais de cromo nos sentidos longitudinal, transversal e diagonal 50

Tabela 2 - Determinação do rasgamento progressivo dos couros de Pirarucu curtidos com tanino vegetal e sais de cromo nos sentidos longitudinal, transversal e diagonal 51

Tabela 3 - Valores médios dos testes físico-mecânicos dos couros de Pirarucu curtidos com tanino vegetal e sais de cromo..59

Capítulo III

Tabela 1 - Valores médios da avaliação físico-mecânica dos couros de Pirarucu curtidos com tanino vegetal e sais de cromo e submetidos ao tingimento com corante vegetal (urucum) e químico...75

Tabela 2 - Rasgamento progressivo dos couros de Pirarucu curtidos com tanino vegetal e sais de cromo e submetidos ao tingimento com corante vegetal (urucum) e químico 78

Tabela 3 - Teste físico-mecânico dos couros de Pirarucu curtidos com tanino vegetal e sais de cromo e submetidos ao tingimento com corante vegetal (urucum) ou químico 80

Tabela 4 - Análise colorimétrica em couros de pirarucu curtidos com tanino vegetal e sais de cromo submetidos ao tingimento com corante químico e vegetal com base no sistema...81

Tabela 5 - Dados médios da solidez da cor do couro à luz UV, à água e ao suor, do couro de Pirarucu curtido com sais de cromo ou tanino vegetal e corado com urucum ou corante químico...82

SUMÁRIO

RESUMO 11

ABSTRACT 13

1 INTRODUÇÃO GERAL 14

2 REVISÃO DE LITERATURA 18

3 TECNOLOGIA DE PROCESSAMENTO DE PELES 20

4. IMPACTO ECONÔMICO E AMBIENTAL 25

REFERÊNCIAS 26

CAPITULO I 27

1 DIFERENTES CONCENTRAÇÕES DE TANINO VEGETAL NO CURTIMENTO DO COURO DO PIRARUCU 27

1.1 Introdução 31

1.2 Objetivo Geral 32

1.3 Material e Métodos 32

1.4 Resultados e discussão 33

1.5 Conclusão 34

1.6 Referências 35

CAPITULO II 37

2 CARACTERISTICAS DO COURO DO PIRARUCU CURTIDO COM EXTRATOS DE ORIGEM VEGETAL EM SUBSTITUIÇÃO AOS SAIS DE CROMO 37

2.1 Introdução 41

2.2 Objetivo Geral 42

2.3 Material e Métodos 42

2. 4 Análise Histológica do Couro de Pirarucu 45

2.5 Análise de Microscopia Eletronica de Varredura 46

2.6 Composição Centesimal da Pele e dos Couros de Pirarucu 46

2.7 Resultados e Discussões 47

2.8 Análise físico-mecânica dos couros de pirarucu curtidos com tanino vegetal e Sais de Cromo no Sentido Longitudinal, Transversal e Diagonal 47

CONCLUSÃO..60

REFERÊNCIAS 61

CAPITULO III 63

3 TANINO VEGETAL E EXTRATO DE URUCUM EM SUBSTITUIÇÃO AOS PRODUTOS QUÍMICOS NO CURTIMENTO E TINGIMENTO DO COURO DO PIRARUCU 63

3.1 Introdução 67

3.2 Objetivo Geral 69

3.3 Objetivos Específicos 69

3.4 Material e Métodos 70

3.5 Resultados e discussões 74

CONCLUSÕES..84

REFERÊNCIAS 85

CONSIDERAÇÕES FINAIS...86

RESUMO

O Brasil é um dos maiores exportadores mundiais de couro, sendo a pele bovina responsável por mais de 60% da matéria prima para a produção do couro *wet blue* (couro inacabado). Rondônia o maior produtor de pirarucu em cativeiro, espécie em potencial para produção do couro ecológico da Amazônia. O couro com escamas representa de 10 a 20 % do peso corporal, é exótico e inovador com especificidades de resistência e grande aceitação em vários segmentos de mercado. O uso de curtentes vegetais em substituição ao cromo apresenta-se como alternativa a produção ecológica e agregação de valores na cadeia do pescado e aproveitamento do couro na Amazônia. Objetivou-se, com a pesquisa, identificar a melhor concentração de tanino vegetal no curtimento da pele do pirarucu; e, comparar tanino vegetal com sais de cromo, identificar alternativas sustentáveis para a produção do couro, substituindo corantes químicos por naturais. Desenvolvida pela Universidade Federal do Rondônia, no Curtume Texturas da Amazônia, utilizou-se 61 peles de pirarucu de 13 ± 0,8 kg de peso corporal, oriundos de viveiros escavados e certificados. No ensaio 1 objetivou-se identificar a melhor concentração de tanino vegetal no curtimento da pele do *Arapaima gigas*. Utilizou-se 20 peles *in natura* de 1,1 ± 0,39 kg de pirarucus de 13,2 ± 1,4 kg de peso corporal distribuídas em delineamento inteiramente casualizado em 5%, 10%, 15% e 20% do agente curtente tanino vegetal e cinco repetições. Os níveis de tanino influenciaram na força de tração e rasgamento progressivo do couro de pirarucu ($P<0,01$). O aumento das concentrações de tanino vegetal reduz a resistência á tração de ruptura em 3,16 N/mm^2 e aumenta o rasgamento progressivo do couro em 2,08 N/mm por unidade percentual do curtente. O curtente não influenciou na elasticidade e o alongamento médio dos couros curtidos foi de 83,87%. A concentração de 15% de tanino vegetal é recomendada ao processo de curtimento da pele do Pirarucu pois apresenta maior estabilidade físico-mecânica do couro para confecção de vestuários e artefatos. No Ensaio II objetivou-se comparar os curtentes tanino vegetal e sais de cromo e corantes químico e natural. Utilizou-se delineamento casualizado em fatorial 2 x 2 onde a combinação tanino vegetal e urucum preservou a integridade das fibras colágenas com maior espessura do couro (2,10mm vs. 2.61mm) e demandando mais força (178,2N vs. 125,3N), para danos físico-mecânicos. As médias para a força de tensão á tração foi de 6,38N/mm^2, rasgamento 30,3mm, alongamento 50,6% e rasgamento de 40,0N/mm ($P>0,05$). A concentração de 15% de tanino vegetal no curtimento não influenciou a resistência á tração ($P<0,05$) no sentido do couro, 15,99 N/mm^2; enquanto as demais concentrações apresentaram menor resistência no sentido transversal ao corpo do animal. Ao se comparar curtentes (sais de cromo e tanino vegetal) com

corantes (urucum e químico) em fatorial 2 x 3 com 7 repetições a espessura média da derme do Pirarucu observou-se que o tanino vegetal proporciona couro 24% mais espesso; 34,78% mais resistente à aplicação de força; 3,21% mais resistente a tensão à tração e 10,4% mais elástico comparado ao couro curtido com sais de cromo. O uso de corante e curtente naturais são eficazes na substituição aos curtentes e corantes químicos no processo de produção do couro ecológico do Pirarucu.

Palavras-chave: *Arapaima gigas*. curtente orgânico. couro de peixe. gigante da Amazônia. urucum

ABSTRACT

Brazil is one of the world's largest exporters of leather, being the bovine skin responsible for more than 60% of the raw material for the production of leather *wet blue* Pirarucu leather represents 10 to 20% of body weight, is exotic and innovative with resistance specificities and great acceptance in various market segments. Rondônia has an annual production of 7,9 tons of Pirarucu. The use of extracts or regional natural waste is presented as an alternative to ecological production and aggregation of values in the chain of fish and hides in the Amazon. The objective of this study was to identify the best concentration of vegetable tannin in the tanning of the Pirarucu; to compare vegetable tannin with chromium salts, identify sustainable alternatives for leather production, replacing chemical dyes with natural ones. Developed by the Federal University of Rondônia, in Tannery Textures of Amazon, 61 Pirarucu skins of 13 ± 0.8 kg of body weight were used, from nurseries excavated and certified, distributed in a randomized design in factorial 2 x 2, where the tannin and Urucum combination preserved the integrity of the thicker collagen fibers (2.10 mm vs. 2.61 mm) demanding more force (178.2N vs. 125.3N), for physical-mechanical damages. The averages for tensile force the traction was 6.38 N / mm², tearing 30.3mm, stretching 50.6% and tearing 40.0N / mm (P> 0.05). The concentration of 15% of vegetable tannin in the tanning did not influence the tensile strength (P <0.05) in the direction of leather, 15.99 N / mm^2; while the other concentrations showed less resistance transverse to the body of the animal. When comparing tanning agents (salts of chromium and vegetable tannin) with dyes (Urucum and chemical) in 2 x 3 factorial with 7 replicates, the average thickness of the Pirarucu dermis was observed that the vegetal tannin provides leather 24% thicker; 34.78% more resistant to the application of force; 3.21% more resistant to tensile stress and 10.4% more elastic compared to leather tanned with chrome salts. The use of natural dye and tanning agents are effective in replacing the tanning and chemical dyes in the process of producing the ecological leather of Pirarucu. The physical-mechanical, physical-chemical analyzes were performed at MK QUÍMICA DO BRASIL, and the colorimetric analyzes were Leather Solutions Laboratory, in Rio Grande do Sul, respectively.

Key words: Vegetable tannin. *Arapaima Gigas*. Urucum. Dye.tanning. Leather.

1 INTRODUÇÃO GERAL

Segundo Santos (2002) a indústria do couro, no Brasil, é composta por aproximadamente 450 curtumes, sendo em sua maioria, cerca de 80%, de pequeno porte e administração familiar. Estima-se que do total de couro produzido, 66% é oriundo de bovinos (CICB, 2002).

De modo geral, couro é uma pele animal beneficiada por inúmeros processos para a confecção de vestuários, utensílios e apliques estéticos na indústria automobilística.

A indústria do couro participa de várias cadeias produtivas, sendo seus principais vínculos, a pecuária e a indústria frigorífica que fornecem sua matéria prima, pele. Esse setor é composto especialmente por curtumes, que fornecem seus produtos a diferentes indústrias para fabricação de diversos insumos: calçados e artefatos, vestuário, móveis e automobilística. A heterogeneidade é uma das principais características dessa indústria, onde seu produto final irá depender do tipo de couro fornecido pelos frigoríficos/curtumes, podendo produzir e fornecer couros em diferentes estágios de acabamento: o couro salgado, mais simples (*wet blue)* e o couro semiacabado (*crust)*; sendo aquele; produto do processo inicial de salgamento do couro, para conservação e transporte, o que lhe agrega pouco valor; e este, sendo fruto do processo de secagem do couro, tornando-o produto semiacabado destinado ao processo de acabamento. O couro pode ser obtido de diversos tipos de animais, como equinos, caprinos, bovinos e peixes, onde o couro bovino é predominante neste mercado (ABDI, 2011).

O Brasil detém o maior rebanho comercializável do mundo, ressaltando que o rebanho indiano é maior em número absoluto, mas por questões religiosas há restrição à comercialização; colocando o Brasil na ponta e com vantagem competitiva em relação à Índia (SENAI, 2002). Detentor do segundo maior rebanho bovino do mundo, atrás somente da Índia, o Brasil é o maior exportador de carne no mercado internacional, onde a região norte do país é a segunda maior em população de bovinos. Rondônia, localizado nessa região, é o 6º maior rebanho do Brasil e segundo maior rebanho da região (IBGE, 2016). Este estado é, atualmente, o maior produtor de peixes em cativeiros de água doce do Brasil, produzindo, em 2016, mais de 90 mil toneladas desse produto (IBGE, 2016). Esses números demostram o potencial do estado de Rondônia, mas também demonstra a quantidade de resíduos sólidos produzidos e sem destinação correta para seu reaproveitamento.

A indústria do couro, no Brasil, teve início no século XIX, mais precisamente em 1824, com a chegada dos primeiros imigrantes alemães ao sul do país. Atividades predominantes da época, como agricultura e a pecuária, motivaram os imigrantes a implementarem a indústria do

artesanato, focados na confecção de arreios para montaria, atividades estas que constituíam as primeiras atividades industriais da época. A exportação de couros e calçados iniciou-se após o fim da primeira grande guerra, onde sua maior expansão foi logo após o fim da segunda guerra, onde a demanda de mercado estava localizada na América latina, fornecendo coturnos para os exércitos brasileiros e venezuelanos (CORREA, 2001).

A década de 90 foi um período de grande crescimento para a indústria brasileira de couro, com um crescimento de 40%, passando de 23,5 milhões de couros em 1991, para 33 milhões em 2001, representando 10% do mercado mundial na época. Grande parte dessa produção está concentrada nas regiões sul e sudeste que, somadas, ocupam mais 72% da produção nacional (SANTOS, 2002).

O setor coureiro nacional despertou para os problemas e as dificuldades, buscando soluções tecnológicas e parcerias. A integração de fatias do setor começa a se tornar mais comum. Na Austrália e EUA, mais de 70% dos curtumes pertencem a grupos que possuem frigoríficos (KOZEN, 2006). Integrar frigorífico e curtume possibilitam ganhos financeiros dos diversos tipos, entre eles tributários, logísticos e controle de qualidade (FRIZZO FILHO, 2002).

O mercado brasileiro movimentou 33,5 milhões de couros em 2000, sendo 30,5 milhões em exportação e 3 milhões em importação, onde esta cresceu mais rapidamente, atingindo uma taxa de 4,8% ao ano durante a década (KOZEN, 2006).

Com participação econômica relevante, em diversos países, a indústria do couro movimenta a ordem de US$ 70 bilhões (ABER et al., 2010). Como líder mundial na exportação de couro, o Brasil processa anualmente cerca de 42 milhões de couros, onde essa produção tem relevância no PIB brasileiro. AZEVEDO (2002) afirma que aproximadamente 74% das peles produzidas no Brasil são exportadas direta ou indiretamente, configurando um dos setores industriais mais abertos ao comércio exterior no país. No Brasil essas estruturas coureiras se concentram mais na região sul do país, onde o estado do Rio Grande do Sul possui o maior numero de instalações (FIGUEIREDO et al., 2010).

Os principais concorrentes internacionais do Brasil, na indústria do couro, são: Coréia do Sul, Itália e China. No entanto, a qualidade do couro produzido no Brasil é inferior. Segundo a Embrapa Gado de Corte – EMBRAPA apenas 8,56% do couro produzido pelos curtumes nacionais são de qualidade superior, ao contrário dos EUA, que possuem essa qualidade em 85% de sua produção (KOZEN, 2006)

Caracterizado como um dos maiores rebanhos bovinos do mundo, em 2016 o Brasil ultrapassou a casa das 218 milhões de cabeças (IBGE, 2016), ocupando, segundo a FAO (2008), a quarta posição em produção de couro, onde a líder de mercado, a China, participa com mais

de 29% da produção mundial. Desta forma observa-se uma característica da indústria brasileira de couro no comercio internacional, com a exportação de grande parte de sua produção (ABDI, 2011).

Segundo Santos (2002) a indústria do couro, no Brasil, é composta por aproximadamente 450 curtumes, sendo em sua maioria, cerca de 80%, de pequeno porte e administração familiar. Estima-se que do total de couro produzido, 66% é oriundo de bovinos (CICB, 2002).

De modo geral, couro é uma pele animal beneficiada por inúmeros processos para a confecção de vestuários, utensílios e apliques estéticos na indústria automobilística.

A indústria do couro participa de várias cadeias produtivas, sendo seus principais vínculos, a pecuária e a indústria frigorífica que fornecem sua matéria prima, pele. Esse setor é composto especialmente por curtumes, que fornecem seus produtos a diferentes indústrias para fabricação de diversos insumos: calçados e artefatos, vestuário, móveis e automobilística. A heterogeneidade é uma das principais características dessa indústria, onde seu produto final irá depender do tipo de couro fornecido pelos frigoríficos/curtumes, podendo produzir e fornecer couros em diferentes estágios de acabamento: o couro salgado, mais simples (*wet blue)* e o couro semiacabado (*crust)*; sendo aquele; produto do processo inicial de salgamento do couro, para conservação e transporte, o que lhe agrega pouco valor; e este, sendo fruto do processo de secagem do couro, tornando-o produto semiacabado destinado ao processo de acabamento. O couro pode ser obtido de diversos tipos de animais, como equinos, caprinos, bovinos e peixes, onde o couro bovino é predominante neste mercado (ABDI, 2011).

O Brasil detém o maior rebanho comercializável do mundo, ressaltando que o rebanho indiano é maior em número absoluto, mas por questões religiosas há restrição à comercialização; colocando o Brasil na ponta e com vantagem competitiva em relação à Índia (SENAI, 2002). Detentor do segundo maior rebanho bovino do mundo, atrás somente da Índia, o Brasil é o maior exportador de carne no mercado internacional, onde a região norte do país é a segunda maior em população de bovinos.

A cadeia produtiva de couro inicia-se na atividade de pecuária, em que os diferentes tipos de sistemas de produção e manejo, podem resultar em peles com qualidades distintas, aplicando dificuldades ao processamento de couro (AZEVEDO, 2002).

A criação dos rebanhos, controle de parasitas, formas corretas de identificação, transporte e confinamento dos animais, são fatores importantes e que influenciam na qualidade final das peles fornecidas. Após o abate é necessário cuidados para evitar a degradação das peles. Se entre o tempo de abate e o processamento das peles para o curtimento for curto, entre

6 e 12 horas, a depender da temperatura, as peles podem ser armazenadas sem nenhum pré-tratamento, onde recebem a denominação de “peles verdes”. Porém, se as peles necessitam ser estocadas em períodos maiores de tempo ou transportadas por longas distâncias, principalmente em altas temperaturas, há a necessidade de pré-tratamento chamado “cura”, onde se empilha as mantas de pele, intercalando-as com camadas de sal, desta forma podendo ser armazenadas por meses (PACHECO, 2005)

2 REVISÃO DE LITERATURA

2.1 Arapaima Gigas e o Potencial de Produção do Couro

2.1.1 especificidades da pele de peixe

A pele apresenta muito mais do que pura e simples cobertura externa do animal. Ela responde continuamente a mudanças fisiológicas que ocorrem no corpo, refletindo muitas características tais como, condições geoclimáticas, tipo de alimentação, sexo, idade e estado de saúde (DANIELS, 2002; HOINACK et al, 1994).

2.1.2 histologia da pele

Priebe (2005) afirma que do ponto de vista de processamento para fins de obtenção de couro pele possui três camadas cutâneas, sendo a epiderme, derme e hipoderme.

A camada denominada epiderme e as escamas são eliminadas no princípio dos processos de ribeira, por meio de emprego de produtos químicos e enzimas, podendo muitas vezes ser substituídos por produtos naturais. No caso da fabricação do couro somente interessa a camada denominada derme. A epiderme corresponde a aproximadamente 1% da espessura total da pele, sua estrutura apresenta como um conjunto de células dispostas em camadas. Essa estrutura é completamente removida no decorrer das operações de depilação e caleiro. A derme contribui com 85% da espessura e é a camada de interesse na produção de couros. Os principais componentes da pele descritos por Hoinacki (1989) são as proteínas, graxas, outros compostos orgânicos e água. Dentre as proteínas o principal formador é o colágeno, esta estrutura é composta por três cadeias polipeptídicas em forma helicoidal, que consiste em aminoácidos unidos num enlace peptídicos. Os principais aminoácidos, são glicina, prolina e hidróxido-prolina.

O colágeno tem como principais características: grande resistência ao rasgamento, alta capacidade de absorção de água (esta, que pode ser facilmente libertada por algum tempo), insolubilidade em água e solventes orgânicos, absorção de água até 70% em peso do tecido (parcialmente depositada em forma de água hidratada ou água capilar) e preservação por desidratação (BASF, 2005).

A hipoderme é constituída por tecido adiposo, conectivo, vasos sangüíneos, nervos e músculos. Portanto, esses tecidos subcutâneos são eliminados mecanicamente juntamente com

restos de carne e gordura remanescentes a esfola do peixe. Esta camada é eliminada no princípio do processamento, nas operações de pré-descarne e/ou descarne.

2.1.3 regiões da pele

A pele não apresenta textura nem espessura uniformes em todas as suas regiões (BASF, 2005).

Como o objetivo de obter produtos de maior uniformidade, costuma-se dividir a pele em diferentes zonas, em relação às características relacionadas com textura fibrosa e a espessura (HOINACKI; GUTTHIEL, 1989). O mesmo autor afirma que a zona do grupão é a região mais rica em fibras colágenas e apresenta melhor entrelaçamento de fibras, já a região da zona dos flancos é mais pobre em material colágeno o que resulta em um menor entrelaçamento relacionadas com outras zonas. Essa diferença na estruturação pode estar interferindo na resistência dos couros. Pode-se observar muitas vezes dependendo da espécie uma diferença muito grande de espessura do couro na região dorsal e menor na ventral, correspondendo respectivamente ao grupão e flancos em mamíferos.

2.1.4 desenho da flor

Souza (2006) relata que a pele apresenta um desenho, que é uma característica própria de cada espécie animal. Após o curtimento, quando são removidas as escamas, é possível observar um desenho que constitui uma definição típica na pele, de acordo com a espécie animal. O desenho da flor é uma composição formada pelas lamélulas de proteção e inserção das escamas em peixe, enquanto para mamíferos seria referente a abertura dos folículos pilosos e poros na pele profundo se relacionados com papilas, também conhecido, como “flor da pele” (Figura 2) (HOINACKI; GUTTHIEL, 1989).

3 TECNOLOGIA DE PROCESSAMENTO DE PELES

3.1 Curtimento e Tingimento do Couro

Novas tecnologias no processamento e curtimento de peles exóticas têm sido desenvolvidas no Brasil, sendo a pele de peixes considerada como uma pele exótica e inovadora, em especial as peles de pirarucu, transformando-se em um couro inimitável decorrente da presença de suas lamélulas.

O objetivo de transformar a pele em couro é preservar as propriedades originais como resistência a tração, viscoelasticidade e abrasão. Com a finalidade de obtenção do processo apresenta uma série de etapas, nas quais as características químicas da pele são alteradas de acordo com a finalidade de cada produto final desejado (DURAISAMY, SHAMENA, BEREKETE, 2016).

De acordo com Hoinacki; Gutthiel (1989) e Souza (2008) com adaptações as fases de processamento dos couros se dividem em etapas com produtos químicos são de remolho, caleiro, desencalagem, purga, desengraxe, piquel, curtimento, neutralização, recurtimento, tingimento, engraxe e acabamento; e as etapas mecânicas de descarne, enxugamento, rebaixamento, estiramento, vácuo ou *toogling*, lixamento, amaciamento, prensagem e medição.

Dentro da cadeia de curtimento de couro é possível classificar os curtumes de acordo com a etapa do processo de curtimento (PACHECO, 2005):

a) curtume de **Wet Blue:**
   - desenvolve o primeiro processamento de couro, qual seja, logo após o abate, o couro salgado ou em sangue é despelado, graxas e gorduras são removidas e há o primeiro banho de cromo e o couro passa a exibir um tom azulado e molhado;

b) curtume **Integrado:**
   - realiza todas as operações, processando desde o couro cru até o couro acabado;

c) curtume de **Semi-Acabado**:
   - utiliza como matéria-prima o couro wet blue e o transforma em couro crust (semi-acabado);

d) curtume **de Acabamento:**
   - transforma o couro crust em couro acabado.

O processamento do couro consiste em 5 etapas: ribeira, curtimento, recurtimento, pré-acabamento e acabamento.

Figura 1 - Fluxograma para o Processo de Curtimento das Peles de Peixes

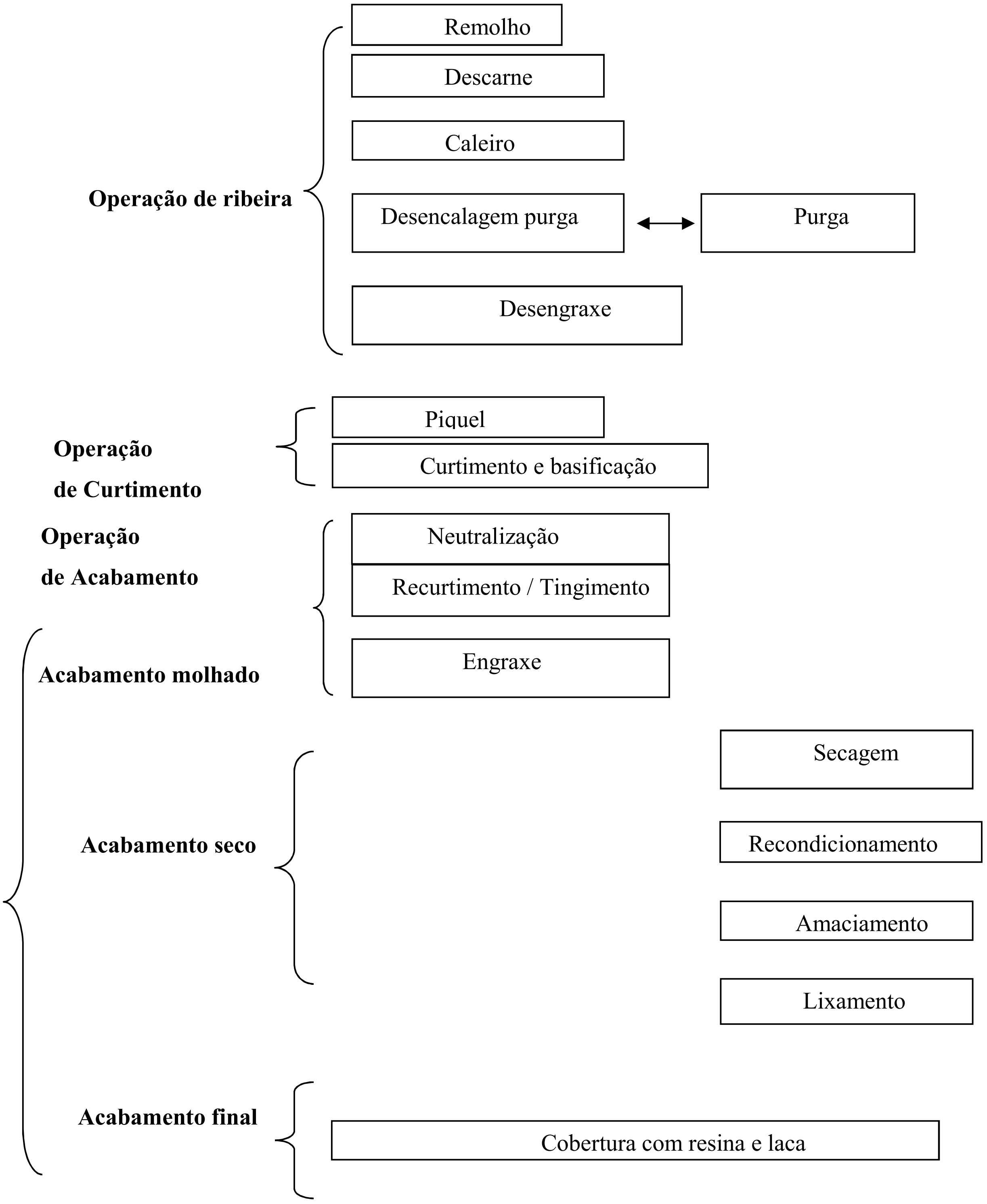

Fonte: Adaptado do Hoinacki; Gutthiel (1989) e Souza (2008).

A criação dos rebanhos, controle de parasitas, formas corretas de identificação, transporte e confinamento dos animais, são fatores importantes e que influenciam na qualidade final das peles fornecidas. Se entre o tempo de abate e o processamento das peles para o curtimento for curto, entre 6 e 12 horas, a depender da temperatura, as peles podem ser armazenadas sem nenhum pré-tratamento, onde recebem a denominação de "peles verdes". Porém, se as peles necessitam ser estocadas em períodos maiores de tempo ou transportadas por longas distâncias, principalmente em altas temperaturas, há a necessidade de pré-tratamento chamado "cura", onde se empilha as mantas de pele, intercalando-as com camadas de sal, desta forma podendo ser armazenadas por meses (PACHECO, 2005).

As etapas de difusão e fixação do curtente estão intimamente ligadas a basicidade do curtente. Quanto maior a basicidade maior a fixação nas camadas externas da pele e maior o poder curtente. Comumente usa-se curtentes de cromo com basicidade de 33% ou pH a baixo de 3, para garantir a penetração do cromo até atingir a basicidade. No entanto diminui-se a difusão para as camadas mais internas, podendo estas ser curtidas posteriormente ao aumentar a basicidade entre 45 a 50% através da adição de compostos alcalinos, como bicarbonato de sódio e o óxido de magnésio, a fim de permitir que os complexos de cromo possam penetrar na matriz colágena através da reação do complexo de cromo com proteína, na qual o cromo se liga diretamente ao grupo carboxílico do colágeno (FUCK; GUTIERRES; MARCILIO, 2007).

3.2 Controle de Qualidade

O controle de qualidade de couros prontos para comercialização semi acabado, em wet-blue ou wet-white, é importante. Para tanto, há necessidade de controle de sua qualidade para estipular preços de comercialização em função de suas características principalmente visuais. Mas, outros parâmetros são utilizados para avaliar a qualidade quantitativa, que refere-se a qualidade físico-mecânica dos couros a exemplo dos parâmetros e os limites para calçados de da UNI (Tabela 1).

Tabela 1 - Principais características físico-mecânicas, métodos de ensaios e limites segundo as normas UNI para uma confecção de calçados

Parâmetros	Método de análise	Especificações
pH do extrato aquoso	NBR 11057	Mínimo 3,5
Cifra diferencial (CD)	NBR 11057	Máximo 0,7
Teor de óxido de cromo (% Cr^2 O^3)	NBR 11054	Mínimo 3,5
Substância extraíveis em DCM (%DCM)	NBR 11030	Máximo 0,5
Material volátil (% MV)	NBR 11029	50 a 60
Cinza total sulfatada (% Cinzas)	NBR 11031	Máximo 12

Fonte: Sammarco, 2001.

Pribe (2005), também afirma que no que diz respeito ao *wet-blue*, seu principal objetivo é a verificação da qualidade do couro, tendo como parâmetro as normas internacionais e nacionais. Estabelecidos os padrões de qualidade para os couros brasileiros, o Comitê Brasileiro do Couro e Calçado da Associação Brasileira de Normas Técnicas (ABNT) elaborou a norma NBR 13525 – requisitos para aceitação de couro quanto à análise química, publicada, em 1995 apresentadas (Tabela 2).

Tabela 2 - Parâmetros utilizados na análise química de acordo com a ABNT para couros em wet-blue

**Características Físico-mecânicas**	**Método de Ensaio**	**Valores Requeridos**
Alongamento e ruptura da flor	UNI ISO 3379	> 7 mm
Carga de ruptura	ISO 3377	> 45 N
Resistência à tração	UNI ISO 3376	> 10 N
Resistência à flexão	UNI 8433	60.000 flexões
Permeabilidade ao vapor d’água	UNI 8429	> 1,5 mg/ cm ².h
Adesão do acabamento	ISO 11644	> 2,5 N/cm
Solidez	NBR 20105-2	Após 75 horas de exposição: nível 3 na escala de têxteis

Fonte: MK News, 2003.

Sendo assim, os controles quantitativos permitem avaliar as características dos couros e emitir certificados de qualidade (laudos). Não se pode esquecer dos controles empíricos que dão noções gerais sobre as características dos couros, nos quais, pode-se citar: toque da superfície do couro, flor solta, cobertura, fixação de corantes, adesão do acabamento, brilho,

dobra do couro, elasticidade da flor, defeitos, solidez à luz, penetração de água, uniformidade do tingimento, simulação do acabamento final (CTCCA, 2000). Isto tudo são informações da ABNT para couros bovinos, porém couros exóticos não são encontradas as referências, para tanto a única forma que se tem é comparar as características ou parâmetros dos couros de peixes ou exóticos aos de couro bovino para se ter uma ideia quanto a sua resistência, ou seja, sua qualidade.

Tabela 3 - Principais características físico-mecânicas e químicos, métodos de ensaios de couros para vestuário segundo as normas ABNT NBR BRASILEIRA 13525 (2016)

Parâmetros	Método de análise	Especificações
Tração	ABNT NBR ISO 3376	≥ 130 N
Alongamento	ABNT NBR ISO 3376	≥ 150 N<br>≥ 40%
Rasgamento progressivo	ABNT NBR ISO 3377-1	≥ 20 N
Teor de óxido de cromo III em couros (% $Cr^2 O^3$)	ABNT NBR ISO 5398-1<br>ABNT NBR ISO 5398-3<br>ABNT NBR ISO 5398-4	≥ 3,5 %
Determinação do teor de substâncias extraíveis em diclorometano (couros) % ED	ABNT NBR<br>11030 ISO 4048	≤ 0,8 %
Determinação de cifra Diferencial do couro	ABNT NBR<br>11057 ISO 4045	Cifra ≤ 0,7
Determinação do pH do couro	ABNT NBR<br>11057 ISO 4045	pH ≥ 3,5

Fonte: ABNT NBR 13525, 2016.

De acordo com Gutterres (2004) é importante avaliar a resistência do couro para aplicação em vestuários ou artefatos em geral, principalmente, utilizando tecnologias consideradas limpas nos sistemas de produção ambiental sustentável onde há buscas por processos industriais com melhor aproveitamento das matérias-primas e menos poluentes, tendo como ponto principal o uso de agente curtente a base de tanino vegetal.

4. IMPACTO ECONÔMICO E AMBIENTAL

Entre as diversas atividades econômicas que causam impactos ao meio ambiente destaca-se o setor pesqueiro. Esta atividade apresenta uma grande geração de resíduos em todas as etapas do seu processo produtivo, desde a captura até a comercialização do pescado. A piscicultura é uma atividade de considerável importância em todo o mundo, como fonte geradora de alimentos, emprego e renda para vários segmentos econômicos. Entretanto, um dos problemas relacionados a essa atividade, é a forma de destino final dos resíduos gerados, já que estes possuem uma alta carga de matéria orgânica, e se não forem gerenciados corretamente podem afetar tanto o solo, quanto os recursos hídricos

Com o advento da industrialização que a partir do séc. XVIII começou a incorporar os mais diversos tipos de recursos naturais, com o tempo também incorporou a atividade pesqueira que deixou de ser essencialmente artesanal e passou também a atender as demandas de mercado, assim intensificando a produção esta atividade passou a ser denominada de pesca industrial ou comercial (MELO, 1985).

O rejeito desses resíduos, seja de origem agrícola ou industrial, oriundos das mais diversas cadeias produtivas, cujos descartes indevidos podem causar impacto ambital e aumentar o gasto público. Esses resíduos são pele, vísceras, cabeça, espinha e escamas.

No Brasil, os resíduos da agroindústria, os urbanos industriais e os marinhos são descartados, em sua maioria, como lixo, desprezando seu potencial para transformação eu couro. Os resíduos marinhos seguem a mesma tendência, com destino para lixões ou despejados no mar.

Hoje já existe a necessidade do aproveitamento integral dos subprodutos gerados pelo cultivo de peixes é crescente, principalmente devido à porcentagem elevada dos resíduos após filetagem que tem sido um problema para o produtor ou para o abatedouro.

A pele pode ser beneficiada e resultar em uma matéria-prima de qualidade e de aspecto peculiar inimitável, após o curtimento, devido à sua resistência e desenho formado na sua superfície, principalmente as peles de peixes com escamas. Segundo Ingram e Dixon (1994), as peles de peixes são consideradas como um couro exótico e inovador, com aceitação geral em vários segmentos da confecção.

REFERÊNCIAS

ABER, S.; SALARI D.; PARSA, M.R. Employing the Taguchi method to obtain the optimum conditions of coagulation–flocculation process in tannery wastewater treatment. **Chemical Engineering Journal,** n.162, p. 127–134, 2010.

AZEVEDO, P. F. Competitividade da cadeia de couro e calçados. **Fórum de Competitividade da Cadeia Produtiva de Couro e Calçados.** Brasília: MDIC/PENSA, 2002.

BASF S.A. **Vade-mécum do Curtidor**. 5. ed. Ludwigshafen: BASF, 2005.

CORRÊA, A. R. **Panorama da indústria mundial de calçados, com ênfase na América Latina**. Rio de Janeiro: BNDES Setorial, 2001.

CARTILHA DO COURO. CTCCA - Centro tecnológico do couro, calçado e afins. p.18-20, 2000.

DURAISAMY, R., SHAMENA, S. BEREKETE A.K. A review of bio-tanning materials for processing of fish skin into leather. **International Journal of Engineering Trends and Technology.** v.39, n.1, p.10-20 September 2016.

FOOD AND AGRICULTURE ORGANIZATION - FAO. **Relatório de comité de problemas de produtos básicos**: Subgrupo sobre Cueros y Pieles, Séptima Reunion, Roma, jun., 2001.

FIGUEIREDO, J.A.S; PRODANOV, C.C.; DAROIT, D. Impacts of the globalized economy on the environment: the tanning industry in the Vale do Rio dos Sinos. **Brazilian Journal of Biology.** v 70, no. 4 (suppl.), p. 1231-1243. 2010.

FUCK, W. F.; GUTTERRES, M.; MARCILIO, N. R.. **Influência do acabamento molhado e do envelhecimento do couro na oxidação de cromo.** In: SEMINÁRIO DO PROGRAMA DE PÓS-GRADUAÇÃO EM ENGENHARIA QUÍMICA, 6. 2007: Porto Alegre. Anais... Porto Alegre, RS UFRGS/PPGEQ, 2007.

GUTTERRES, M. Estrutura de Colágeno na Pele. **Revista do Couro Abqtic**, Estância Velha, n. 170, 2004.

HOINACKI, E. GUTHIEL,N.C.. Peles e couros; origens, defeitos, industrialização. In: **Peles e couros; origens, defeitos, industrializaçao**. Porto Alegre: SENAI/RS, 1989. p.19.

INGRAM, P., DIXON, G. Fishskin leather: na innovate product. **Journal of the Society of Leather Technologists and chemists**, v.79, p.103-106, 1994

PACHECO, J. W. F. Curtumes. Série P+ L. **São Paulo: CETESB**, 2005.

SANTOS, A. M. M. M. et al. Panorama do setor de couro no Brasil. **BNDES Setorial, Rio de Janeiro**, n. 16, p. 57-84, set. 2002

CAPITULO I

1 DIFERENTES CONCENTRAÇÕES DE TANINO VEGETAL NO CURTIMENTO DO COURO DO PIRARUCU

RESUMO

O couro de peixe apresenta-se como alternativa a outros couros no uso em produtos, não somente por atributos estéticos, mas também por questões ambientais. Produtos desenvolvidos com esta matéria prima têm significativo valor agregado, dada à inovação e sustentabilidade. Dentre as diversas técnicas de curtimento, a mais utilizada mundialmente é com sais de cromo. Porém há necessidade de aplicação de produtos menos poluentes ao ambiente, sendo assim, são importantes estudos que avaliem a possibilidade do processamento das peles de animais sem a utilização de sais de cromo, sulfeto de sódio (agente basificante altamente tóxico) e querosene, utilizados comumente no processamento, desta maneira, produzindo um couro ecológico ou também denominado "bioleather". Alternativas para a produção deste tipo de couro, está na utilização de extratos vegetais e na operação de curtimento. A concentração de tanino é um fator relevante no processo de curtimento, tendo em vista, que a dosagem inadequada pode comprometer o acabamento final deste couro. Estudos comparando 10% de tanino vegetal e combinados com aos couros de tilápias tratados com tanino sintético proporcionaram maior resistência a tração (VIEIRA et al. 2008). Pensando nisso, o objetivo deste trabalho é verificar a concentração ideal de tanino vegetal para o curtimento de peles de pirarucu. O Estudo foi realizado pela Universidade Federal de Rondônia e o Curtimento das peles no Curtume Texturas da Amazônia, localizado em Ji-paraná, Rondônia, Brasil. A pesquisa atendeu aos requisitos no Comitê de ética ao Uso de Animais - CEUA número 031/2018. Foram utilizadas vinte peles retiradas de Pirarucu com peso corporal de 13,2 ± 1,4 kg provenientes de piscicultura licenciada para o cultivo da espécie, submetidas as concentrações de 5%, 10%, 15% e 20% do agente curtente tanino vegetal em cinco repetições ou peles inteiras com média de peso in natura de 1,1 ± 0,39/pele kg. O curtente utilizado foi o tanino vegetal marca TANAC S.A.© extraído da Acácia negra, *Acacia mearnsii*, do Rio Grande do Sul. Os testes físico-mecânicos foram realizados no laboratório de controle de qualidade da **MK QUÍMICA DO BRASIL**, RS em aparelho dinamômetro da marca EMIC®. A resistência físico-mecânica dos couros secos (23ºC e 50% de umidade relativa do ar por 24 horas, conforme ABNT - NBR ISSO 4044, 2015) foi

determinada a partir de 6 corpos-de-prova retirados da região dorsal de cada couro no sentido longitudinal em relação ao comprimento da pele do peixe com auxilio de balancim (ABNT - NBR, 11035, 2015). Através destes resultados pode-se inferir que o melhor nível de tanino vegetal a ser utilizado no curtimento do couro de pirarucu é de 15%, pois dependendo do teste realizado, a concentração proporciona uma maior dificuldade no deslizamento das fibras, fazendo com que reduza a elasticidade e diminuindo a resistência, em especial a de tração.

Palavras-chaves: couro de pirarucu. dosagens. tanino ecológico. *Arapaima Gigas*.

ABSTRACT

Fish leather presents itself as an alternative to other leathers in product use, not only for aesthetic attributes but also for environmental reasons. Products developed with this raw material have significant added value, given the innovation and sustainability. Among the various techniques of tanning, the most used worldwide is with salts of chromium. However, there is a need for the application of less polluting products to the environment and important studies are therefore being carried out to evaluate the possibility of processing animal skins without the use of chromium salts, sodium sulphide (highly toxic basifying agent) and kerosene commonly used in the processing, in this way, producing an ecological leather or also denominated "bioleather". Alternatives for the production of this type of leather, is in the use of vegetable extracts and in the tanning operation. The concentration of tannin is a relevant factor in the tanning process, considering that the inadequate dosage may compromise the final finishing of this leather. Studies comparing 10% of vegetable tannin and combined with the tilapia hides treated with synthetic tannin provided greater resistance to traction (VIEIRA et al., 2008). With this in mind, the objective of this work is to verify the ideal concentration of vegetable tannin for the tanning of pirarucu skins. The study was carried out by the Federal University of Rondônia and the tanning of the skins in Curtume Textures of Amazonia, located in Ji-paraná, Rondônia, Brazil. The research met the requirements in the Ethics Committee on the Use of Animals - CEUA number 031/2018. Twenty skins from Pirarucu were used, with a body weight of 13.2 ± 1.4 kg from fish farms licensed for the cultivation of the species, with concentrations of 5%, 10%, 15% and 20% of the tanning agent in five replicates or whole skins with average in natura weight of 1.1 ± 0.39 / skin kg. The tannin used was the vegetable tannin brand TANAC SA extracted from the black Acacia, Acacia mearnsii, from Rio Grande do Sul. The physical-mechanical tests were carried out in the quality control laboratory of MK QUÍMICA DO BRASIL, RS in a dynamometer of the brand EMIC®. The physical-mechanical resistance of dry leathers (23ºC and 50% relative humidity for 24 hours, according to ABNT-NBR ISO 4044, 2015) was determined from 6 specimens removed from the dorsal region of each leather in the longitudinal direction in relation to the skin length of the fish with the aid of rocker (ABNT - NBR, 11035, 2015). From these results it can be inferred that the best level of vegetable tannin to be used in the tanning of pirarucu leather is 15%, because depending on the test performed, the concentration gives a greater difficulty in the sliding of the fibers, making it reduce the elasticity and reducing the resistance, especially the tensile strength.

Key words: pirarucu leather. dosages. ecological tannin. *Arapaima Gigas.*

1.1 Introdução

O Brasil possui a maior reserva de agua do mundo, 8.000 km^3, produziu mais de 692 mil toneladas de peixes em 2017 e gerou 1 milhão de empregos (IBGE, 2017). O estado de Rondônia está no ranking da produção de peixes em cativeiro, com 90.560 toneladas em 14 mil hectares e 4.200 propriedades licenciadas (SEDAM, 2017).

O *Arapaima gigas* é a segunda espécie mais cultivada, 8% da produção comercial perfazendo 7.350 toneladas em 2016. O Estado é o maior produtor de Pirarucu em cativeiro e primeiro em tanques-lona, com autorização para cultivo comercial da espécie em cativeiro.

O couro com escamas de espécimes Pirarucu de cultivo de 12 a 14 kg de peso corporal corresponde a proporção média de 1,4 % e, levando-se em consideração o montante abatido o Estado de Rondônia possui potencial de produção de 560 mil peles para processamento e comercialização a confecções e acessórios (CAVALI et al, 2017).

O curtimento de pele de peixe com cromo orgânico é usual nos curtumes em função da facilidade e custo de aquisição do curtente e das características estáveis proporcionadas ás peles. Contudo, os taninos podem ser aplicados nas etapas de curtimento e recurtimento e, dependendo da finalidade do couro, serem dadas as características finais e diferentes ao couro, proporcionando maior maciez, elasticidade ou um couro mais espesso.

Os produtos mais utilizados para o curtimento são os sais de cromo, alumínio, zincônio e, dentre os taninos, os vegetais (extraídos de plantas) e os sintéticos. Os tanantes vegetais são misturas complexas de muitas substâncias encontradas em cascas, raízes, folhas e frutos. São extraídos do barbatimão (*Styphnodendron* barbatimão), angico (*Piptadenia rígida*), quebracho (*Schinopsis lorentzii*), mimosa (*Acacia decurens*) (SOUZA et al, 2008, KARDELL et al 2013), eucalipto (*Eucalyptus globulus*) (PINTO et al, 2013; VEIGA et al, 2013) e materiais biocurtentes como microrganismos (enzimas) considerados agendes do curtimento verde utilizado no curtimento de couro de peixes devido a sua biodegradação (DURAISAMY, SHAMENA, BEREKETE, 2016).

Os taninos vegetais possuem capacidade de precipitar alcaloides, gelatina e outras proteínas, interações estas que variam com a ação curtente, ou afinidade de um polifenol em se ligar com a estrutura fibrosa da proteína, da proporção tanino e proteína, do tamanho da cadeia molecular dos taninos condensados, de seu peso molecular e do número de hidroxilas fenólicas (HOINACKI, MOREIRA, KIEFER, 1994). São responsáveis por encorpar a espessura do couro, auxiliando na sua resistência ao rasgamento, flexibilidade e maciez, além de ser um produto ecologicamente correto, quando comparado ao cromo (CARDOSO, 2010, EIRAS et

al., 2015). Estudos comparando 10% de tanino vegetal e combinados com aos couros de tilápias tratados com tanino sintético proporcionaram maior resistência a tração (VIEIRA et al. 2008).

A pele dos peixes, por sua vez, possui diferentes respostas a concentração de tanino vegetal como curtente, atreladas a quantidade de colágeno da espécie, a idade, sexo, alimentação (SCHWARZ et al. 2018), destacando-se o *Arapaima gigas*, o "gigante da Amazônia" com grande proporção de tecido colagenoso.

1.2 Objetivo Geral

O objetivo deste trabalho foi avaliar a resistência físico-mecânica do couro do Pirarucu submetido ao curtimento com diferentes concentrações de tanino vegetal.

1.3 Material e Métodos

O Estudo foi realizado pela Universidade Federal de Rondônia e o Curtimento das peles no Curtume Texturas da Amazônia, localizado em Ji-paraná, Rondônia, Brasil. A pesquisa atendeu aos requisitos no Comitê de ética ao Uso de Animais - CEUA número 031/2018.

Foram utilizadas vinte peles retiradas de Pirarucu com peso corporal de 13,2 ± 1,4 kg provenientes de piscicultura licenciada para o cultivo da espécie, submetidas as concentrações de 5%, 10%, 15% e 20% do agente curtente tanino vegetal em cinco repetições ou peles inteiras com média de peso *in natura* de 1,1 ± 0,39/pele kg.

O curtente utilizado foi o tanino vegetal marca TANAC S.A.© extraído da Acácia negra, *Acacia mearnsii*, do Rio Grande do Sul. As peles, congeladas (-18°C), foram descongeladas, pesadas e agrupados em lotes para facilitar o cálculo das porcentagens dos produtos a serem utilizados em cada etapa no processamento. O curtimento seguiu o padrão descrito por Souza et al (2006), com algumas modificações. As etapas utilizadas foram de remolho, caleiro (10% de cal), desencalagem, purga, desengraxe, píquel, curtimento (com os níveis correspondentes de tanino vegetal), neutralização, recurtimento (mesmas concentrações utilizadas no curtimento), tingimento, engraxe, secagem e amaciamento. Para a avaliação da resistência físico-mecânica os couros não foram submetidos a etapa de tingimento. Decorrido 12 dias do curtimento dos couros, estipulou-se um horário (16h) para aferir a umidade dos couros, para tanto, foi utilizado o aparelho Drying & control systems. No final do processo de curtimento, os couros foram estabilizados em 18% de umidade.

Os testes físico-mecânicos foram realizados no laboratório de controle de qualidade da MK QUÍMICA DO BRASIL, RS em aparelho dinamômetro da marca EMIC®. A resistência físico-

mecânica dos couros secos (23ºC e 50% de umidade relativa do ar por 24 horas, conforme ABNT - NBR ISSO 4044, 2015) foi determinada a partir de 6 corpos-de-prova retirados da região dorsal de cada couro no sentido longitudinal (Figura 2) em relação ao comprimento da pele do peixe com auxilio de balancim (ABNT - NBR, 11035, 2015).

Figura 2 - Corpos-de-prova para Avaliações Físico-mecânicas dos Couros de *Arapaima gigas* Curtidos com Tanino Vegetal

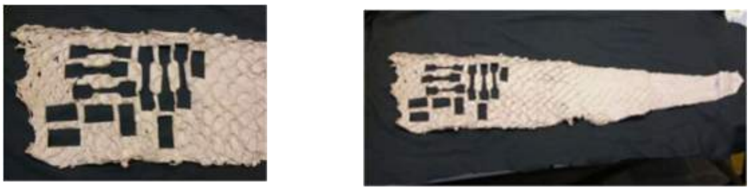

Fonte: próprio autor

Foi determinada a resistência à tração e ao alongamento (ABNT - NBR ISSO 3376, 2014) e ao rasgamento progressivo (ABNT - NBR 11055, 2014). Para os testes de determinação da resistência foram avaliadas a força (N) aplicada no teste, a tração (N/mm^2) até a ruptura do couro e para avaliação da elasticidade destes couros o teste de alongamento (%) e para o rasgamento progressivo (N/mm).

As médias dos níveis de tanino foram submetidas a Anova e a análise de regressão por meio de contrastes ortogonais para avaliar os efeitos linear, quadrático e cubico considerando-se o nível de significância de 1,0%. Para todas as análises foi utilizado o programa SAS Inst. Inc., Cary, NC, USA. (SAS, 2010).

1.4 Resultados e discussão

Os níveis de tanino vegetal influenciaram na determinação da tração e rasgamento progressivo do couro de pirarucu (P<0,01) (Tabela 4). O aumento das concentrações de tanino vegetal reduz a resistência á tração de ruptura em 3,16 N/mm^2 e aumenta o rasgamento progressivo do couro do Pirarucu em 2,08 N/mm por unidade percentual do curtente utilizado, apesar de não influenciar na elasticidade (P>0,01) ou resistência ao alongamento até ruptura.

Tabela 1. Determinação da tração, alongamento e rasgamento progressivo do couro de *Arapaima Gigas* curtidos em diferentes níveis de tanino vegetal

Tanino Vegetal (%)	Tração (N/mm^2)[1]	Alongamento (%)	Rasgamento progressivo
5%	34,10 ±9,83	86,50 ± 1,29	14,34 ± 1,25

10%	21,20 ± 21,0	73,50 ± 3,40	19,83 ± 4,92
15%	26,18 ± 5,19	91,00 ± 7,35	19,82 ± 5,55
20%	20,81 ± 0,82	82,50 ± 0,58	22,75 ± 2,08
CV (%)	15,90	9,41	6,62
Valor P	0,0011	0,7585	< 0,0001

CV: Coeficiente de variação. Equações de regressão: [1]$\hat{Y}$ =20,56-3,16 X (R^2 = 55,00), [2]$\hat{Y}$ = 17,24+2,085X (R^2 = 84,94)

O alongamento médio dos couros de Pirarucu curtidos com tanino vegetal foi de 83,87%. O arranjo estrutural das fibras colágenas da derme depende da espécie e dos agentes curtentes e permite que a pele possua grande resistência às diferentes forças de tração definindo sua utilização na confecção de vestuários ou calçados. O alongamento médio longitudinal ás fibras colágenas é de 108,43% para couro de tilápia, 111% para salmão, 58,93% para cachara (YOSHIDA et al, 2016) e de 81,82% para Bejupirá (*Rachycentron canadum)* tratados com tanino vegetal (SOUZA et al, 2017); no sentido transversal de 76,98% para o alongamento do couro do Pacu (*Piaractus mesopotamicus*) (SOUZA et al, 2008).

Portanto, através destes resultados pode-se inferir que o melhor nível de tanino vegetal a ser utilizado no curtimento do couro de pirarucu é de 15%, pois dependendo do teste realizado, a concentração proporciona uma maior dificuldade no deslizamento das fibras, fazendo com que reduza a elasticidade e diminuindo a resistência, em especial a de tração. Pode-se inferir que, para o rasgo seria possível incluir o nível de 20% de tanino vegetal, por proporcionar maior proteção as fibras colágenas, dificultando o deslizamento entre as mesmas e maior dificuldade para o rompimento dessas fibras. Contudo, a adição de 20% de tanino vegetal proporcionaria um custo mais elevado no processo de curtimento do couro.

1.5 Conclusão

A concentração de 15% de tanino vegetal é recomendada ao processo de curtimento da pele do Pirarucu, pois apresenta maior estabilidade físico-mecânica do couro para confecção de vestuários e artefatos.

1.6 Referências

ASSOCIAÇÃO BRASILEIRA DE NORMAS TÉCNICAS - ABNT. NBR 13525: **couro - Ensaios físicos e químicos em couro — Valores orientativos para aceitação de couros**. Rio de Janeiro, p.1-9. 2016.

ASSOCIAÇÃO BRASILEIRA DE NORMAS TÉCNICAS - ABNT. NBR 3377-1: **Ensaios físicos e mecânicos – Determinação da força de rasgamento. Parte 1: Rasgamento de extremidade simples**. Rio de Janeiro, p.1-4. 2014.

ASSOCIAÇÃO BRASILEIRA DE NORMAS TÉCNICAS - ABNT. NBR 11041: **couros – determinação da resistência à tração e alongamento.** Rio de Janeiro, p.1-5. 1997.

ASSOCIAÇÃO BRASILEIRA DE NORMAS TÉCNICAS - ABNT. NBR 11055*:* **couro - determinação da força de rasgamento progressivo**. Rio de Janeiro, 1997. p.1-4.

CAVALI, J; PORTO, M.O., PINHEIRO, L.M. et al Cenário das Industrias de beneficiamento do Pescado em Rondônia. In: CAVALI, J; LOPES, Y.V.A, Orgs. **Piscicultura e meio Ambiente: estudos e perspectivas na Amazônia**. 1 ed. Porto Velho: EDUFRO. 2017. Cap.8 p. 99-107.

CARDOSO, J. O design industrial como ferramenta para a sustentabilidade: estudo de caso do couro de peixe. **Revista Espaço Acadêmico***,* 10**,** 110-117. 2010.

DURAISAMY, R., SHAMENA, S. BEREKETE A.K. A review of bio-tanning materials for processing of fish skin into leather. *International Journal of Engineering Trends and Technology*. v.39, n.1, p.10-20 September 2016.

EIRAS, B. J. C. F., MEDEIROS JÚNIOR, E. F., ALVES, M. M. Desenvolvimento de método artesanal de curtimento da pele da pescada amarela (*Cynoscion acoupa*), e sua difusão por meio de oficina a uma comunidade no município de Bragança, PA, Brasil. **Semina: Ciências Agrárias,** 36**,** 1123-1134. 2015.

HOINACKI, E.; MOREIRA, M.V.; KIEFER, C.G. **Manual básico de processamento do couro**. SENAI, Centro Tecnológico do Couro, 1994. 402p.

IBGE, **Instituto Brasileiro de Geografia e Estatística**. Brasil. 2017.

KARDELL, M. TAUBE, F. SCHULZ TZE, W. GIERUS, M., Different approaches to evaluate tannin content and structure of selected plant extracts. **Journal of Applied Botany and Food Quality**. v.86, p.154 - 166. 2013.

PINTO, P.C.R., SOUSA G, CRISPIM F. SILVESTRE A.J.D. PASCOAL NETO, C. *Eucalyptus globulus* bark as source of tannin extracts for application in leather industry *ACS* **Sustainable Chem. Eng**. v.1 n.8, p 950–955. 2013. DOI**:** 10.1021/sc400037h.

SCHWARZ, K.K., MENDONÇA, K.S., WAKIUCHI S.S., SASSAMORI J.C., REBULI G.C.J.P. Metodologias para a transformação das peles de Linguado, Robalo, Paru e Tilápia em couro. **PUBVET.** v.12, n.2, p.1-8, Fevereiro, 2018. doi.org/10.22256/pubvet.v12n2a23.1-8.

SOUZA, M. L. R, GASPARINO, E. PENHA, B.G., CORADINI, M.F. GOES, E.S.R, GONÇALVES, A.A. Physicochemical and mechanical characteristics of cobia (*Rachycentron canadum*, Linnaeus, 1766) leather submitted to different tanning agents in the retanning step. **International Journal of Latest Research in Science and Technology**. v.6, n.2:p.8-13, March-April 2017.

SOUZA, M. L. R. Tecnologia para processamento das peles de peixe. **Fundamentum,** 1**,** 14-55. 2008.

SOUZA, M. L. R., CASACA, J. D. M., NAKAGHI, L. S. O., FRANCO, N. D. P., SILVA, L. O., DOURADO, D. M., VIEGAS, E. M. M. 2006. Efeito da técnica de curtimento e do método utilizado para remoção da pele da tilápia-do-nilo sobre as características de resistência do couro. **Revista Brasileira de Zootecnia***,* 35**,** 1273-1280.

VEIGA, M.C.M., MELO JR C.A.F., SANTOS, G, VIDAL, J.M.A., COSTA W.M. Extração de tanino vegetal do eucalipto no curtimento de pele de peixe: aspectos fitogeográficos. In: Jornada de Ensino, Pesquisa E Extensão – JEPEX. 8, 2013 – UFRPE: Recife. **Anais...** Recife, 2013.

VIEIRA, A. M., KACHBA, Y. R., FRANCO, M. L. R. S., OLIVEIRA, K. F., GODOY, L. C., GASPARINO, E. Curtimento de peles de peixe com taninos vegetal e sintético. **Acta Scientiarum Animal Sciences,** 30**,** 359-363. 2008.

YOSHIDA, G. M., KUNITA, N. M., SOUZA, M. L. R., GASPARINO, E. Análises mecânicas e físico-químicas de couros de tilápia, cachara e salmão. **Archivos de Zootecnia***,* 65**,** 349-355. 2016.

CAPITULO II

2 CARACTERISTICAS DO COURO DO PIRARUCU CURTIDO COM EXTRATOS DE ORIGEM VEGETAL EM SUBSTITUIÇÃO AOS SAIS DE CROMO

RESUMO

A piscicultura é uma das novas atividades do meio rural, que vem atraindo muitos ex-produtores e novos empresários agrícolas, isto está ocorrendo, devido às altas taxas de retorno e de lucratividade, quando comparado às outras opções de investimentos (Martins et al., 2001). Mas últimos anos o impacto ambiental vem sendo muito discutido no setor dos curtumes que utilizam as peles, que são subprodutos da indústria pesqueira. O cromo, por sua vez é o agente curtente mais utilizado e considerado um dos mais impactantes. Dessa forma, segundo Gutterres (1997), buscam-se alternativas tecnológicas para substituição do cromo por outros agentes de curtimento (curtentes vegetais). A resistência do couro está diretamente relacionada com o tipo de agente curtente utilizado durante o processo. Neste sentido, objetivo deste trabalho foi comparar a resistência em diferentes sentidos dos couros de pirarucu curtidos com sais de cromo e tanino vegetal. O Estudo foi realizado pela Universidade Federal de Rondônia e o Curtimento das peles no Curtume Texturas da Amazônia, em março de 2017, de localizado em Ji-paraná, Rondônia, Brasil. A pesquisa atendeu aos requisitos no Comitê de ética ao Uso de Animais - CEUA número 031/2018. Para o processo de curtimento e coloração foram utilizadas 42 ½ peles com média de peso in natura de 1,0 ± 0,40 kg de 21 Pirarucus de 12 ± 0,5 kg, distribuídas em delineamento inteiramente casualizado em fatorial 2 x 3 sendo, 2 curtentes (sais de cromo e tanino vegetal, Acacia mearnsii) e 2 corantes (químico e urucum), com 7 repetições para cada tratamento. Foi determinada a resistência à tração e ao alongamento (ABNT – NBR ISO 3376, 2014) e ao rasgamento progressivo (ABNT – NBR 11055, 2014). Para os testes de determinação da resistência foram avaliadas a força (N) aplicada no teste, a tração (N/mm2) até a ruptura do couro e para avaliação da elasticidade destes couros o teste de alongamento (%) e para o rasgamento progressivo (N/mm). Estas análises de determinação de resistência dos couros foram realizadas pelo Laboratório de Processamentos de Peles e Couros da Universidade Estadual de Maringá, localizado na Fazenda Experimental de Iguatemi (Iguatemi-PR). A elasticidade (%) e a deformação (mm) do couro não foram influenciadas pelo uso dos diferentes agentes curtentes, mas sim pelos sentidos de retirada dos corpos de prova. Os couros no sentido transversal (60,79%) apresentaram maior elasticidade comparada ao sentido diagonal (45,93%), enquanto o longitudinal (57,78%) não diferiu dos sentidos. Quanto a deformação

houve um aumento no comprimento do corpo de prova após a determinação da elasticidade do couro de 3,7 cm para o sentido transversal, 3,4 cm para o longitudinal e 2,7 cm para o diagonal. Os couros curtidos com sais de cromo apresentaram maior resistência (7,28 N/mm2) comparada aos com tanino (5,32 N/mm2). Isso refletiu em 26,92% a mais de resistência, quando utilizado os sais de cromo como agente curtente. Quando analisado o sentido do couro, o longitudinal apresentou menor resistência a tração (4,66 N/mm2) em relação aos demais, ou seja, o corpo de prova foi 14,71% menos resistente com relação ao sentido transversal e 63,25% ao diagonal.

Palavras-chaves: impacto ambiental. tração físico-mecânica. alongamento.

ABSTRACT

Fish farming is one of the new activities in the rural environment, which has attracted many ex-producers and new agricultural entrepreneurs. This is happening because of the high rates of return and profitability when compared to other investment options (Martins et al. 2001). But in the last years the environmental impact has been much discussed in the sector of tanneries that use the skins, which are by-products of the fishing industry. Chromium, in turn, is the most used tanning agent and considered one of the most impacting. Thus, according to Gutterres (1997), technological alternatives are sought to replace chromium by other tanning agents (vegetable tanning agents). The strength of the leather is directly related to the type of tanning agent used during the process. In this sense, the objective of this work was to compare the resistance in different senses of the leather of pirarucu tanned with salts of chromium and vegetable tannin. The study was carried out by the Federal University of Rondônia and the tanning of the skins in Curtume Textures of the Amazon, in March 2017, located in Ji-paraná, Rondônia, Brazil. The research met the requirements in the Ethics Committee on the Use of Animals - CEUA number 031/2018. For the tanning and coloring process, 42 ½ skins with a mean in natura weight of 1.0 ± 0.40 kg of 21 Pirarucus of 12 ± 0,5 kg were used, distributed in a completely randomized design in factorial 2 x 3, 2 tannins (salts of chromium and vegetable tannin, Acacia mearnsii) and 2 dyes (chemical and urucum), with 7 replicates for each treatment. The tensile strength and elongation (ABNT - NBR ISO 3376, 2014) and progressive tearing (ABNT - NBR 11055, 2014) were determined. The strength (N) applied in the test, the traction (N / mm2) until the leather rupture and the elasticity evaluation of these hides were evaluated for the test of elongation (%) and progressive tear (N / mm). These analyzes of resistance determination of the hides were performed by the Leather and Leather Processes Laboratory of the State University of Maringá, located at the Experimental Farm of Iguatemi (Iguatemi-PR). The elasticity (%) and the deformation (mm) of the leather were not influenced by the use of different tanning agents, but by the senses of removal of the specimens. The hides in the transverse direction (60.79%) presented greater elasticity compared to the diagonal direction (45.93%), while the longitudinal one (57.78%) did not differ from the senses. As for deformation, there was an increase in the length of the specimen after determination of the elasticity of the leather of 3,7 cm for the transverse direction, 3,4 cm for the longitudinal and 2.7 cm for the diagonal. The tanned hides with chromium salts presented higher resistance (7.28 N / mm2) than those with tannin (5.32 N / mm2). This reflected 26.92% more resistance when using the chromium salts as a tanning agent. When the direction of the leather was analyzed,

the longitudinal showed a lower tensile strength (4.66 N / mm2) in relation to the others, that is, the specimen was 14.71% less resistant with respect to the transverse direction and 63.25 % to diagonal.

Key words: environmental impact. physical-mechanical traction. stretching.

2.1 Introdução

A exigência de incrementar a produção de alimentos para o consumo humano tem despendido esforços no sentido de viabilizar alternativas viáveis em quantidade e qualidade, e nesse sentido a aquicultura tem se mostrado adequada para essa finalidade.

O crescimento da aquicultura no Brasil se deve a fatores como investimentos no setor de produção/processamento, ao incremento nas pesquisas/extensão, aliado aos vastos recursos hídricos disponíveis. E entre as espécies de peixes nativos cultivados, vem ganhando grande interesse o pirarucu, pela sua qualidade da carne e excelente aceitação pelos consumidores.

A pele de peixe pode variar entre 4,5% a 14% em relação ao peso corporal, podendo a mesma ser transformada em couro (SOUZA e SILVA, 2005). A pele processada em couro pode ser utilizada posteriormente como matéria-prima para confecção de sapatos, bolsas, carteiras, cintos, jaquetas, entre outros.

Para realizar o curtimento das peles, seja qual for a espécie, pode ser utilizado diversos tipos de agentes curtentes, entre eles, sais de cromo, tanino vegetal ou sintético, alumínio, assim como para o tingimento, uso de corantes químicos especiais para o couro ou tecidos e naturais de diferentes origens. A utilização de agentes curtentes de origem mineral tem sido preteridas, como é o caso do cromo, pois os mesmos podem ser danosos ao meio ambiente, levando muitos países a dar preferência comercial aos couro que utilizaram taninos vegetais (GONDIN et al., 2015).

De acordo com Kasim et al. (2014) o cromo é o agente mineral mais utilizado mundialmente, cerca de 90% dos curtumes fazem uso deste produto. Com isso, a contaminação ambiental proveniente de efluentes industriais tem agravado a situação de degradação do meio ambiente, e dentre os metais tóxicos, o que apresenta maior destaque é o cromo (DAL MAGRO *et al.,* 2013). Além disso, elevadas concentrações de cromo podem trazer impactos diretos nos fatores fisiológicos e bioquímicos de funções vitais no corpo humano (GHANI, 2011).

Dessa forma se faz necessário avaliar a possibilidade de utilização de taninos vegetais em substituição aos sais de cromo no curtimento da pele de pirarucu.

Neste sentido, objetivou-se avaliar a resistência físico-mecânica nos diferentes sentidos das fibras dos couros de Pirarucu submetidos ao curtimento com óxido de cromo e tanino vegetal.

2.2 Objetivo Geral

O objetivo deste trabalho foi comparar a resistência em diferentes sentidos dos couros de pirarucu curtidos com sais de cromo e tanino vegetal

2.2.1 Específicos

Avaliar os parâmetros fisico-mecânicos e químicos dos couros curtidos com tanino vegetal em substituição aos sais de cromo;

Avaliar a resistência dos couros nos diferentes sentidos (longitudinal, transversal e diagonal) ao comprimento do corpo do peixe em função dos tipos de agentes curtentes utilizados.

2.3 Material e Métodos

O Estudo foi realizado pela Universidade Federal de Rondônia e o Curtimento das peles no Curtume Texturas da Amazônia, em março de 2017, de localizado em Ji-paraná, Rondônia, Brasil. A pesquisa atendeu aos requisitos no Comitê de ética ao Uso de Animais - CEUA número 031/2018.

Para o processo de curtimento e coloração foram utilizadas 42 ½ peles com média de peso *in natura* de 1,0 ± 0,40 kg de 21 Pirarucus de 12 ± 0,5 kg, distribuídas em delineamento inteiramente casualizado em fatorial 2 x 3 sendo, 2 curtentes (sais de cromo e tanino vegetal, *Acacia mearnsii*) e 2 corantes (químico e urucum), com 7 repetições para cada tratamento.

As peles, congeladas (-18°C), foram descongeladas e agrupados em lotes com média de 14 kg, e também o cálculo das porcentagens dos produtos a serem utilizados em cada etapa no processamento. O curtimento seguiu o padrão descrito por Souza (2004), com algumas modificações. As etapas utilizadas foram de remolho, caleiro (10% de cal), desencalagem, purga, desengraxe, píquel, curtimento (15% de tanino vegetal ou 5% de sais de cromo), neutralização, recurtimento (mesmas concentrações utilizadas no curtimento), tingimento, engraxe, secagem e amaciamento (Figura 1).

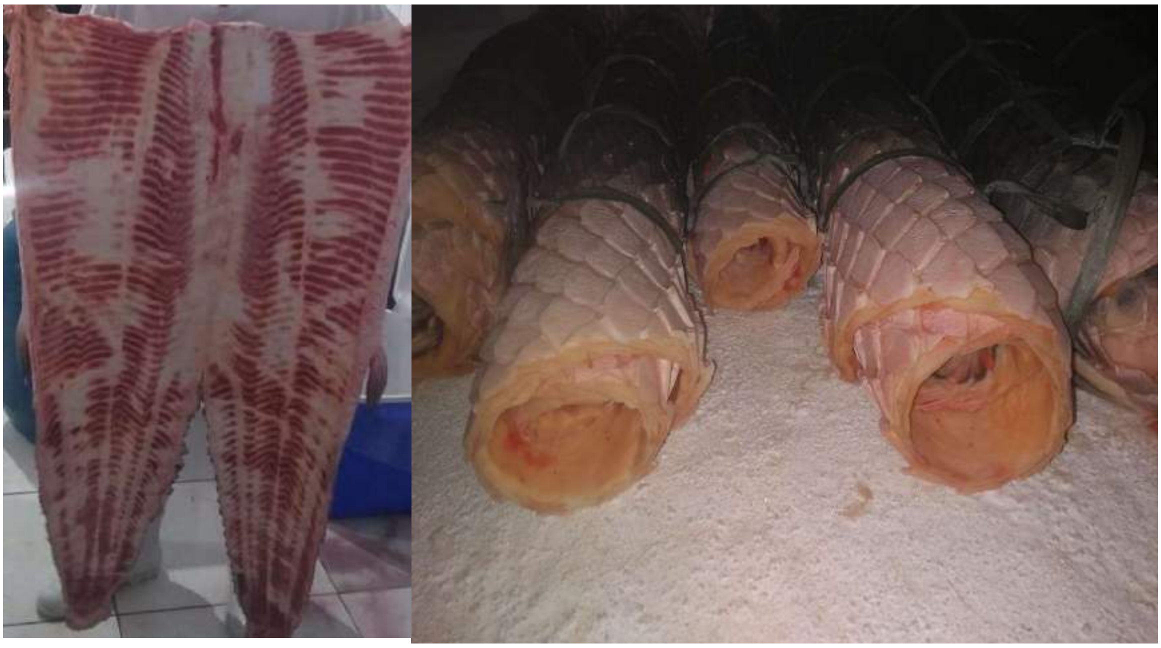

Figura 1 – Pele de Pirarucu após a Retirada do Peixe, com Resíduos de Carne no Lado Carnal (A) e Peles com Escamas Prontas para o Congelamento (B)

As peles foram processadas em tempos diferentes, ou seja, foi utilizado um fulão para o curtimento com tanino vegetal e depois com sais de cromo. Decorrido 12 dias do curtimento dos couros, estipulou-se um horário (16h) para aferir a umidade dos couros, para tanto, foi utilizado o aparelho Drying e control systems. Foram utilizados os mesmos produtos e mesma formulação, exceto no píquel e curtimento, para adição de tanino vegetal e sais de cromo.

No final do processo de curtimento, os couros foram estabilizados em 18% de umidade, mensurando-se a área, peso e espessura do couro com espessímetro para posteriores análises.

2.3.1 Testes Físico-mecânicos dos Couros

Após curtimento, os couros secos foram encaminhados a um laboratório climatizado (23ºC e 50% de umidade relativa do ar) por 24 horas, conforme ABNT (NBR ISO 4044, 2015). Foram retirados os corpos de prova por tratamento (curtimento com tanino vegetal e sais de cromo) com auxilio de balancim (ABNT– NBR, 11035, 2015a), e com um espessímetro foi medido em dois pontos as espessuras (Figura 3) de cada corpo de prova (ABNT – NBR ISO 2418, 2015). Os corpos de prova foram retirados da região dorsal do couro do pirarucu curtidos pelos dois agentes curtentes, no sentido longitudinal, transversal e diagonal ao comprimento do corpo do peixe (Figura 4).

(A) 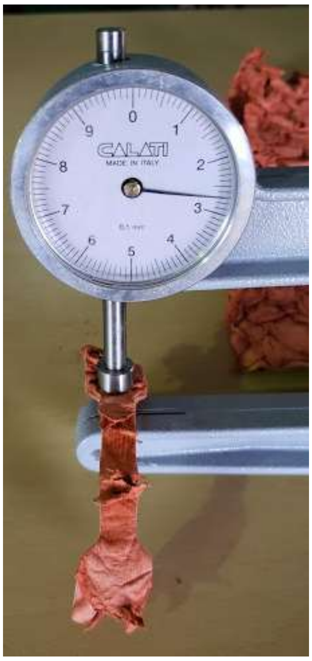 (B) 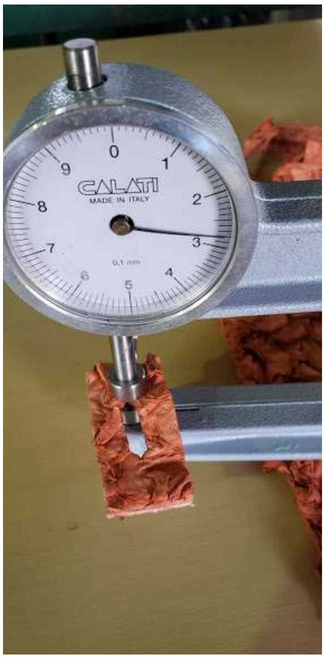

Figura 3 – Ponto de medição da espessura do corpo de prova de tração e alongamento (A) e rasgamento progressivo (B) do couro de Pirarucu com auxílio de espessímetro.

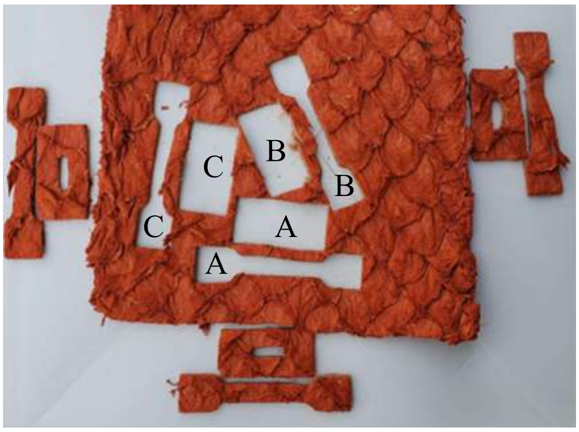

Figura 4 - Couro de Pirarucu com a retirada dos corpos de prova utilizados para os testes de determinação da tração e alongamento (*) e rasgamento progressivo (**). Corpos de prova retirados nos sentidos longitudinal (A), transversal (B) e diagonal (C) ao comprimento do corpo do pirarucu.

Foi determinada a resistência à tração e ao alongamento (ABNT – NBR ISO 3376, 2014) e ao rasgamento progressivo (ABNT – NBR 11055, 2014). Para os testes de determinação da resistência foram avaliadas a força (N) aplicada no teste, a tração (N/mm^2) até a ruptura do couro e para avaliação da elasticidade destes couros o teste de alongamento (%) e para o rasgamento progressivo (N/mm).

Para os testes de resistência, foi utilizado dinamômetro da marca EMIC, com velocidade

de afastamento entre cargas de 100 ± 20 mm/mm. Foi utilizada uma célula de carga de 200 kgf e a calibração foi realizada pela Emic-dcame, laboratório de calibração credenciado pela CGCRE/inmetro sob nº 197.

Estas análises de determinação de resistência dos couros foram realizadas pelo Laboratório de Processamentos de Peles e Couros da Universidade Estadual de Maringá, localizado na Fazenda Experimental de Iguatemi (Iguatemi-PR).

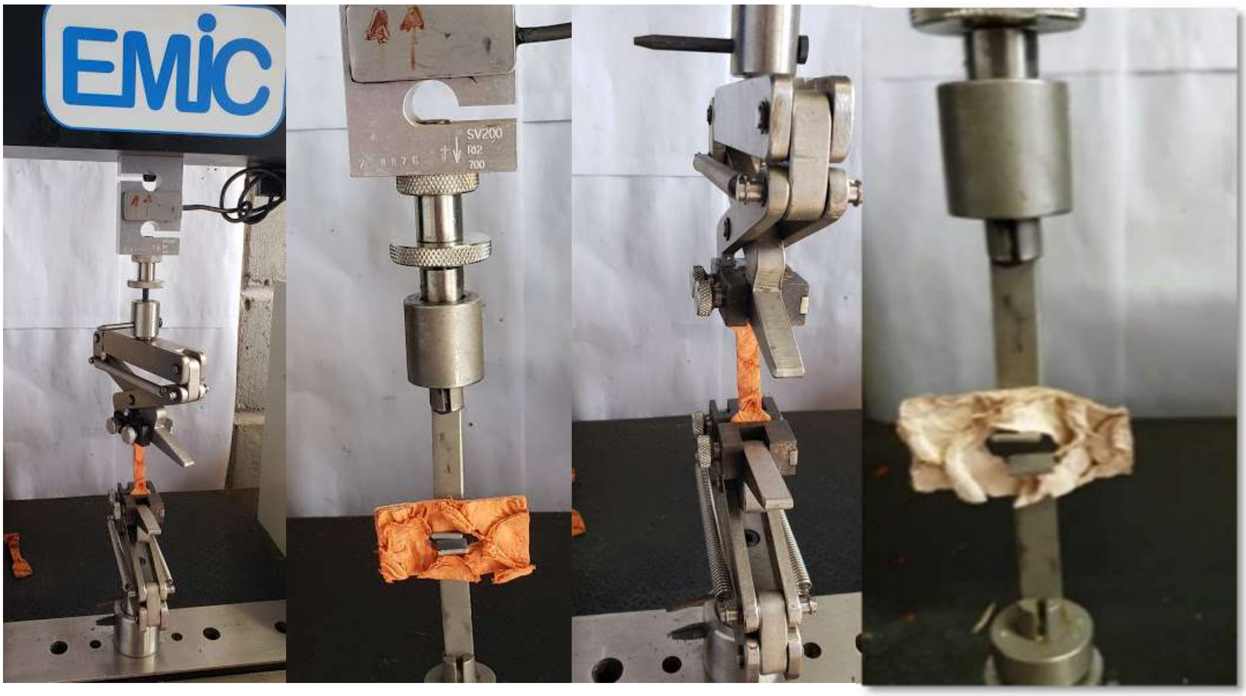

Figura 5. Realização dos testes de rasgamento progressivo (A) e tração ao alongamento (B).

2. 4 Análise Histológica do Couro de Pirarucu

Para as análises histológicas foram coletadas amostras de cada sentido do couro do pirarucu curtido com tanino vegetal e emblocado em parafina para realização dos cortes. As amostras foram cortadas com aproximadamente 5 micrômetros de espessura e coradas pela técnica de hematoxilina-eosina (HE) (LUNA, 1968) para descrever a histologia da derme. Os cortes histológicos foram analisados pela microscopia de luz e fotografados em fotomicroscópio Calrs Zeiss/AxioLab e AxioxKop Zeiss.

2.5 Análise de Microscopia Eletronica de Varredura

Amostras nos diferentes sentidos do couro em relação ao comprimento do peixe foram retiradas para análise da distribuição das fibras colágenas da derme e lamélulas de inserção e proteção das escamas. Pequenas amostras foram retiradas nos diferentes sentidos (longitudinal, transversal e diagonal), na superfície da flor do couro e na face do carnal. As amostras foram simplesmente fixadas com fits adesiva sobre os stabs para a análise no microscópio eletrônico de varredura SHIMADZU-SS550, disponibilizados pelo Complexo Central de Apoio à Pesquisa (COMCAP-UEM). As amostras não foram metalizadas, pois utilizou-se baixo vácuo.

2.6 Composição Centesimal da Pele e dos Couros de Pirarucu

Peles foram salgadas para serem transportadas de Rondônia à Maringá, para a realização da análise de composição centesimal. As amostras foram embaladas em sacos plásticos e identificadas. Após chegada ao laboratório da Universidade Estadual de Maringá as peles foram dessalgadas e realizada a análise de composição química. Amostras dos couros em etapa de piquel e curtidas também foram analisadas. Para análise de umidade e de cinzas foi utilizada a metodologia oficial do AOAC (2005), a quantidade de proteína bruta foi avaliada pelo método de semi-micro Kjeldahl (SILVA e QUEIROZ, 2002) e a extração de lipídeos totais segundo Bligh e Dyer (1959). Os teores de carboidratos foram estimados utilizando-se uma fórmula matemática que considera a soma dos valores de umidade, proteína, lipídios e cinzas subtraído de 100%

2.6.1 Análises Físico-química dos Couros de Pirarucu

A preparação de amostras de couro para análise química seguiu as condições exigidas pelas normas da ABNT (NBR ISSO 4044, 2015), para a determinação do óxido de cromo Cr_2O_3 (ABNT - NBR 10054, 2014), determinação das substâncias extraíveis com diclorometano (CH_2Cl_2) (ABNT - NBR 11030, 2013) e a determinação do pH e da cifra diferencial do pH de um extrato aquoso (ABNT- NBR 10455, 2014).

2.6.2 Analise Estatística

Os dados obtidos foram submetidos à análise de variância usando o PROC GLM do SAS (2010) e às médias comparadas ao nível de significância de 5% pelo teste de Tukey.

2.7 Resultados e Discussões

Os couros de pirarucu apresentaram após o curtimento uma maior espessura referente a região dorsal do animal e mais delgada na região ventral. Através das análises nota-se a redução da espessura da região dorsal (2,9 - 3,8mm) para a região ventral (2,0-2,9mm). Na região central do couro a espessura média variou de 1,8 a 2,8mm. As lamélulas de proteção e inserção das escamas são longas e profundas e espessas (Figura 6a). A variação da espessura dessas lamélulas vai de 0,45 nas laterais e 1mm, no centro, que corresponte ao ponto de junção entre as três lamélulas.

2.8 Análise físico-mecânica dos couros de pirarucu curtidos com tanino vegetal e Sais de Cromo no Sentido Longitudinal, Transversal e Diagonal

As espessuras médias dos corpos de prova dos couros de Pirarucu variaram de 1,79mm a 2,82mm (Tabelas 1 e 2). Quando analisada a espessura dos corpos de prova utilizados para os testes de tração e alongamento houve interação ($P<0,01$) para o agente curtente e sentido de retirada desses corpos de prova. Com o desdobramento dessa interação, pode-se inferir que os couros com tanino vegetal apresentaram-se mais espessos ($P<0,05$) que os curtidos com sais de cromo, ou seja, o tanino vegetal proporcionou um couro com 24,59% mais espesso em relação aos curtidos com sais de cromo. Portanto, o tanino vegetal proporcionou uma maior espessura ao couro, deixando o couro mais encorpado.

Quanto ao sentido de retirada dos corpos de prova, os sentidos longitudinal e transversal apresentaram significativamente menor espessura quando curtidos com sais de cromo, enquanto sentido diagonal não diferiu apenas do longitudinal quando curtido com tanino vegetal.

2.8.1 Curtimento com Tanino Vegetal e Sais de Cromo

As espessuras médias dos corpos de prova dos couros de Pirarucu variaram de 1,79mm a 2,82mm (Tabelas 1 e 2). Quando analisada a espessura dos corpos de prova utilizados para os testes de tração e alongamento houve interação para o agente curtente e sentido de retirada

desses corpos de prova. Com o desdobramento dessa interação, pode-se inferir que os couros com tanino vegetal, apresentaram-se significativamente mais espesso que os curtidos com sais de cromo, ou seja, o tanino vegetal proporcionou um couro com 24,59% mais espesso em relação aos curtidos com sais de cromo. Portanto, o tanino vegetal proporcionou uma maior espessura ao couro, deixando o couro mais encorpado.

Quanto ao sentido de retirado dos corpos de prova, apenas, o sentido diagonal dos couros curtidos com sais de cromo não diferiu significativamente do sentido transversal e diagonal dos couros curtidos com tanino vegetal (Tabela 1).

Resultados semelhantes foram obtidos na resistência da pele de Rã-Touro (*Rana catesbeiana*), curtidos com sais de cromo e tanino vegetal. O tanino vegetal proporcionou couros mais espessos (longitudinal = 0,74mm e transversal = 0,81mm) que os curtidos com sais de cromo (longitudinal 0,74mm e transversal = 0,71mm). No entanto, foi significativo apenas para o couro no sentido transversal (tanino = 0,81mm e cromo = 0,71mm) (HILBIG et al. 2010)

A força aplicada no teste de tração e alongamento não apresentou diferença significativa para os agentes curtentes e sentido do couro (Tabela 1), cujos valores variaram de 110 N a 141N para ocorrer a ruptura do corpo de prova.

A elasticidade (%) e a deformação (mm) do couro não foram influenciadas pelo uso dos diferentes agentes curtentes, mas sim pelos sentidos de retirada dos corpos de prova. Os couros no sentido transversal (60,79%) apresentaram maior elasticidade comparada ao sentido diagonal (45,93%), enquanto o longitudinal (57,78%) não diferiu dos sentidos. Quanto a deformação houve um aumento no comprimento do corpo de prova após a determinação da elasticidade do couro de 3,7 cm para o sentido transversal, 3,4 cm para o longitudinal e 2,7 cm para o diagonal (Tabela 1).

Para a determinação da tração houve diferença significativa para os agentes curtentes e sentido de retirada dos corpos de provas ($P<0,05$). Os couros curtidos com sais de cromo apresentaram maior resistência (7,28 N/mm^2) comparada aos com tanino (5,32 N/mm^2). Isso refletiu em 26,92% a mais de resistência, quando utilizado os sais de cromo como agente curtente. Quando analisado o sentido do couro, o longitudinal apresentou menor resistência a tração (4,66 N/mm^2) em relação aos demais, ou seja, o corpo de prova foi 14,71% menos resistente com relação ao sentido transversal e 63,25% ao diagonal.

Quando analisado o rasgamento progressivo dos couros de pirarucu, os corpos de prova dos couros curtidos com tanino vegetal necessitaram de 25,42% a mais de força máxima para o rasgo em comparação as curtidas com sais de cromo. A determinação do rasgo, independe dos agentes curtentes ($P>0,05$), pois estes não influenciaram na resistência dos couros (Tabela

2). Porém, a força máxima e média aplicadas apresentaram diferença, ou seja, os couros curtidos com tanino vegetal necessitaram de maior de força para terminar de rasgar os corpos de prova. No sentido do couro, os corpos de prova retirados no sentido transversal exigiram menor força para o rasgo ($P<0,05$), por apresentar menor quantidade de fibras de reticulina (Figura 10A).

Tabela 1 - Tração e alongamento dos couros de Pirarucu curtidos com tanino vegetal e sais de cromo nos sentidos longitudinal, transversal e diagonal

	Couro	Espessura (mm)	Força (N)	Tração (N/mm²)	Deformação (mm)	Alongamento (%)
Tanino	Longitudinal	2,76±0,42a[1]	154,71±97,96	4,48±0,84	33,00±14,51	55,00±24,81
	Transversal	2,20±0,34ab	108,28±31,36	4,82±1,24	35,29±4,39	59,00±7,04
	Diagonal	2,36±0,25ab	160,43±61,47	6,65±2,13	29,29±3,99	48,57±6,77
Cromo	Longitudinal	1,77±0,26c	95,71±37,95	4,71±1,72	35,71±11,10	59,57±18,49
	Transversal	1,82±0,27c	125,29±59,83	8,49±2,71	37,86±9,89	62,57±15,86
	Diagonal	1,94±0,37b	109,29±71,34	8,61±1,53	26,00±6,40	43,29±10,42
			Efeitos Principais			
Curtente	Tanino	2,44±0,40	141,14±69,87	5,32±1,73b	32,52±8,95	54,19±15,26
	Cromo	1,84±0,2	110,00±67,43	7,28±3,24a	33,19±10,35	55,14±16,91
Sentido	Longitudinal	2,26±0,62	125,21±56,45	4,68±1,30b	34,36±12,53ab	57,28±21,16ab
	Transversal	2,01±0,35	116,79±46,73	6,66±2,78a	36,57±7,47a	60,79±11,94a
	Diagonal	2,16±0,35	139,85±69,26	7,64±2,05a	27,64±5,40b	45,93±8,88b
			Valores de P			
Curtimento (C)		0,0001	0,1240	0,0012	0,8160	0,8418
Sentido (S)		0,1183	0,7569	0,0003	0,0388	0,0381
C x S		0,0244	0,2370	0,0533	0,621	0,6492
C.V.[2] (%)		14,71	50,84	34,63	28,05	28,08

[1]médias ± desvio padrão seguidas na mesma coluna pelo teste de Tukey a 5% de probabilidade; [2]CV = Coeficiente de Variação

Tabela 2. Determinação do rasgamento progressivo dos couros de Pirarucu curtidos com tanino vegetal e sais de cromo nos sentidos longitudinal, transversal e diagonal

Couro		Espessura (mm)	Rasgo (N/mm)	Força máxima (N)	Força média (N)
Tanino	Longitudinal	2,82±0,63	44,99±14,36	125,57±44,12	101,00±3579
	Transversal	2,76±0,42	31,14±8,29	83,42±25,96	60,14±18,34
	Diagonal	2,81±0,37	43,60±10,98	121,57±26,19	95,92±25,62
Cromo	Longitudinal	2,15±0,59	39,41±8,94	84,12±22,15	66,37±20,67
	Transversal	1,85±0,44	37,69±9,70	70,28±23,54	53,85±17,76
	Diagonal	2,16±0,43	43,40±9,99	91,85±19,95	71,14±11,85
Efeitos Principais					
Curtente	Tanino	2,80±0,46a[1]	39,92±12,63	110,19±37,03a	85,52±32,03a
	Cromo	2,06±0,49b	40,14±9,37	82,18±22,71b	63,91±18,06b
Sentido	Longitudinal	2,46±0,68	42,02±11,69	103,47±39,22^{a}	82,53±32,90^{a}
	Transversal	2,31±0,63	34,42±9,32	76,86±24,77b	57,00±17,65b
	Diagonal	2,49±0,51	43,51±10,09	106,71±27,17a	83,29±22,95^{a}
Valores de P					
Curtimento (C)		0,0001	0,9370	0,0022	0,0036
Sentido (S)		0,5550	0,0600	0,0116	0,0043
C x S		0,7569	0,3117	0,4040	0,2567
C.V.[2] (%)		20,44	26,29	29,20	30,73

[1]médias ± desvio padrão seguidas na mesma coluna pelo teste de Tukey a 5% de probabilidade; [2]C.V. = Coeficiente de Variação.

As fibras de reticulina são mais finas e amarram os feixes de fibras colágenas, providenciam suporte estrutural para os diversos tecidos e órgãos, como fibras musculares, nervos, vasos sanguíneos bem como sob a lâmina basal epitelial e também formam a estrutura de órgãos hematopoiéticos, estroma da medula óssea, do baço e dos gânglios linfáticos, exceto o timo. São compostas por colágeno tipo III, sendo as fibras de menor diâmetro no tecido conjuntivo. Estas são sintetizadas por diversas células do nosso organismo, como os fibroblastos, as células reticulares, as células de *Schwann* e as células musculares, compõem a maior parte do componente fibroso e apresenta células de forma estrelada com finos prolongamentos.

De acordo com Hoach et al. (2009) a técnica de curtimento, o tipo e percentagem do agente curtente e a etapa em que eles foram utilizados (curtimento ou recurtimento), influenciam nos resultados de resistência dos couros. Também é interessante comparar a resistência dos couros de pirarucu com os couros de outras espécies de peixes e, até mesmo outros animais para verificar a qualidade desse couro inovador que é a pele de pirarucu para sua aplicação em confecções entre outros.

Hoch et al. (2008), ao avaliarem os efeitos de diferentes agentes curtentes em peles de coelho, o tratamento que foi utilizado o cromo proporcionou maior elasticidade ao couro.

Souza et al. (2003) analisando couros de pacu, com espessura variando de 0,73 a 0,88 mm, obtiveram valores de tração para os sentidos longitudinal e transversal de 5,93 N/ mm^2 e 13,81 N/mm^2, para o rasgamento progressivo de 15,66 N/mm e 13,85 N/mm e para o alongamento de 52,20 e 76,98 %, respectivamente. Neste trabalho do pirarucu observou-se valores de tração foram inferiores aos encontrados por Souza et al. (2003), porém para o rasgo os couros de pirarucu apresentaram os valores médios maiores, portanto, mais resistente. A elasticidade dos couros de pirarucu foi bem próxima dos valores do couro de pacu.

Rocha (2007), analisando a pele de tambaqui submetida ao curtimento com tanino vegetal e sais de cromo, relatou valores médios de 13,86 N/mm^2 e 8,39 N/mm^2 para o teste de tração, 55,64 % e 34,64 % para alongamento e 34,85 e 22,64 N/mm para rasgamento progressivo, respectivamente. Comparando com os resultados obtidos no couro de pirarucu com os referidos autores, a elasticidade do couro de tambaqui foi menor, e com mais facilidade de rasgar (Tabela 2). Mas, comparando a resistência dos couros em função do agente curtente, nota-se que o couro de tambaqui quando curtido com tanino vegetal apresentou maior resistência que o de pirarucu. No entanto, deve-se avaliar além do agente curtente, a espécie de couro devido à arquitetura histológica das fibras colágenas, concentração de produtos utilizados, em especial a quantidade ou percentual de tanino utilizado por quilos de pele in natura. Já quando curtido com sais de

cromo a resistência do couro do pirarucu foi muito próximo ao obtido para tambaqui.

2.8.2 Análise da Morfologia do Couro de Pirarucu

Após o processo de curtimento pode-se observar o desenho de flor do couro do pirarucu (Figura 6a). Observa-se que acima de cada lamélula, no ponto de junção entre as três lamélulas de inserção e proteção das escamas (Figura 6b) encontra-se um orífico correspondente a linha lateral que percorre o couro no sentido ao comprimento do corpo de peixe. Ao redor do orífico encontra-se feixes de fibras colágenas finas organizadas no sentido horizontal a derme e outras entrelaçando entre elas (Figuras 6c, d).

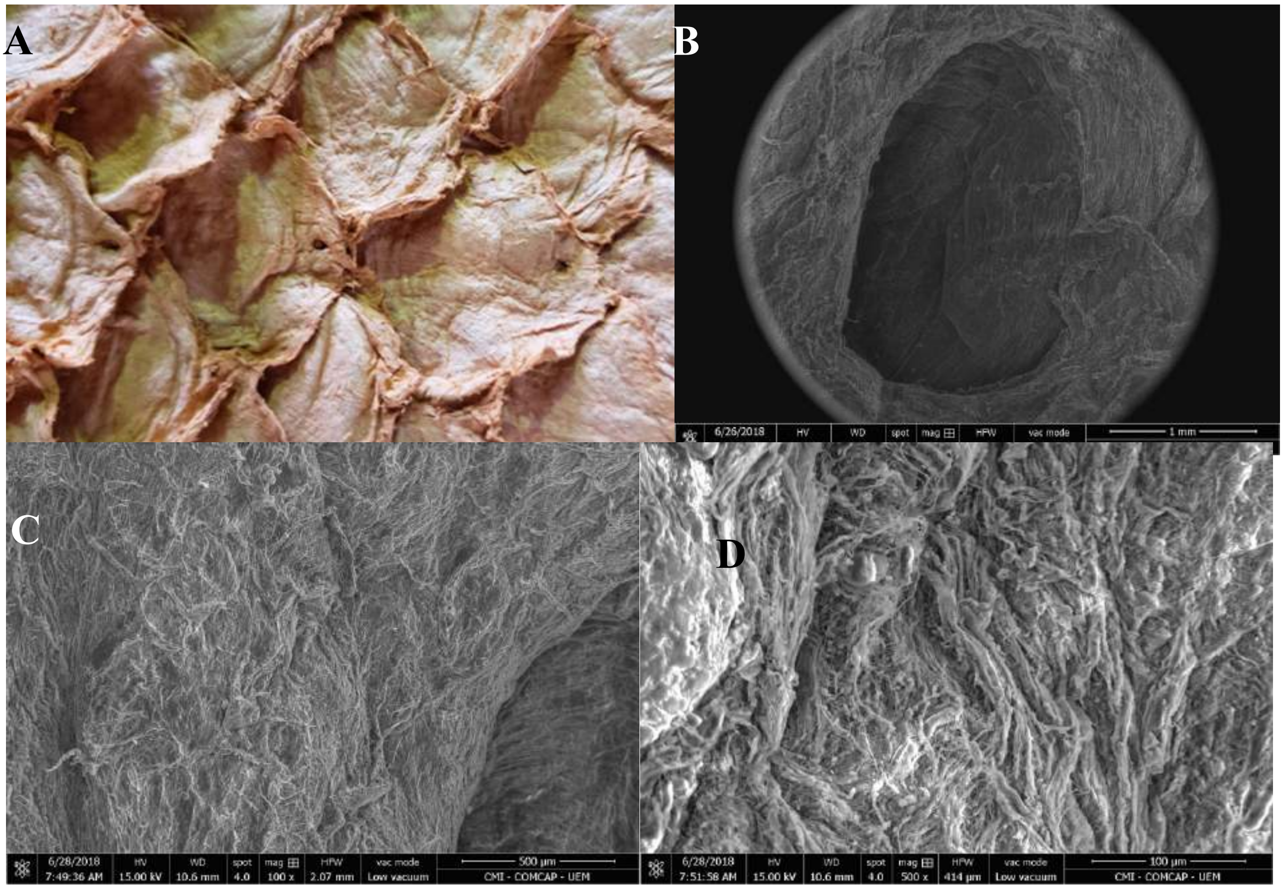

Figura 6 (a) Superfície do couro de pirarucu evidenciando as lamélulas de proteção e inserção das escamas (ponto de junção entre três lamélulas *), (b) Microscopia eletrônica de varredura do orifício da linha lateral, e ao redor (c, d) mostrando as fibras colágenas finas e sem a camada epidérmica.

O couro do pirarucu apresenta camadas sobrepostas de feixes de fibras colágenas intercaladas e entrelaçadas entre si. Na Figura 7 mostra o lado carnal do couro, após o curtimento e na Figura 8 (a, b) a microscopia eletronica de varredura deste mesmo local para

observar com melhor nitidez essa organização do entrelaçamento dos feixes de fibras colágenas.

Segundo Souza et al (2013) a resistência do couro de tilápia do Nilo deve-se primeiramente à estrutura histológica da pele, ou seja, à arquitetura dos feixes de fibras colágenas na derme profunda. Neste caso, forma-se um arranjo de camadas de fibras colágenas sobrepostas paralelamente à superfície da pele e intercaladas perpendicularmente a essa, formando uma amarração entre essas fibras colágenas, que permite conseqüentemente, maior resistência ao couro curtido.

De acordo com Pasos (2002), em geral em peixes, a derme consiste em uma relativa camada de tecido difuso, zona denominada estrato compacto. Essa é rica em fibras de colágeno, que se encontram dispostas em forma paralela à flor da pele e entrecruzadas entre si em lâminas ou camadas, no formato de redes entrecruzadas como nos mamíferos.

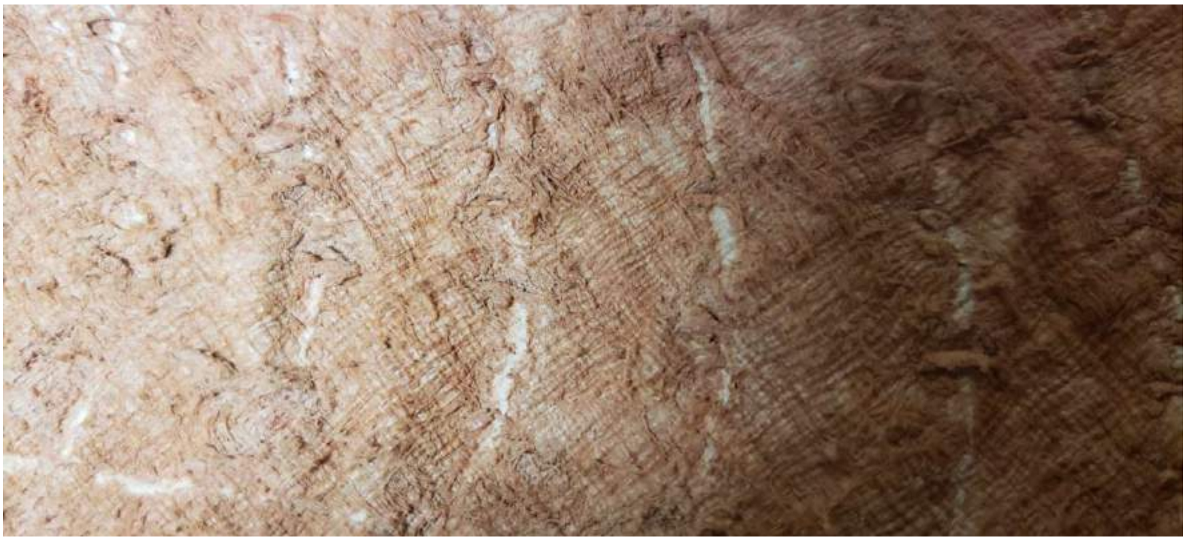

Figura 7. Couro de pirarucu após o curtimento, evidenciando a disposição dos feixes de fibras colágenas observada pelo lado carnal do couro.

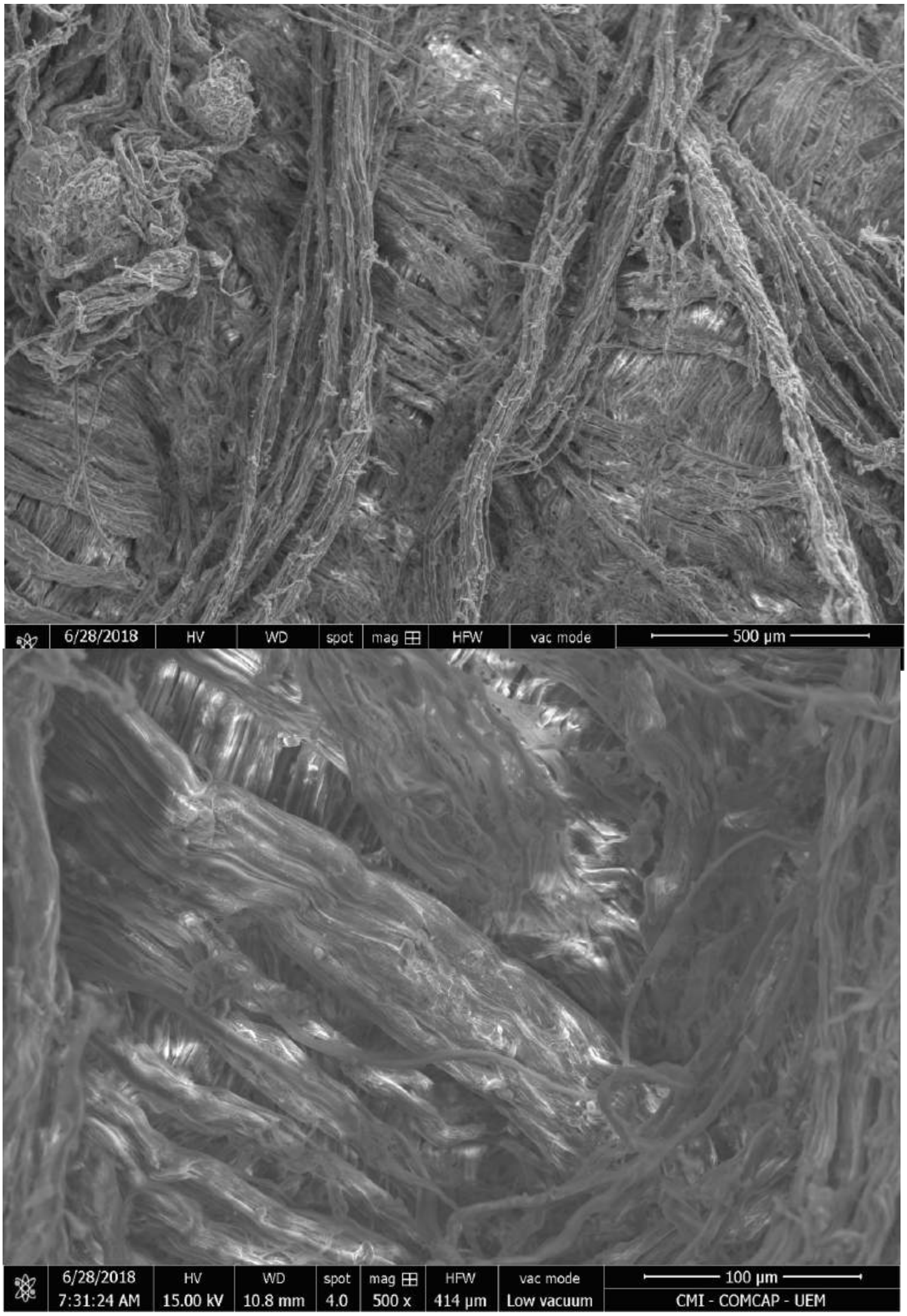

Figura 8 (a, b). Microscopia Eletrônica de Varredura mostrando à disposição e orientação dos feixes de fibras colágenas do lado do carnal do couro de pirarucu em camadas sobrepostas.

2.8.3 Análise de histologia e Microscopia Eletrônica de Varredura dos Couros de Pirarucu nos Sentido Longitudinal, Transversal e Diagonal

As fibras colágenas se apresentam em camadas paralelas a superfície do couro de pirarucu nas imagens de corte no sentido longitudinal (Figura 9). As fibras colágenas são mais finas próximo a essa superficie e vão aumentando de espessura à medida que se direcionam para o lado do carnal (hipoderme). No local onde a escama deveria estar inserida (Figura 9 B), na parte superior, encontra-se um tecido dérmico diferenciado que corresponde à lamélula de proteção, na inserção da escama. O tecido dessa lamélula é constituido de fibras bem mais finas e juntas sobrepostas em relação ao restante do couro (Figura 6, b c d).

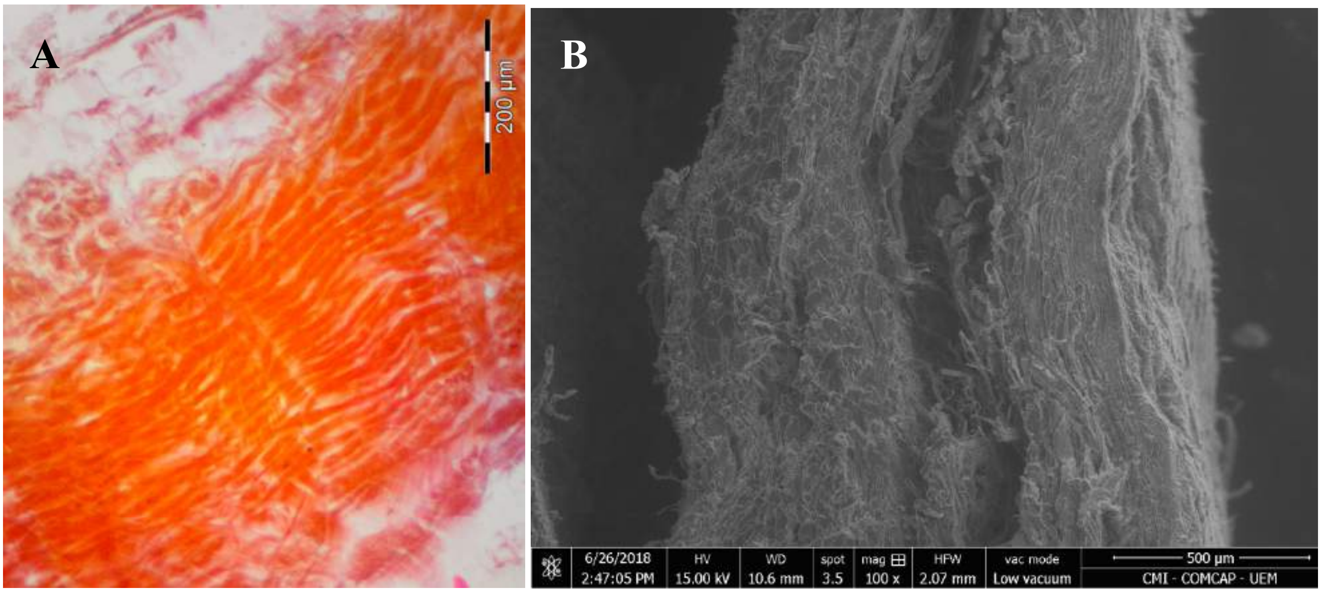

Figura 9 - Fotomicrografia (A) e Microscopia eletrônica de varredura (B) do corte do couro no sentido longitudinal ao comprimento do corpo do peixe . (A) Coloração HE.

Nas imagens do corte do couro do pirarucu no sentido transversal (Figura 10) observa-se que as fibras colágenas se encontram em camadas paralelas a superficie do couro e de espaço em espaço fibras transversais cruzam as paralelas, proporcionando uma amarração mais firme no couro. Observa-se a distribuição das fibras colágenas da lamélula mais finas e juntas e à medida que vão se afastando da superficie vão aumentando de espessura e espaçamento entre elas (figura 10).

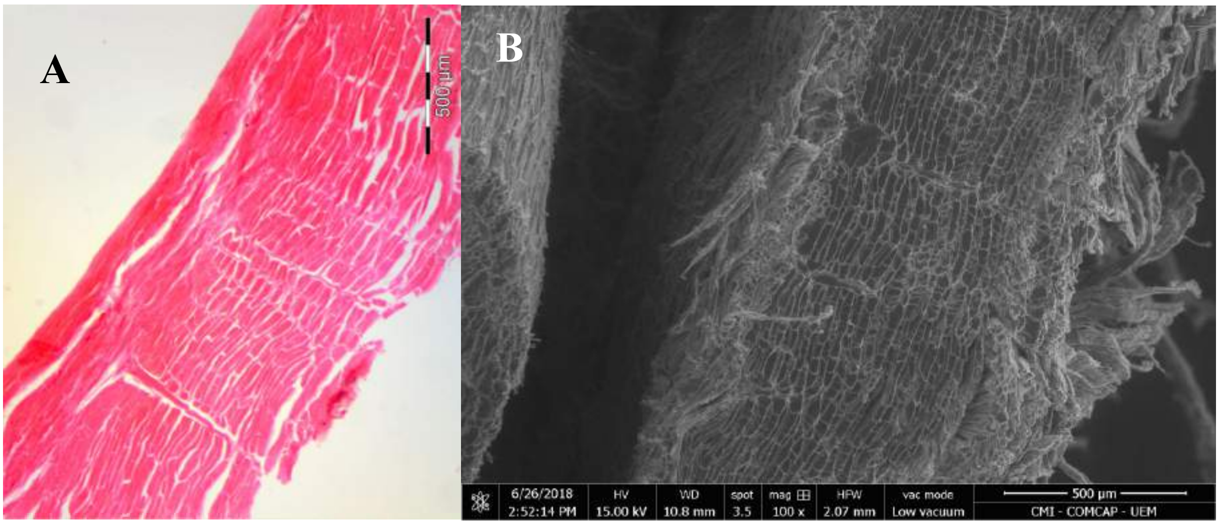

Figura 10 – Fotomicrografia (A) Microscopia eletronica de varredura (B) do corte do couro no sentido transversal ao comprimento do corpo do peixe. (A) Coloração HE.

Souza et al, (2017) avaliando o alongamento médio longitudinal ás fibras colágenas de para Cobia (*Rachycentron canadum)* obtiveram 7145% para couro tratado com sais de cromo e 81,82% quando tratadas com tanino vegetal.

Na Figura 11 pode-se observar as imagens com a distribuição dos feixes de fibras colágenas do couro de pirarucu retirada no sentido diagonal ao comprimento do corpo do animal. Na diagonal nota-se que o entrelaçamento é mais intenso, ou seja, além das camadas sobrepostas intercaladas mais finas próximo a superfície também observa-se os feixes de fibras cruzando entre si, aparentando uma rede (Figura 11A).

Junqueira et al. (1983), relataram que a derme apresenta um arranjo estrutural das fibras colágenas, permitindo que a pele possua grande resistência às diferentes forças de tração. Por essa razão, a pele de algumas espécies de peixes pode ser utilizada comercialmente na confecção de artefatos de couro.

Analisando as imagens da Microscopia eletrônica de varredura e a histologia, onde se observa a arquitetura de entrelaçamento, disposição e orientação das fibras colágenas, assim como as camadas sobrepostas dessas fibras, confirmam os resultados fisico-mecânicos obtidos dos couros curtidos nos sentidos longitudinal, transversal e diagonal (Tabela 1).

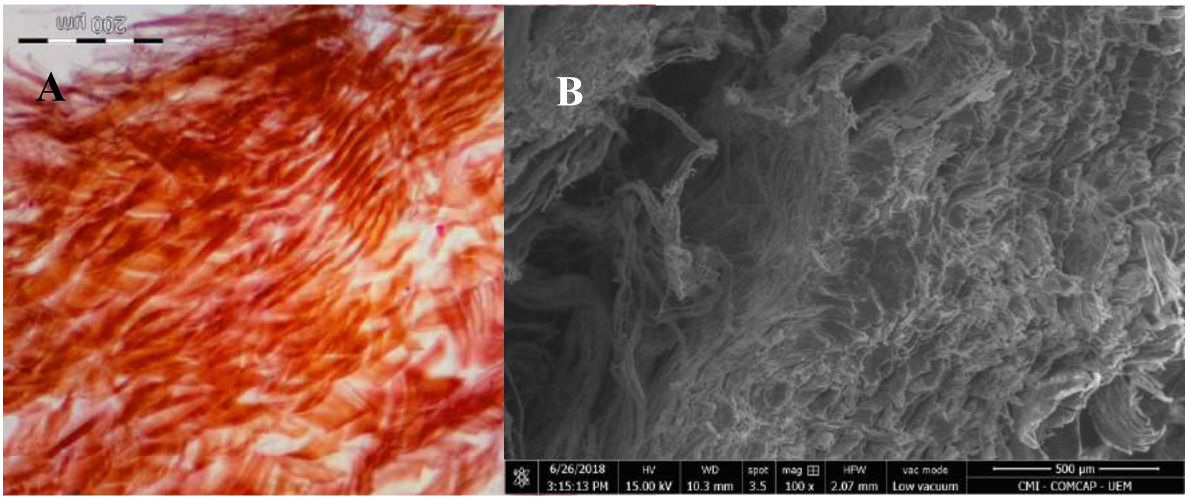

Figura 11 – Fotomicrografia (A) Microscopia eletronica de varredura (B) do corte do couro no sentido diagonal ao comprimento do corpo do peixe (A) Coloração HE.

O couro no sentido transversal e diagonal apresentaram maior tração (N/mm^2) para a ruptura do corpo de prova, ou seja das fibras colágenas do couro. Observando as imagens (Figura 10 e 11) a forma de entrelaçamento das fibras na diagonal e transversal proporcionam maior resistência a tração em função das fibras estarem distribuídas no sentido paralelo a superfície, perpendicular e cruzadas entre elas. Pelo lado carnal do couro é possível visualizar essa forma de orientação e distribuição das fibras colágenas (Figura 8 a,b). De acordo com Souza (2004) quando o couro de peixe apresenta maior quantidade de fibras de reticulina amarrando as fibras colágenas, este couro apresenta maior resistência ao rasgo. Portanto, tudo indica que no sentido longitudinal e diagonal apresentam mais fibras de reticulina amarrando aos feixes de fibras.

Greven et al. (1995) também afirmaram que a quantidade de espessas camadas de fibras colágenas tem efeito sobre as propriedades mecânicas da pele, proporcionando com isso a maior resistência ao couro quando tracionado.

2.8.4 Análises físico-química dos couros de Pirarucu

Os testes físico-químicos dos couros de pirarucu indicam que o agente curtente sais de cromo (Tabela 4) proporcionou menor teor de óxido de cromo nos couros (0,27%), inferior ao que Hoinacki (1989) indica como valor mínimo 3,0% de óxido de cromo ou acima de 2,5%, recomendação da Basf (1995) para os couros curtidos com os sais de cromo. A quantidade de

óxido de cromo dos couros está relacionada com a proporção do curtente fixado às fibras colágenas. Neste tratamento foram adicionados 5% de sais de cromo na etapa de curtimento e mais 5% na etapa de recurtimento, enquanto no tratamento com curtente vegetal, foi 15% no curtimento e o mesmo percentual no recurtimento, não sendo encontrado óxido de cromo nas amostras.

Tabela 3. Valores médios dos testes físico-químicos dos couros de Pirarucu curtidos com tanino vegetal e sais de cromo

Agente curtente	Óxido de Cromo Cr_2O_3 (%)	pH	Cifra Diferencial***	Substâncias Extraíveis com Diclorometano (%)
Tanino vegetal	0,0	3,43	0,80	21,97
Sais de cromo	0,27	3,38	0,83	16,57
Norma/Método	IT10.4-009	IT10.4-008	IT10.4-008	IT10.4-007

*** A cifra diferencial somente age como um critério para a presença de ácidos fortes livres ou bases em extrato aquoso com valores de pH abaixo de 4,0 ou acima de 10.

O pH final dos couros curtidos foi de 3,43 para o curtimento com tanino e 3,38 para sais de cromo. No entanto, o melhor pH de acordo com Hoinacki (1989) deve ser um valor mínimo de 3,5. Nessa faixa de pH ocorre melhor fixação de corante e dos óleos utilizados na etapa de engraxe (Tabela 3). Neste experimento os couros recurtidos com tanino vegetal apresentaram melhor valor de pH, mais próximo do recomendado, portanto, melhor condição para a fixação do agente curtente vegetal e os óleos utilizados no processo e maior a sifra diferencial.

Como o valor de pH ficou um pouquinho abaixo do recomendado, nota-se que a cifra diferencial, apresentou valores de 0,8 e 0,83, respectivamente para os couros curtidos com tanino vegetal e sais de cromo, sendo o recomendado no máximo 0,7 (HOINACKI ,1989). Isso mostra que houve a formação de ácidos fortes livres nas amostras analisadas. Para o tratamento com sais de cromo que o pH ficou inferior, mais ácido, o resultado da cifra diferencial foi também maior (0,83), isto porque no momento da fixação ou adição do ácido fórmico utilizado foi acima do que deveria ter adicionado, proporcionando maior quantidade de ácido livre entre as fibras colágenas do couro.

Devido ao processo de curtimento, o pH do couro normalmente apresenta-se ácido, e para curtimento com sais de cromo a acidez deve ser em torno de 3,0 e para tanino 4,0 na etapa de piquel; ao termino do processo para a fixação de corantes, agente curtente em especial o tanino vegetal e os óleos deve estar em torno de 3,5. Quando o pH estivar abaixo desse valor, significa que encontra-se uma quantidade excessiva de ácido no interior do couro podendo causar enfraquecimento do mesmo ao longo do tempo. A acidez excessiva provoca a degradação da

cadeia proteica, pela hidrólise ácida, diminuindo a resistência do couro.

Pode ocorrer redução da resistência físico-mecânica do couro, quando a quantidade de ácido for elevada (pH baixo), por apresentar poder corrosivo sobre as fibras colágenas (FLÔRES,1997). Essa acidez do couro pode proporcionar a oxidação de componentes metálicos, se colocados em contato com o couro, como rebites, fivelas e ilhoses, bem como, ocasionar alergia ou irritação para o usuário.

Para as Substâncias Extraíveis com Diclorometano o percentual foi de 21,97% para couro com tanino vegetal e 16,57% para couros com sais de cromo, ou seja, a quantidade de óleo fixada nos couros de pirarucu. Os óleos adicionados no processo de curtimento agem no deslizamento das fibras colágenas fazendo com que elas se deslizem umas sobre as outras à medida que é submetida a algum tipo de tração, proporcionando uma maior mobilidade ou elasticidade para o couro. As substâncias extraíveis em diclorometano devem estar no máximo entre 16% a 18% para o couro poder ser utilizado em vestuário (BASF, 1995). Sendo assim, apenas os couros curtidos com sais de cromo poderiam ser utilizados para a confecção de vestuário.

É interessante que a técnica utilizada proporcione maior resistência aos couros (tração, alongamento e rasgamento progressivo), com a mínima aplicação de sais de cromo ou ausência desses sais. Comparando os resultados relatados com os obtidos neste experimento com o couro de pirarucu, pode-se inferir que além dos tipos e quantidades de agentes curtentes aplicados no processo de curtimento, a espécie é fundamental na avaliação da resistência, pois está apresenta uma arquitetura na disposição e orientação das fibras colágenas que determinam uma maior ou menor resistência ao couro após processamento.

CONCLUSÃO

Os couros curtidos com tanino vegetal apresentam menor resistência que aos couros curtidos com sais de cromo para a tração até a ruptura. Os agentes curtentes não interferem na elasticidade e deformação dos couros de pirarucu, exceto quando avaliados isoladamente onde o sentido transversal do couro apresenta maior elasticidade e deformação.

REFERÊNCIAS

ASSOCIAÇÃO BRASILEIRA DE NORMAS TÉCNICAS. NBR 3377-1: Ensaios físicos e mecânicos: Determinação da força de rasgamento. Parte 1: Rasgamento de extremidade simples. Rio de Janeiro, 2014. p.1-4.

ASSOCIAÇÃO BRASILEIRA DE NORMAS TÉCNICAS NBR 11041**:** couros – determinação da resistência à tração e alongamento. Rio de Janeiro, 1997. p.1-5.

ASSOCIAÇÃO BRASILEIRA DE NORMAS TÉCNICAS. NBR 11055: couro - determinação da força de rasgamento progressivo. Rio de Janeiro, 1997. p. 1-4.

ANUSZ, L., **A arte de curtir**. Estância Velha: ABQTIC, 1995. p.100.

BAYER: CURTIR, TEÑIR, **ACABAR**, 6. ed., Faltam cidade: editora, 1997.

BASF S.A**. Vade-mécum do Curtidor**. 5ªedição, Ludwigshafen: BASF, 2005.

CARTILHA DO COURO. CTCCA - Centro tecnológico do couro, calçado e afins.p.18-20, 2000.

DANIELS, R., **Raw Material and Preservation**, World Leather, Part 2: May, p. 52 e 53, April/May 2002.

GREVEN, H.; ZAMGER, K.; SCHWINGER, G. Mechanical proprieties of the Xenoplus laevis (Anura, Amphibia)**. Journal Morphology**, v.224, p.15-22, 1995.

GUTTERRES, M. **Estrutura de Colágeno na Pele**. Revista do Couro Abqtic, Estância Velha, n. 170,2004.

HOINACKI, E. GUTHIEL,N.C. **Peles e Couros: origens, defeitos e industrialização**, 1 edição, SENAI/RS, Porto Alegre, 1979, p.19.

HOINACKI, E.; MOREIRA, M.V.; KIEFER, C.G. **Manual básico de processamento do couro**. SENAI, Centro Tecnológico do Couro, 1994.402p.

MK Equipe Técnica, **Controle de Qualidade em Couros** Wet-blue, MK News, Ano 6, n. 26, p. 11 – 15, Julho de 2003.

MK Equipe Técnica, **Manual de aplicação para produção de couro**, p.10, Março de 1999.
PRIBE, G.P.S. 2005.

SOUZA, M.L.R. **Tecnologia para processamento das peles de peixe**. Maringá: Eduem, Coleção Fundamentum nº 11.2004. 59p.

SOUZA, M. L. R, GASPARINO, E. PENHA, B.G., CORADINI, M.F. GOES, E.S.R, GONÇALVES, A.A. Physicochemical and mechanical characteristics of cobia (*Rachycentron canadum*, Linnaeus, 1766) leather submitted to different tanning agents in the retanning step. **International Journal of Latest Research in Science and Technology**. v.6, n.2:p.8-13, March-April 2017.

SOUZA, M.L.R.; GANECO, L.N.; NAKAGHI, L.S.O.; FARIA, R.H.S.; WAGNER, P.M.; POYH, J.A. E FERREIRA, I.C. Histologia da pele do pacu (*Piaractus mesopotamicus*) e testes de resistência do couro. **Acta Scien Animal Science**, 25: 37-44. 2003.

VEIGA, M.C.M., MELO JR C.A.F., SANTOS, G, VIDAL, J.M.A., COSTA W.M. Extração de tanino vegetal do eucalipto no curtimento de pele de peixe: aspectos fitogeográficos. In: JORNADA DE ENSINO, PESQUISA E EXTENSÃO – JEPEX. 8, 2013 – UFRPE: Recife. **Anais...** Recife, 2013.

CAPITULO III

3 TANINO VEGETAL E EXTRATO DE URUCUM EM SUBSTITUIÇÃO AOS PRODUTOS QUÍMICOS NO CURTIMENTO E TINGIMENTO DO COURO DO PIRARUCU

RESUMO

Nas últimas décadas, os problemas ambientais têm se tornado cada vez mais críticos e freqüentes, devido ao aumento da atividade industrial. No setor dos curtumes não é diferente. A cada dia buscam novas alternativas para se diminuir os impactos ambientais causados pelos tingimentos, que é uma das etapas do processo de curtimento. Pensando nisso, o objetivo deste trabalho foi avaliar a substituição de corantes químicos por corante natural (urucum) em couros curtidos com sais de cromo ou tanino vegetal. O Estudo foi realizado pela Universidade Federal de Rondônia e o Curtimento das peles no Curtume Texturas da Amazônia, em março de 2017, de localizado em Ji-paraná, Rondônia, Brasil. A pesquisa atendeu aos requisitos no Comitê de ética ao Uso de Animais - CEUA número 031/2018. Para o processo de curtimento e coloração foram utilizadas 42 ½ peles com média de peso in natura de 1,0 ± 0,40 kg de 21 Pirarucus de 12 ± 0,5 kg, distribuídas em delineamento inteiramente casualizado em fatorial 2 x 3 sendo, 2 curtentes (sais de cromo e tanino vegetal, Acacia mearnsii) e 2 corantes (químico e urucum), com 7 repetições para cada tratamento. Foram utilizados 4% de corante químico sobre o peso dos couros para realizar o tingimento e para a fixação do corante 2 % ácido fórmico. Para o tingimento com corante natural foi utilizado 6% de urucum. A extração do urucum foi feita de forma artesanal. Foram separados 10 kg de urucum e deixados em um recipiente com água e 2% de etanol sob o peso do urucum. Após cinco dias de fermentação, o urucum foi separado o líquido dos grãos e armazenado em um balde com tampa para ser utilizado no momento do tingimento dos couros. Foi realizado a leitura em 3 pontos aleatório na superfície do couro de pirarucu e 3 repetições, sendo o couro a unidade experimental. A leitura foi realizada no aparelho Modelo MiniScan EZ, brand Hunter Lab, com fonte de luz D65, ângulo de observação 10º, abertura das células de medição de 30 mm, utilizando a escala L*,a*, b* do Sistema Cielab (JUSS e HUNTER, 1975). Sendo o valor L * representa o brilho, indica leveza (L * = 0 corresponde a preto e L * = 100 a branco), o a* e b* representam a saturação (croma ou pureza) e matiz (cor). Os valores de a* variando de valores de − a* (verde) até + a* (vermelho) e b* varia de −b* (azul) para + b* (amarelo). As analises referente a colorimetria quanto a resistência

da cor dos couros conjugados a diferentes tecidos/materiais ás intempéries (envelhecimento acelerado, solidez a luz). A combinação tanino vegetal e urucum preservou a integridade das fibras colágenas com maior espessura do couro (2,10mm vs. 2.61mm) e demandando mais força (178,2N vs. 125,3N), para danos físico-mecânicos. As médias para a força de tensão á tração foi de 6,38N/mm^2, rasgamento 30,3mm, alongamento 50,6% e rasgamento de 40,0N/mm (P>0,05). luz UV, a agua e ao suor) foram realizada no Laboratório Leather Solutions, RS. O efeito dos corantes pode ser potencializado pelo curtente utilizado, porém, o tingimento com urucum reduz o envelhecimento acelerado do couro e dá maior eficácia quanto a solidez da cor ao suor e demais intempéries, independente do tecido, mesmo quando precedido por curtente químico, características que dão maior maleabilidade e resistência na produção de produtos de confecções e artefatos com couro de Pirarucu.

Palavras-chaves: cores. coloração. envelhecimento acelerado. couro tingido.

ABSTRACT

In the last decades, environmental problems have become increasingly critical and frequent due to the increase in industrial activity. In the tannery sector it is no different. Every day they seek new alternatives to reduce the environmental impacts caused by dyeing, which is one of the stages of the tanning process. The objective of this study was to evaluate the substitution of natural dyes (urucum) in hides tanned with chromium salts or vegetable tannins. The study was carried out by the Federal University of Rondônia and the tanning of the skins in Curtume Textures of the Amazon, in March 2017, located in Ji-paraná, Rondônia, Brazil. The research met the requirements in the Ethics Committee on the Use of Animals - CEUA number 031/2018. For the tanning and coloring process, 42 ½ skins with a mean in natura weight of 1.0 ± 0.40 kg of 21 Pirarucus of 12 ± 0,5 kg were used, distributed in a completely randomized design in factorial 2 x 3, 2 tannins (salts of chromium and vegetable tannin, Acacia mearnsii) and 2 dyes (chemical and urucum), with 7 replicates for each treatment. 4% of chemical dye was used on the weight of the hides to dye and to fix the dye 2% formic acid. For the dyeing with natural dye was used 6% of urucum. The extraction of the annatto was done in an artisan way. 10 kg of urucum were separated and left in a container with water and 2% ethanol under the weight of urucum. After five days of fermentation, the annatto was separated the liquid from the beans and stored in a bucket with lid to be used at the time of the dyeing of the leathers. It was carried out the reading in 3 random points in the surface of the pirarucu leather and 3 repetitions, being the leather the experimental unit. The reading was performed on the Model MiniScan EZ, Hunter Lab brand, with light source D65, viewing angle 10º, opening of the measuring cells 30 mm, using the L *, a *, b * scale of the Cielab System (JUSS and HUNTER, 1975). As the value L * represents the brightness, it indicates lightness (L * = 0 corresponds to black and L * = 100 a white), a * and b * represent saturation (chroma or purity) and hue (color). The values of a * varying from -a * (green) to + a * (red) and b * ranges from -b * (blue) to + b * (yellow). The analyzes concerning colorimetry as to the color resistance of the leather conjugated to different fabrics / materials in the weather (accelerated aging, light fastness). The tannin and urucum combination preserved the integrity of collagen fibers with greater thickness of leather (2.10mm vs. 2.61mm) and requiring more strength (178.2N vs. 125.3N) for physical-mechanical damages. The averages for tensile tensile strength were 6.38N / mm², tear 30.3mm, elongation 50.6% and tear 40.0N / mm (P> 0.05). UV light, water and sweat) were performed at the Laboratory Leather Solutions, RS. The effect of the dyes can be enhanced by the curing agent used, however, dyeing with urucum reduces the accelerated aging

of the leather and gives greater effectiveness as the color fastness to sweat and other weather, regardless of the fabric, even when preceded by chemical tanning, characteristics which give greater malleability and resistance in the production of garments and artifacts with Pirarucu leather.

Key words: colors. coloring. accelerated aging. dyed leather.

3.1 Introdução

O Brasil tem se destacado, nos últimos anos, com um crescimento bastante expressivo na aquicultura, onde aumentou sua produção em 123% entre 2005 e 2015, segundo a Embrapa Pesca e Aquicultura.

O estado de Rondônia, em 2016, alcançou a marca das 90 mil toneladas de peixes (IBGE, 2016) se tornando o maior produtor de peixes em água doce de cativeiros. Essa produção gera uma imensa quantidade de resíduos sólidos onde há pouca destinação e reutilização desses subprodutos. Rondônia visa duplicar essa produção pesqueira onde há a necessidade de desenvolvimento de estudos visando à reutilização dos resíduos gerados por essa cadeia industrial que está se instalando na região de modo a trazer mais sustentabilidade e responsabilidade ambiental a essa fatia do PIB estadual.

Dentro do aspecto sustentável de desenvolvimento tem se dado destaque, principalmente, aos impactos ao meio ambiente, inserindo conceitos e normas legais a fim de proteger e desenvolver formas eficientes de exploração destes meios, que são as fontes primárias de todo processo industrial desenvolvido pelo homem.

A exploração do couro do Pirarucu, na indústria têxtil e artefatos, tem crescido em função de suas especificidades comparado a pele das demais espécies de pescado e ao couro bovino. Contudo, os processos para a produção do couro têm utilizado os procedimentos padrões de curtumes de couro bovino.

Os curtumes têm sido categorizados como grandes causadores de impacto ambiental, devido à poluição gerada por seus resíduos. Os corantes químicos quando não tratados podem trazer prejuízos à fauna, a flora e até ao homem além da poluição visual e alterações no ecossistema. No Brasil a produção de corantes tem ocorrido desde seu descobrimento, relacionada à madeira Pau Brasil (*Caesalpinia echinata*, Lam.), da qual era extraído um pigmento capaz de tingir tecidos com cores fortes, como vermelho, rosa ou marrom (DALLOGO & SMANIOTTO, 2005).

Os corantes químicos tem expressiva relevância no tingimento de couros para indústria têxtil, porém o uso de corantes vegetais pode vir como alternativa aos processos comuns, aproveitando recursos naturais e reduzindo custo de produção, ajustando-se a menores impactos ambientais.

Os pigmentos naturais são normalmente agrupados, em função de sua estrutura química (FERREIRA, 1998). O extrato de açaí possui propriedade tintórias que além de sua cor intensa, é um resíduo natural e abundante da extração vegetal nativo na região

amazônica, a qual possui ainda uma diversidade de cascas, sementes, frutos e folhas que podem ser utilizados como corantes naturais. No Brasil, uma das principais matérias-primas utilizadas na produção de corantes naturais é o urucum, originário da América Tropical, pertence à família Bixaceae (GOUVEIA; MOURA; MEDEIROS, 2000) com pigmento extraído da camada externa das sementes da planta de *Bixa orellana L.* sendo fonte do carotenoide bixina (STRINGHETA; SILVA, 2008).

O processo de curtimento pode ser antecedido por uma etapa de pré-curtimento, ou condicionamento, e este é sempre empregado para o caso de curtimento vegetal. Este processo visa preparar a pele para deixá-la mais suscetível à difusão e a fixação dos curtentes, evitando que o curtimento ocorra apenas externamente em sua superfície (HOINACKI; MOREIRA; KIEFER, 1994). Com relação ao peixe, o seu tamanho está diretamente relacionado com a espessura da pele, pois à medida que o peixe vai crescendo ocorre um aumente da espessura e, consequentemente, da quantidade de fibras colágenas. Os parâmetros que indicam a tração (carga de força, tensão de tração e elasticidade) podem ser correlacionados com a quantidade e a orientação das fibras colágenas. Portanto a espessura da derme é determinada principalmente, pela proporção das fibras colágenas na pele, fibras estas que reagirão com os agentes curtentes, dando a característica de resistência ao couro (SOUZA, 2003).

Segundo Filho (2006) a resistência à flexão é uma propriedade importante a considerar na indústria do couro, pois todos artefatos acabam sofrendo “dobras” em suas manipulações quando no produto acabado, tais como calçados, bolsas, malas, casacos, cintos, etc. Com a resistência a flexão é possível medir a maciez do couro. A maciez está profundamente ligada ao tipo de pele utilizado e aos processos do curtimento e engraxe (RITTERBUSCH et al., 2014).

Segundo Calciolari et al. (2013), as vantagens existentes no emprego do tanino vegetal são: melhorias no tratamento de efluentes, resíduos mais biodegradáveis, utilização dos resíduos na agricultura, água residuária e lodo livre de cromo, além de pó de serragem provenientes do lixamento e rebaixamento também isentos de cromo.

Entre as espécies de peixes atualmente cultivadas, a pele da tilápia é a que está ganha mais espaço no processo do curtimento, para seu aproveitamento no beneficiamento de artefatos e vestuários. Entretanto, para melhor aproveitamento da pele na indústria coureira, deve-se considerar a espécie, tamanho, qualidade de pele, além de sua beleza, principalmente para os peixes que apresentam escamas. O desenho característico formado pelas lamélulas de proteção da inserção dessas escamas resulta em um aspecto típico e difícil de ser imitado

e garante um padrão exclusivo de alto impacto visual (SOUZA et al, 2002; 2003; 2004). Segundo Ingram e Dixon (1994), as peles de peixes são consideradas como um couro exótico e inovador, com aceitação geral em vários segmentos da confecção.

Com foco nessas ideias, muitos trabalhos científicos foram e estão sendo desenvolvidos para, não somente demonstrar os impactos causados pela ação produtiva do homem, mas também pelas técnicas e métodos de produção que amenizam ou anulem essas interferências no equilíbrio natural do meio ambiente. Atentos a essas questões o projeto visa à sustentabilidade substituindo aditivos químicos (corantes e curtentes) por extratos vegetais naturais com a mesma capacidade qualitativa na produção final do couro, utilizando fontes da flora amazônica e pampa em substituição ao método comum de curtimento de peles utilizando sais de cromo, e a utilização dos resíduos da indústria da piscicultura, avaliando concentrações dessas fontes naturais na qualidade dos processos de fabricação do couro de pirarucu.

Objetivou-se avaliar os aspectos físico-mecânicos, histológicos e colorimétricos do couro de Pirarucu submetido ao curtimento e tingimento por produtos naturais em substituição a produtos químicos.

3.2 Objetivo Geral

Avaliar a substituição de corantes químicos por corante natural (urucum) em couros curtidos com sais de cromo ou tanino vegetal

3.3 Objetivos Específicos

Comparar o efeito do corante natural e químico em couros curtidos com sais de cromo e tanino vegetal;

Testar o Urucum como corante natural em substituição a corantes químicos.

Verificar se os corantes influenciaram na resistência dos couros

3.4 Material e Métodos

O Estudo foi realizado pela Universidade Federal de Rondônia e o Curtimento das peles no Curtume Texturas da Amazônia, em março de 2017, de localizado em Ji-paraná, Rondônia, Brasil. A pesquisa atendeu aos requisitos no Comitê de ética ao Uso de Animais - CEUA número 031/2018.

Para o processo de curtimento e coloração foram utilizadas 42 ½ peles com média de peso *in natura* de 1,0 ± 0,40 kg de 21 Pirarucus de 12 ± 0,5 kg, distribuídas em delineamento inteiramente casualizado em fatorial 2 x 3 sendo, 2 curtentes (sais de cromo e tanino vegetal, *Acacia mearnsii*) e 2 corantes (químico e urucum), com 7 repetições para cada tratamento.

3.4.1 Curtimento

Ao final do processo de curtimento, os couros foram estabilizados em 18% de umidade, mensurando-se a área, peso e espessura da pele através de paquímetro para posteriores análises.

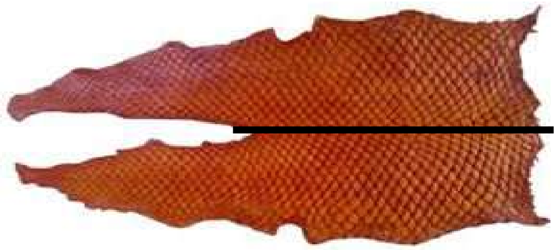

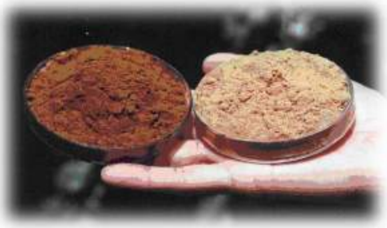

Figura 12. ½ pele de Pirarucu (A) distribuídas nos curtentes tanino vegetal e sais de cromo

3.4.2 Tingimento

Foram utilizados 4% de corante químico sobre o peso dos couros para realizar o tingimento e para a fixação do corante 2 % ácido fórmico. Para o tingimento com corante natural foi utilizado 6% de urucum. O tempo da etapa de tingimento foi de 2 horas em fulão.

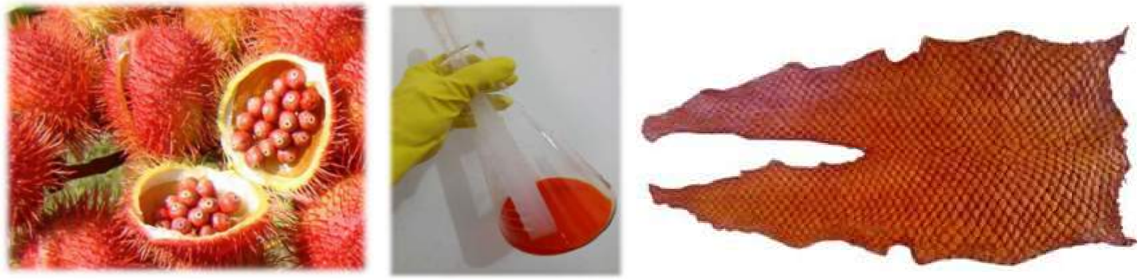

Figura 13. Corante Urucum (*bixa orellana*) extraído e utilizado no tingimento da pele do Pirarucu.

Após os processos de tingimento dos couros, as amostras foram enviadas ao Laboratório Leather Solutions, no Rio Grande do Sul.

3.4.2.1 urucum

O urucuzeiro é originário da América Tropical, pertence à família Bixaceae com o nome botânico de Bixa orellana L. (GOUVEIA; MOURA; MEDEIROS, 2000). Dependendo da região de cultivo e da idade da planta, apresenta-se como um arbusto perene grande ou como uma árvore pequena, variando de 2 a 5 metros de altura. A planta exibe grande variabilidade de coloração, com caule, frutos verdes e flores brancas ou caule vermelho, flores rosas e frutos vermelho-escuro. Os grãos são arredondados, revestidos por uma camada pastosa de coloração avermelhada, os quais tornam-se secos, duros e de coloração escura com o amadurecimento. O extrato de urucum tem uma estabilidade considerável à oxidação pelo oxigênio em meio anidro, mas uma resistência mais baixa aos efeitos da luminosidade. Uma forma de garantir a estabilidade do corante sob a incidência luminosa é a adição de antioxidantes, conforme sugeriram Najar, Bobbio e Bobbio (1988). Kiokias e Gordon (2003).

Os grãos de urucum ainda possuem outros componentes de importância, mas pouco explorados, como o geranilgeraniol, que apresenta importantes propriedades farmacológicas.

3.4.2.2 extração do urucum

A extração do urucum foi feita de forma artesanal. Foram separados 10 kg de urucum e deixados em um recipiente com água e 2% de etanol sob o peso do urucum. Após cinco

dias de fermentação, o urucum foi separado o líquido dos grãos e armazenado em um balde com tampa para ser utilizado no momento do tingimento dos couros.

3.4.2.3 análises de colorimetria dos couros pelo sistema CIELAB

Foi realizado a leitura em 3 pontos aleatório na superfície do couro de pirarucu e 3 repetições, sendo o couro a unidade experimental. A leitura foi realizada no aparelho Modelo MiniScan EZ, brand Hunter Lab, com fonte de luz D65, ângulo de observação 10º, abertura das células de medição de 30 mm, utilizando a escala L*,a*, b* do Sistema Cielab (JUSS e HUNTER, 1975**).** Sendo o valor L * representa o brilho, indica leveza (L * = 0 corresponde a preto e L * = 100 a branco), o a* e b* representam a saturação (croma ou pureza) e matiz (cor). Os valores de a* variando de valores de − a* (verde) até + a* (vermelho) e b* varia de −b* (azul) para + b* (amarelo). A calibração do instrumento foi realizada antes de ler as amostras com o padrão branco e um preto.

3.4.2.4 análises de colorimétrica pelo uso do couro

As analises referente a colorimetria quanto a resistência da cor dos couros conjugados a diferentes tecidos/materiais ás intempéries (envelhecimento acelerado, solidez a luz UV, a agua e ao suor) foram realizada no Laboratório Leather Solutions, RS.

Os parâmetros colorimétricos para as alterações na cor da flor do couro do Pirarucu foram avaliados quanto ao envelhecimento acelerado e solidez a luz UV, por 72 horas a 100°C, e quanto ao manchamento do tecido, quando submetido à solidez da cor à agua e solidez da cor ao suor nos tecidos cotton, poliamida, poliéster, acetato, acrílico e lã.

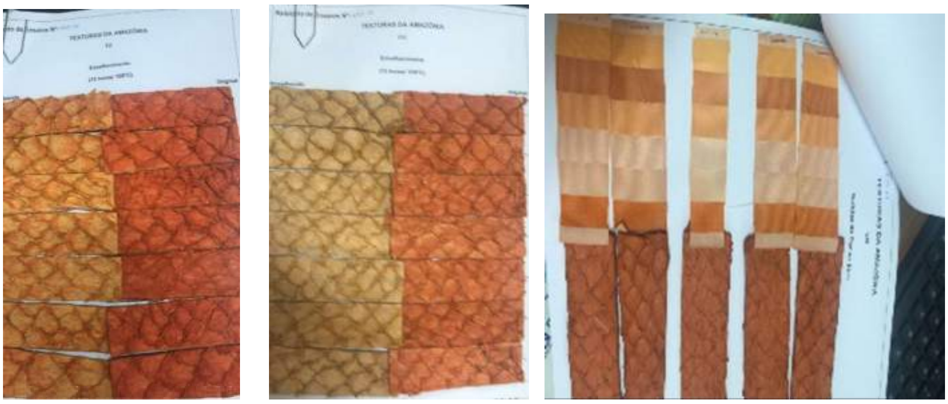

Figura 14. Corpos de prova de couro de Pirarucu submetidos a curtentes e corantes químicos e naturais utilizados nos testes de colorimetria.

3.4.2.5 testes de resistência dos couros

Após curtimento, os couros secos foram encaminhados a um laboratório aclimatizado (23ºC e 50% de umidade relativa do ar) por 24 horas, conforme ABNT (NBR ISSO 4044, 2015). Foram retirados os corpos de prova por tratamento com auxilio de balancim (ABNT– NBR, 11035, 2015a), e com um espessímetro foi determinada as espessuras de cada amostra (ABNT – NBR ISSO 2418, 2015). Os corpos de prova foram retirados da região dorsal do couro do pirarucu curtidos pelos dois agentes curtentes e dois tipos de corantes (urucum e químico) no sentido longitudinal, transversal e diagonal ao comprimento do corpo do peixe, sendo utilizado o valor médio dos sentidos (Figura 1).

Foi determinada a resistência à tração e ao alongamento (ABNT – NBR ISSO 3376, 2014) e ao rasgamento progressivo (ABNT – NBR 11055, 2014). Para os testes de resistência foram avaliadas a força (N) aplicada no teste, a tração (N/mm^2) até a ruptura do couro e para avaliação da elasticidade destes couros o alongamento (%) e deformação ou maciez (mm), que ocorreu ao ser tracionado. Para a determinação do rasgamento progressivo (N/mm) foi avaliado a força máxima (N) e média (N) aplicada no teste.

Para os testes de resistência, foi utilizado dinamômetro da marca EMIC, com velocidade de afastamento entre cargas de 100 ± 20 mm/mm. Foi utilizada uma célula de carga de 200 kgf e a calibração foi realizada pela Emic-dcame, laboratório de calibração credenciado pela CGCRE/inmetro sob nº 197

As análises de determinação de resistência dos couros foram realizadas pelo Laboratório de Processamento de peles e couros da Universidade Estadual de Maringá.

3.4.2.6 análises físico-química dos couros de Pirarucu

A preparação de amostras de couro para análise química seguiu as condições exigidas pelas normas da ABNT (NBR ISSO 4044, 2015), para a determinação do óxido de cromo Cr_2O_3 (ABNT – NBR' 10054, 2014), determinação das substâncias extraíveis com diclorometano (CH_2Cl_2) (ABNT - NBR 11030, 2013 e a determinação do pH e da cifra diferencial do pH de um extrato aquoso (ABNT- NBR 10455, 2014).

3.4.3 Análise Estatística

Os dados das variáveis analisadas foram apresentados como média ± desvio padrão para cada tratamento testado. Para análise dos dados foi utilizado ANOVA seguido de teste de comparações múltiplas (Tukey 5%). Para todas as análises foi utilizado o programa SAS Inst. Inc., Cary, NC, USA. (SAS, 2010).

3.5 Resultados e discussões

3.5.1 Testes de Resistência em Couros de Pirarucu Curtidos com Tanino Vegetal e Sais de Cromo e Submetidos ao Tingimento com Corante Natural e Químico

Os couros curtidos com tanino vegetal (média de 2,56 a 2,60mm) apresentaram significativamente maior espessura que os couros curtidos com sais de cromo (média 2,06 a 2,15 mm) (Tabelas 1 e 2).

A força máxima aplicada no teste de tração e alongamento variou de 85,57N a 178,28N. Houve interação do agente curtente com os tipos de corantes utilizados na etapa de tingimento dos couros. Com o desdobramento da interação, os couros curtidos com tanino e tingidos com corante vegetal (urucum) necessitou aplicar uma maior força para a ruptura do corpo de prova a tração, quando comparado aos curtidos com tanino e corante químico e sais de cromo com corante natural.

Os agentes curtentes e corantes utilizados influenciaram no resultado da tração a ruptura, cujos valores variaram entre os tratamentos de 4,92 a 7,38 N/mm^2. Fazendo o desdobramento da interação, nota-se que os couros curtidos com sais de cromo e tingidos com corante químico (7,38 N/mm^2) apresentaram maior resistência à tração, porém não diferiu significativamente dos couros curtidos com tanino vegetal, independente do corante utilizado no tingimento (corante químico 6,47 N/mm^2 e urucum = 6,75N/mm^2) (Tabela 1).

Quanto a elasticidade (%) do couro e a deformação ou maciez ocorrida em milímetros, somente houve influencia da ação dos corantes, ou seja, foi significativamente maior para os couros tingidos com corante químico em relação ao corante vegetal (urucum). O processo de curtimento utilizado com tanino e com sais de cromo proporcionaram uma elasticidade média de 50,61% e o aumento em centímetros no comprimento do corpo de prova foi de 30,35 mm, ou seja, de 3,04 cm.

Tabela 1. Valores médios da avaliação físico-mecânica dos couros de Pirarucu curtidos com tanino vegetal e sais de cromo e submetidos ao tingimento com corante vegetal (urucum) e químico.

Couro		Espessura (mm)	Força (N)	Tração (N/mm^2)	Deformação (mm)	Alongamento (%)
Tanino	Urucum	2,67±0,38	178,28±43,14a[1]	6,75±1,86ab	28,57±5,33	47,61±8,82
	Químico	2,52±0,60	134,33±91,56b	6,47±2,89ab	31,95±10,06	53,14±16,76
Cromo	Urucum	2,04±0,39	85,57±43,05c	4,92±2,18b	27,38±7,26	45,80±11,95
	Químico	2,27±0,42	156,28±88,19ab	7,38±3,65a	33,52±8,09	55,90±13,64
			Efeitos principais			
Curtente	Tanino	2,60±0,51a	156,30±74,11	6,61±2,40	30,26±8,13	50,38±13,52
	Cromo	2,15±0,41b	120,92±77,32	6,15±3,22	30,45±8,21	50,85±13,66
Corante	Urucum	2,36±0,49	131,92±63,35	5,84±2,21	27,97±6,32b	46,71±10,41b
	Químico	2,39±0,53	145,30±89,48	6,92±3,28	32,73±9,05a	54,52±15,16a
			Valor de P			
Curtente (C)		0,0001	0,0241	0,4404	0,9120	0,8693
Corante (C)		0,7097	0,3870	0,0720	0,0070	0,0078
C x C		0,0675	0,0004	0,0298	0,4242	0,4270
C.V.[2] (%)		19,39	50,85	42,28	25,94	25,91

*médias ± desvio padrão seguidas de mesma letra minúscula na coluna não diferem ao teste de tukey a 5% de probabilidade;

Os agentes curtentes utilizados no processo de curtimento e os corantes empregados na etapa de tingimento não influenciaram na força máxima e média aplicada no teste de determinação do rasgamento progressivo (Tabela 2). Os valores variaram de 83,14N a 99,23N e de 66,04N a 79,95N, respectivamente para a força máxima e força média. Todavia, para o rasgo houve interação entre os agentes curtentes e os corantes utilizados, sendo o uso do curtente vegetal e corante químico proporcionou a menor resistência ao rasgamento progressivo (35,51 N/mm), diferindo significativamente apenas dos couros curtidos com sais de cromo e tingidos com corante químico.

A resposta dos corantes está diretamente relacionada ao curtente que precede o processamento do couro. Nos couros submetidos ao curtimento pelo tanino vegetal os corantes responderam de forma semelhante ($P<0,05$) quanto a espessura da pele e a força aplicada para as respostas físico-mecânicas, porém o urucum (2,67mm e 178,28 N) foi superior em 5,6% e 24,6% ao corante químico (2,52mm e 134,3 N), respectivamente (Tabela 1).

Uma explicação sobre esse processo é que o corante natural (urucum) influenciou na ligação dos sais de cromo com a camada peptídica do couro, o que atribui maior resistência por estar diretamente ligada à resistência do colágeno (GUTIERRES, 2004) e a quantidade dessas fibras. Já, quando houve a troca do curtente químico pelo curtente vegetal (tanino), mesmo sequenciado ao corante químico, mostrou melhor resultado nos diversos testes de resistência do couro.

Os sais básicos de cromo são capazes de formar macromoléculas que se ligam à cadeia polipeptídica do colágeno (Figura 1B), já o tanino depende primariamente da hidroxila fenólica (OH) ligar-se à camada peptídica o que atribui maior resistência ao couro (Figura 1A).

Figura 1. Ligações cruzadas com os produtos curtentes: (a) tanino vegetal, (b e c) cromo.

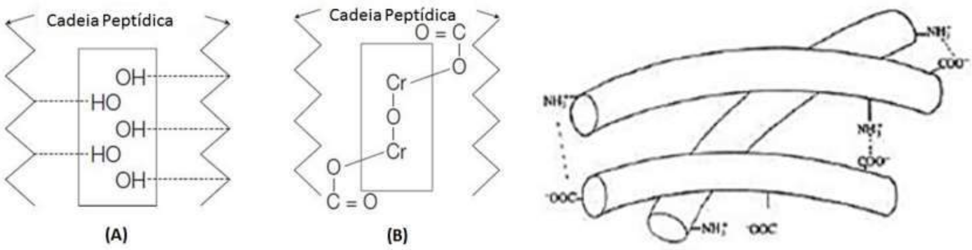

As etapas de difusão e fixação do curtente estão intimamente ligadas a basicidade do curtente. Quanto maior a basicidade maior a fixação nas camadas externas do couro e maior o poder curtente. Comumente usa-se curtentes de cromo com basicidade de 33% ou pH a baixo de 3, para garantir a penetração do cromo até atingir a basicidade. No entanto, diminui-se a difusão para as camadas mais internas, podendo estas ser curtidas posteriormente ao aumentar

a basicidade entre 45 a 50% através da adição de compostos alcalinos, como bicarbonato de sódio e o óxido de magnésio, a fim de permitir que os complexos de cromo possam penetrar na matriz colágena através da reação do complexo de cromo com proteína, na qual o cromo se liga diretamente ao grupo carboxílico do colágeno (FUCK; GUTIERRES; MARCILIO, 2007).

Para o rasgamento do couro, a força aplicada foi numericamente superior (99,2N vs. 84,1N) para os couros submetidos ao curtente e corante vegetais (tanino e urucum), comparado a média dos demais tratamentos, apesar de não haver diferenças (P>0,05) entre a espessura do couro quando tingidos com corante químico ou vegetal (P<0.05) (Tabela 2).

Os dados de rasgamento apresentam um couro com superior resistência, homogêneo vegetal em face do tratamento heterogêneo, mostrando-se adequados ao uso na confecção de vestuários, seguindo padrões adotados por Hoinacki (1989), com valores maiores que 9,8 N/mm^2 e aplicação de 60% para força, tração e alongamento, respectivamente.

Pressupõem-se três tipos de ligações: Enlaces eletrostáticos ou salinos grupos amino livres da proteína e os grupos sulfônico dos corantes; Pontes de Hidrogênio ativos do corante e os centros de alta densidade eletrônica sobre a proteína ou entre os hidrogênios ativos do couro e enlaces azo do corante; Forças de Van der Waals entre corantes proteína; Enlaces covalentes entre corante e complexo de cromo (FUCK, GUTERRES, MARCILIO, 2007). Quanto menor o pH, mais superficial a fixação de corantes aniônicos e mais forte, as temperaturas mais baixas promovem penetração mais profunda, já quando a temperatura é elevada a fixação é mais superficial (FUCK, GUTERRES, MARCILIO, 2007). Para tanto, a eficiência do corante utilizado depende das reações precedentes ocorridas no curtimento do couro, pois estão diretamente relacionadas a basicidade e tipo de ligações para com as fibras colágenas.

O tanino vegetal proporciona um couro 24% mais espesso; 34,78% mais resistente à aplicação de força; 3,21% mais resistente a tensão à tração e 10,4% mais elástico.

A combinação tanino vegetal e urucum preservou a integridade das fibras colágenas com maior espessura do couro (2,10mm vs. 2.61mm) e demandando mais força (178,2N vs. 125,3N), para danos físico-mecânicos. As médias para a força de tensão á tração foi de 6,38N/mm^2, rasgamento 30,3mm, alongamento 50,6% e rasgamento de 40,0N/mm (P>0,05).

Tabela 2. Rasgamento progressivo dos couros de Pirarucu curtidos com tanino vegetal e sais de cromo e submetidos ao tingimento com corante vegetal (urucum) e químico.

	Couro	Espessura (mm)	Rasgo (N/mm)	Força média (N)	Força máxima (N)
Tanino	Urucum	2,60±0,53	40,81±10,28ab	79,95±23,20	99,23±33,57
	Químico	2,52±067	35,51±10,79b	66,66±19,55	88,42±23,83
Cromo	Urucum	2,20±0,26	38,26±9,45ab	67,27±14,75	83,14±17,62
	Químico	1,93±0,46	45,52±13,35a	66,04±19,59	84,66±22,64
		Efeitos principais			
Curtente	Tanino	2,56±0,60a	38,16±10,75	73,30±22,23	93,83±29,27
	Cromo	2,06±0,39b	41,89±12,00	66,64±16,92	83,90±20,05
Corante	Urucum	2,40±0,46	39,54±9,84	73,59±20,25	91,19±27,70
	Químico	2,22±0,64	40,52±13,01	66,35±19,13	86,54±23,04
		Valor de P			
Curtente (C)		0,0001	0,1268	0,1194	0,0736
Corante (C)		0,1176	0,6860	0,0913	0,3990
C x C		0,4080	0,0110	0,1572	0,02635
C.V.² (%)		21,86	27,64	27,75	28,23

*médias ± desvio padrão seguidas de mesma letra minúscula na coluna não diferem ao teste de tukey a 5% de probabilidade;

3.5.2 Testes de Físico-Químicos em Couros de Pirarucu Curtidos com Tanino Vegetal e Sais de Cromo e Submetidos ao Tingimento com Corante Químico e Natural

Os valores dos testes físico-químicos dos couros de pirarucu constam na Tabela 3. Os couros curtidos com sais de cromo apresentaram baixo teor de óxido de cromo, sendo inferior a 0,49%. O recomendado seria o valor mínimo de 3,0% de óxido de cromo segundo Hoinacki (1989) ou acima de 2,5% de acordo com Basf (1995). Atualmente o recomendado é um percentual maior devendo ser ≥3,5%, para cabedal de moda (ABNT NBR 13525, 2016). Essa quantidade de óxido de cromo fixada no couro está relacionada com a proporção de agente curtente adicionado no processo de curtimento e o ligado às fibras colágenas. Neste tratamento foram adicionados 5% de sais de cromo nas etapas de curtimento e de recurtimento, mas provavelmente a fixação desses sais não foi eficiente.

O pH final dos couros curtidos neste experimento ficaram abaixo do recomendado, ou seja, muito ácidos, refletindo na cifra diferencial dos couros, na formação de ácidos fortes livres nas amostras analisadas. O pH recomendado para o couro ao término do processo de curtimento deve ser de no 3,5 (ABNT NBR 13525, 2016). Nessa faixa de pH ocorre melhor fixação de corante e dos óleos utilizados na etapa de engraxe. Neste experimento os couros com sais de cromo e corante químico apresentaram o pH, mais próximo do recomendado (3,06) (Tabela 3).

Para todos os tratamentos observa-se que a cifra diferencial foi muito elevada (0,74 a 0,98), sendo o indicado de no máximo 0,7 (HOINACKI, 1989; ABNT NBR 13525, 2016). A acidez excessiva leva a degradação da cadeia proteica, pela hidrólise ácida, diminuindo a resistência do couro. Portanto, o que está dentro do recomendado são os couros curtidos com tanino vegetal e tingido com urucum (0,74) (Tabela 3).

Para as Substâncias Extraíveis com Diclorometano consta na Tabela 3 o percentual com uma variação de 6,13% a 18,62%, sendo o maior teor observado para os couros curtidos com sais de cromo e tingidos com urucum e o pior com sais de cromo e tingido com corante químico (6,13%).

Tabela 3. Testes físico-químicos dos couros de Pirarucu curtidos com tanino vegetal ou sais de cromo e submetidos ao tingimento com corante vegetal (urucum) ou químico.

Agente curtente	Óxido de Cromo Cr_2O_3 (%)	pH	Cifra Diferencial*	Substâncias Extraíveis com Diclorometano (%)	Determinação de Cálcio em couros **
Tanino x urucum	0,0	2,88	0,74	12,35	0,02
Tanino x químico	0,0	2,77	0,85	11,60	0,01
Sais de cromo x urucum	0,49	2,78	0,98	18,62	0,12
Sais de cromo x químico	0,28	3,06	0,98	6,13	0,28
Norma/Método	IT10.4-009	IT10.4-008	IT10.4-008	IT10.4-007	IT1.4-008

* A cifra diferencial somente age como um critério para a presença de ácidos fortes livres ou bases em extrato aquoso com valores de pH abaixo de 4,0 ou acima de 10.
**método colorimétricoutilizando espectro UV Cary 1E ou Cary 100

Os óleos adicionados no processo de curtimento agem no deslizamento das fibras colágenas fazendo com que elas se deslizem umas sobre as outras à medida que é submetida a algum tipo de tração, proporcionando uma maior mobilidade ou elasticidade para os couros de peixe (Souza, 2007). De acordo com a literatura as substâncias extraíveis em diclorometano devem estar no máximo entre 16% a 18% para o couro de bovino ser utilizado em vestuário. Há controvérsias para esse parâmetro, sendo assim, apenas os couros curtidos com sais de cromo e tingidos com corante vegetal (urucum) poderiam ser utilizados para a confecção de vestuário (Tabela 3). Segundo a ABNT NBR 13525 (2016) os couros para vestuário devem apresentar o valor médio de 2% de substâncias extraíveis em diclorometano.

É interessante que o couro apresente a maior resistência aos testes físico-mecânicos, com o mínimo de sais de cromo utilizados no processo de curtimento ou ausência desses sais.

A análise de determinação de cálcio dos couros curtidos com sais de cromo mostra que provavelmente a quantidade de cal adicionada na etapa de caleiro não tenha sido removida adequadamente na etapa de desencalagem (Tabela 3). Quanto menor quantidade de cálcio presente no couro, menor possibilidade dele engessar ou encartonar.

3.5.3 Colorimetria em Couros de Pirarucu Curtidos com Tanino Vegetal e Sais de Cromo e Submetidos ao Tingimento com Corante Químico e Natural

O agente curtente (tanino vegetal ou sais de cromo) não influenciou na luminosidade dos couros curtidos, no entanto, quando analisado o tipo de corante químico ou natural

(urucum), a luminosidade apresentou diferença significativa. O urucum proporcionou uma maior luminosidade, maior vivacidade da cor, maior brilho (Tabela 4). O parâmetro L* (luminosidade) variou de 49,54 a 59,15.

Os agentes curtentes interferiram apenas no croma a*, que determina a coloração indo para o vermelho. Os couros curtidos com tanino vegetal apresentaram um croma a* significativamente maior (24,40) que os couros curtidos com sais de cromo (20,47), mostrando assim uma maior tonalidade para a cor vermelha quando o couro foi curtido com tanino vegetal (Tabela 4).

Tabela 4. Análise colorimétrica em couros de pirarucu curtidos com tanino vegetal e sais de cromo submetidos ao tingimento com corante químico e vegetal com base no sistema.

	Curtente		Média
	Tanino	Cromo	
Luminosidade (L)			
Químico	49,64± 1,14	57,55± 2,84	**53,60 B**
urucum	59,15± 3,19	55,64±2,50	**57,39 A**
**Média**	**54,39 A**	**56,60 A**	
Trat		0,17	
Cor		0,03	
TratxCor		0,0045	
Croma a*			
Químico	25,71± 0,75	20,38± 1,82	**23,04 A**
urucum	23,10± 0,33	20,56± 0,92	**21,83 A**
**Média**	**24,40 A**	**20,47 B**	
Trat		0,0003	
Cor		0,09	
TratxCor		0,60	
Croma b*			
Químico	22,13± 1,04	24,75± 0,85	**23,44 A**
urucum	25,35± 1,15	23,91± 0,77	**24,63 A**
**Média**	**23,74 A**	**24,33 A**	
Trat		0,32	
Cor		0,07	
TratxCor		0,0065	

*médias e desvio padrão seguidas de mesma letra maiúscula na coluna (linha) não diferem ao teste de tukey a 5% de probabilidade.

Já a natureza do corante (químico e natural) não influenciou na cromaticidade a* e b* (Tabela 4). Os parâmetros colorimétricos para as alterações na cor da flor do couro do Pirarucu foram avaliados quanto ao envelhecimento acelerado e solidez a luz UV, por 72 horas a 100°C,

e quanto ao manchamento do tecido, quando submetido à solidez da cor à agua e solidez da cor ao suor nos tecidos cotton, poliamida, poliéster, acetato, acrílico e lã (Tabela 5).

Observa-se que o uso do cromo como curtente ou do tanino precedido de um corante químico apresentam ação mais agressiva para com as fibras colágenas. O corante químico, independente do curtente (cromo ou tanino) utilizado, proporcionou maior envelhecimento acelerado do couro e maior solidez da cor a luz UV (P<0,05).

A solidez da cor a ação do suor foi maior (P<0,05) para os couros curtidos com cromo e corados com urucum, independente do tecido utilizado (Tabela 3). Em contrapartida, a solidez da cor à àgua foi menor (P<005) para couros submetidos a curtente e corante químicos nos tecidos cotton poliamida, acetato e lã.

Tabela 5. Dados médios da solidez da cor do couro à luz UV, à água e ao suor de couro de Pirarucu curtido com sais de cromo ou tanino vegetal e corado com urucum ou corante químico.

Variáveis	Cromo e químico	Cromo e urucum	Tanino e urucum	Tanino e químico	Valor-P	CV (%)
Envelhecimento acelerado	4.5a	1.0b	1.5b	4.0a	<0.0001	5.8
Solidez a Luz UV	4.5a	2.29b	3.64a	4.4a	<0.0301	10.2
Solidez da cor à água						
Tec. Acetato	2.0a	4.0b	2.71a	4.08b	<0.0001	5.5
Tec. Cotton	1.5a	3.5b	3.07b	3.00b	<0.0001	8.1
Tec. Poliamida	1.0a	4.0b	3.5b	3.58b	<0.0001	10.6
Tec. Poliéster	3.5	4.5	4.0	4.08	<0.0001	6.6
Tec. Acrílico	3.0	4.5	4.5	4.08	<0.0001	8.4
Tec. Lã	1.0a	4.5b	4.0b	3.0b	<0.0267	12.2
Solidez da cor ao suor						
Tec. Acetato	1.0a	4.0b	2.50a	2.83a	<0.0001	5.5
Tec. Coton	1.5a	3.5b	2.14a	1.33a	<0.0001	8.1
Tec. Poliamida	1.0a	3.86b	2.36a	2.83a	<0.0001	10.6
Tec. Poliéster	2.0a	3.86b	2.43a	2.83a	<0.0001	6.6
Tec. Acrílico	2.0a	3.86b	2.43a	2.83a	<0.0001	8.4
Tec. Lã	1.0a	4.5b	3.86b	2.33a	<0.0267	12.2

*médias seguidas na mesma linha ± desvio padrão e teste de tukey a 5% de probabilidade; ** C.V. = coeficiente de variação

O tingimento é um processo empregado para conferir coloração desejada e melhorar o aspecto dos couros. Nesse processo, são usados substâncias corantes com características de possuir cor e se fixar ao substrato. Nesta etapa, como nas anteriores, devem ser favorecidas as condições de difusão e fixação. No processo de tingimento atuam afinidades ou repulsões das cargas tanto do couro quanto do corante. A reatividade entre eles será maior ou menor

dependendo da diferença das cargas do couro e o corante.

A radiação ultravioleta e calor são mais prejudiciais ao couro sem cromo que para o couro curtido ao cromo, especialmente com respeito à resistência de cor de corantes e às propriedades mecânicas (CHENG-KUNG LIU, et. al. 2006.). O papel da umidade e sua interação com radiação UV e temperatura em propriedades de couro não está claro à indústria de couro.

Os couros submetidos ao corante de urucum da Amazônia apresentaram grande eficácia na solidez da cor à água e ao suor, sem pré-tratamentos de corpos não lixados, apresentou-se pouco sensível ao manchamento em todos os testes com o corante natural de urucum, portanto, a utilização de corante natural não teve interferência visual no produto final.

O efeito dos corantes pode ser potencializado pelo curtente utilizado, porém, o tingimento com urucum reduz (P<005) o envelhecimento acelerado do couro e dá maior eficácia quanto a solidez da cor ao suor e demais intempéries, independente do tecido, mesmo quando precedido por curtente químico, características que dão maior maleabilidade e resistência na produção de produtos de confecções e artefatos com couro de Pirarucu.

CONCLUSÕES

O efeito dos corantes pode ser potencializado pelo curtente utilizado, porém, o tingimento com urucum reduz o envelhecimento acelerado do couro e dá maior eficácia quanto a solidez da cor ao suor e demais intempéries, independente do tecido, mesmo quando precedido por curtente químico, características que dão maior maleabilidade e resistência na produção de produtos de confecções e artefatos com couro de Pirarucu.

REFERÊNCIAS

ASSOCIAÇÃO BRASILEIRA DE NORMAS TÉCNICAS. NBR 3377-1: Ensaios físicos e mecânicos: Determinação da força de rasgamento. Parte 1: Rasgamento de extremidade simples. Rio de Janeiro, 2014. p.1-4.

ASSOCIAÇÃO BRASILEIRA DE NORMAS TÉCNICAS NBR 11041**:** couros – determinação da resistência à tração e alongamento. Rio de Janeiro, 1997. p.1-5.

ASSOCIAÇÃO BRASILEIRA DE NORMAS TÉCNICAS. NBR 11055: couro - determinação da força de rasgamento progressivo. Rio de Janeiro, 1997. p. 1-4.

CRUZ, W.D. **Características morfométricas e o efeito do peso de abate no rendimento de processamento do pirarucu** (*Arapaima gigas* CUVIER, 1817) **em piscicultura comercial no estado de Goiás**. 2012. 49 f. Dissertação (Programa de Pós-Graduação STRICTO SENSU em Tecnologia em Aquicultura Continental) - Pontifícia Universidade Católica de Goiás, Goiânia - GO.

GOUVEIA, J. P. G.; MOURA, R. S. F.; MEDEIROS, B. G. S. Determinação de algumas propriedades físicas das sementes de urucum. **Revista Brasileira de Corantes Naturais**, n. 4, p. 35-38, 2000.

HILBIG, C. C; FOCKINK, D. H; CAMARGO, M. et al. Resistência da pele de rã-touro (Rana catesbeiana) curtida com sais de cromo e tanino vegetal. In: II Simpósio Nacional de Engenharia de Pesca, IV Simpósio Paranaense de Engenharia de Pesca e XII Semana Acadêmica de Engenharia de Pesca, Toledo. **Anais...** II SINPESCA, 2010.

IBGE – Instituto Brasileiro de Geografia e Estatística. Diretoria de Pesquisas, Coordenação de Agropecuária, Pesquisa da Pecuária Municipal 2015/2016. **Produção Pec. Municipal**, Rio de Janeiro, v. 43, p.1- 49, 2015. Disponível em: <biblioteca.ibge.gov.br>. Acesso em: 20 Dez. 2017.

KIOKIAS, S.; GORDON, M. H. Antioxidant properties of annatto carotenoids. **Food Chemistry**, n. 83, p. 523-529, 2003.

NAJAR, S. V.; BOBBIO, F. O.; BOBBIO, P. A. Effects of light, air, anti-oxidants and prooxidants on annatto extracts (*Bixa orellana*). **Food Chemistry**, v. 29, n. 4, 1988.

SOUZA, M. L. R; OLIVEIRA, L. S. Efeito de técnicas de recurtimento sobre a resistência do couro da tilápia do Nilo (*Oreochromis niloticus* L.). **Acta Scientiarum Animal Sciences**, v. 27, n. 4, 2005.

CONSIDERAÇÕES FINAIS

A técnica de curtimento com tanino vegetal e o uso dos corantes naturais (urucum) em substituição dos sais de cromo e corantes tradicionalmente utilizados pelas indústrias de curtimento influenciaram na resistência dos couros de pirarucu (*Arapaima gigas*).

A utilização de tanino vegetal proporciona uma maior espessura para o couro e quando tingido com corante natural apresenta uma maior resistência ao rasgamento progressivo, porém quando utilizado o corante químico menor resistência para esse mesmo parâmetro. O uso do urucum reflete na elasticidade dos couros, tornando-os menos elástico, com menor deformação. Para a tração os couros curtidos com tanino e tingidos independente do tipo de corante apresentam a mesma resistência que os couros curtidos e tingidos com corante químico. O urucum proporciona maior luminosidade ao couro e o tanino vegetal o maior croma a*, tendendo a cor vermelha. Portanto, é mais interessante o uso do curtente e corante vegetal para usar no processamento dos couros de pirarucu.

O tanino vegetal proporciona um couro 24% mais espesso; 34,78% mais resistente à aplicação de força; 3,21% mais resistente a tensão à tração e 10,4% mais elástico.

A combinação tanino vegetal e urucum preservou a integridade das fibras colágenas com maior espessura do couro (2,10mm vs. 2.61mm) e demandando mais força (178,2N vs. 125,3N), para danos físico-mecânicos. As médias para a força de tensão á tração foi de 6,38N/mm², rasgamento 30,3mm, alongamento 50,6% e rasgamento de 40,0N/mm. O efeito dos corantes pode ser potencializado pelo curtente utilizado, porém, o tingimento com urucum reduz o envelhecimento acelerado do couro e dá maior eficácia quanto a solidez da cor ao suor e demais intempéries, independente do tecido, mesmo quando precedido por curtente químico, características que dão maior maleabilidade e resistência na produção de produtos de confecções e artefatos com couro de Pirarucu.
